# Factors influencing antimicrobial resistance in the European food system and potential leverage points for intervention: A participatory, One Health study

**DOI:** 10.1371/journal.pone.0263914

**Published:** 2022-02-22

**Authors:** Irene Anna Lambraki, Melanie Cousins, Tiscar Graells, Anaïs Léger, Patrik Henriksson, Stephan Harbarth, Max Troell, Didier Wernli, Peter Søgaard Jørgensen, Andrew P. Desbois, Carolee A. Carson, Elizabeth Jane Parmley, Shannon Elizabeth Majowicz

**Affiliations:** 1 School of Public Health Sciences, University of Waterloo, Waterloo, Ontario, Canada; 2 Global Economic Dynamics and the Biosphere, Royal Swedish Academy of Sciences, Stockholm, Sweden; 3 Stockholm Resilience Centre, Stockholm University, Stockholm, Sweden; 4 Global Studies Institute, University of Geneva, Geneva, Switzerland; 5 Beijer Institute of Ecological Economics, Royal Swedish Academy of Sciences, Stockholm, Sweden; 6 WorldFish, Jalan Batu Maung, Batu Maung, Penang, Malaysia; 7 Infection Control Programme and WHO Collaborating Centre on Patient Safety, Geneva University Hospitals and Faculty of Medicine, Geneva, Switzerland; 8 Institute of Aquaculture, University of Stirling, Stirling, United Kingdom; 9 Centre for Food-borne, Environmental and Zoonotic Infectious Diseases, Public Health Agency of Canada, Guelph, Ontario, Canada; 10 Department of Population Medicine, Ontario Veterinary College, University of Guelph, Guelph, Ontario, Canada; Bangladesh Agricultural University, BANGLADESH

## Abstract

**Introduction:**

Antimicrobial resistance (AMR) is a global crisis that evolves from a complex system of factors. Understanding what factors interact is key to finding solutions. Our objective was to identify the factors influencing AMR in the European food system and places to intervene.

**Materials and methods:**

We conducted two workshops involving participants with diverse perspectives to identify the factors influencing AMR and leverage points (places) to target interventions. Transcripts were open coded for factors and connections, then transcribed into Vensim 8.0.4 to develop a causal loop diagram (CLD) and compute the number of feedback loops. Thematic analysis followed to describe AMR dynamics in Europe’s food system and places for intervention. The CLD and themes were confirmed via participant feedback.

**Results:**

Seventeen participants representing human, animal and agricultural sectors identified 91 CLD factors and 331 connections. Seven themes (e.g., social and economic conditions) describing AMR dynamics in Europe’s food system, five ‘overarching factors’ that impact the entire CLD system (e.g., leadership) and fourteen places for intervention (e.g., consumer demand) emerged from workshop discussions. Most leverage points fell on highly networked feedback loops suggesting that intervening at these places may create unpredictable consequences.

**Conclusions:**

Our study produced a CLD of factors influencing AMR in Europe’s food system that implicates sectors across the One Health spectrum. The high connectivity between the CLD factors described by participants and our finding that factors are connected with many feedback mechanisms underscores the complexity of the AMR problem and the challenge with finding long-term solutions. Identifying factors and feedbacks helped identify relevant leverage points in the system. Some actions, such as government’s setting AMU standards may be easier to implement. These actions in turn can support multi-pronged actions that can help redefine the vision, values and goals of the system to sustainably tackle AMR.

## Introduction

Antimicrobial resistance (AMR) claims 700,000 lives globally and threatens health, social and economic well-being [1–3], with antimicrobial use (AMU) a widely recognized cause [4, 5]. AMR in food animals can spread through the food chain contributing to resistant infections in people [6–8]. Trade, travel, and waste also contribute to AMR spread across borders and into the environment [9, 10]. Because AMR implicates many sectors, it is an all-of-society problem that requires a One Health approach [11], including broad engagement of actors from different sectors, to better understand contributing factors and find sustainable solutions beyond what traditional experts have unearthed [12].

Because many types of actors are involved in AMR, tools that can integrate knowledge about how their actions directly or indirectly impact AMR or are impacted by AMR are needed. Causal loop diagrams (CLDs) are a tool used in system science to integrate the diverse perspectives of actors from different sectors and model the range of factors that may impact complex systems. CLDs are visual models that illustrate the relationship between explanatory factors and outcomes [13–15]. In addition to organizing and analyzing information to provide a whole picture of the factors within a system, CLDs enable the identification of leverage points or places where interventions may transform system behaviour in ways that mitigate a problem [16] and feedback loops to determine which leverage points may have greater or less potential for creating chaos and unpredictable consequences in the system.

While drivers of AMR have been identified, greater understanding about how micro and macro factors interconnect to generate AMR is needed. Thus, our objectives were to gain insights from diverse perspectives to understand the broad range of factors influencing the development and spread of AMR in the food system and identify leverage points for intervention with potential to effectively address AMR long-term. We successfully conducted our study with a focus on Europe.

## Materials and methods

This study used qualitative methods. We followed the Consolidated Criteria for Reporting Qualitative Research (COREQ) checklist for reporting our qualitative research [17]. This study received ethics clearance from the University of Waterloo’s Research Ethics Committee (ORE# 40519). All participants provided written consent to participate in the study and for the use of anonymous quotations. Thus, quotations that are provided (in the results section and in S1–S3 Tables) are identified based on which workshop day it was stated in accordance with research ethics requirements.

### Research team and reflexivity

The study was designed and conducted by a core team (co-authors: SE Majowicz (SEM); EJ Parmley (EJP), IA Lambraki (IAL), M Cousins (MC), CA Carson (CAC)) who consulted with a larger interdisciplinary project team of academics with specialties in human medicine, aquaculture, clinical microbiology, and evolutionary biology, throughout the design, data collection and analysis steps. The core team has disciplinary backgrounds in public health, epidemiology, and veterinary medicine and bring experience with qualitative methods, systems thinking, food safety, and antimicrobial resistance.

### Study design

Using a participatory approach and systems thinking, we engaged stakeholders from across the One Health spectrum in two group model-building workshops to develop an interdisciplinary CLD relating to AMR in the European food system (S1 Fig). We defined the European food system as encompassing all actors and actions involved from farm to fork and the disposal of food products originating from agriculture, fisheries, and parts of the broader natural, social and economic environments in which they are embedded. Our definition also includes other systems (health, trade, environment) that connect with and may impact the food system [18]. We focused on the European food system because it involves European countries that belong to and operate under the regulations of the European Union and non-European Union countries, which has created varying levels of AMR [19] with some areas such as the Nordic countries being more successful at tackling AMR [4]. This renders Europe a rich context to explore the broad system of factors that may impact AMR.

### Participant selection and characteristics

Participants were identified through: Google, LinkedIn, and Twitter searches; professional organizations’ websites that may impact AMR in human, animal, agricultural, and environment sectors; and via the research team’s (IAL, MC, CAC, EJP, SEM, and T Graells (TG), A Léger (AL), P Henriksson (PH), M Troell (MT), S Harbarth (SH), D Wernli (DW), and P Søgaard Jørgensen (PSJ) professional networks who identified individuals to approach that represented expertise that is less traditionally engaged in AMR discussions. We purposively selected participants with diverse perspectives from Europe, with either traditionally recognized expertise in AMR (e.g., medical and veterinary doctors, researchers), or expertise in areas that may directly or indirectly impact AMR (e.g., peace and conflict resolution; leadership). Efforts were made to recruit participants across human, agriculture (farming of terrestrial livestock, aquaculture, and crops), and environment sectors.

We approached 64 participants via email with a maximum of two follow-up contacts in alignment with the University of Waterloo Ethics Committee approved protocols. Twenty-six participants did not respond. Twenty-one individuals declined due to work conflicts, AMR not being deemed a priority for the approached participant’s organization, or the individual did not see their role in AMR relevant to the food system. Seventeen participants agreed to participate. Almost half of the participants were female. Participants represented the following perspectives: epidemiology, food safety and microbiology, veterinary sciences, aquatic sciences and aquatic foods, agricultural crops and policy, animal welfare, human medicine, nursing, public health, public health advocacy, consumer advocacy, pharmaceutical marketing, pharmaceutical law, trade and economics, urban agricultural innovation, sustainable foods and innovation, dietetics, peace and conflict resolution, and leadership. These participants represented organizations at sub-national, national, or regional (i.e., European) levels, including governmental and non-governmental organizations, health care organizations, private consultants, and industry. Over half of the participants were from Sweden, and the remaining from France, Italy, Spain, United Kingdom, and Belgium. Participants did not have previous relationships with research team members.

### Data collection and analysis

We held two in-person 6.5-hour workshops over two days: September 19^th^ and 20^th^, 2019 at the Stockholm Resilience Centre (Stockholm, Sweden). Both workshop days had a mix of AMR experts and experts in other content areas relevant to the food system that may not usually be engaged in discussions about AMR. However, workshop day one brought together participants that could provide a broad understanding of AMR in the European food system context, and thus had a greater representation of AMR experts. Workshop day two brought together participants that could provide an understanding of sectors in the food system at a national and sub-national level which may influence AMR directly or indirectly, and thus had a greater representation of experts in content areas that may not usually be engaged in AMR discussions and varying levels of knowledge about AMR.

The purpose of the workshops was to have participants with diverse perspectives brainstorm the system of factors that may influence AMR and identify where in that system to intervene. Workshops were audio-recorded, guided by a semi-structured interview guide, and facilitated by our team (IAL, EJP; note taker: MC). Co-authors (AL, TG, PH, MT, PSJ, DW) attended the workshops to listen and provide expert input if requested by participants. Each workshop started with a welcome, icebreaker activity, overview of the workshop objectives, and a brief presentation on AMR to provide a common understanding and terminology. A practice system thinking activity followed, in preparation for the modelling process.

To initiate conversations, participants were introduced to an existing CLD of AMR in the Canadian food system [20], and tasked to adapt the CLD to reflect the European food system. We defined factors influencing AMR to be any factors associated with AMU, AMR, or AMR impacts, either proximately or distally. Facilitators added or removed factors and connections and changed the names of factors in the CLD as directed by participants; this was done directly on the starting CLD. Facilitators asked participants if they were describing antibiotic resistance or AMR in general when unclear. Prior to adding a given factor or connection to the CLD, participants were asked if they wanted to question, counter, revise or add anything to the point made by another participant. Each factor was written as a short textual phrase. Participants were prompted to frame factors as ‘measurable’ to enable future simulation modelling (e.g., ‘amount of exported food products’ versus ‘exported food products’). Facilitators also elicited the direction, and where possible, the nature of the connections between factors. The direction of connections between factors was depicted by an arrow (→), and where participants identified it, a positive (+) or negative (-) sign on the arrow was used to identify the nature of the connection. If participants did not identify the nature of the relationship between factors, then no sign was added to the arrow. A positive connection indicates that two factors are moving in the same direction (i.e., more ‘good farming practices’ lead to improved ‘animal welfare’). A negative connection indicates that two factors move in opposite directions (an increase in ‘non-antimicrobial infection control on farms of food-producing animals’ (e.g., vaccination) leads to a reduction in ‘food-producing animal illness’). Then, through small and in turn large group discussions, participants pinpointed which factors could be leverage points for intervention with potential to change the behaviour of the system in ways that sustainably address AMR and suggested actions for each identified leverage point. Throughout the workshop process, revisions and discussions with the larger group continued until participants had no new information to share and participants indicated that they were comfortable with the CLD and that the CLD and identification of leverage points were complete.

The workshops produced five data sources that were analysed: the facilitator-revised CLDs (S2 and S3 Figs); each participant’s own marked-up handout of the starting CLD; verbatim workshop transcripts; flip chart and meeting notes. From these sources, and for each of the two workshops separately, co-author (IAL) open coded data using NVivo 12, triangulated data sources and conducted the thematic analysis, and co-author (MC) extracted all factors and connections and entered them into Vensim Professional 8.0.4, to yield two CLDs of AMR in the European food system. Co-authors (MC, SEM EJP, CAC, IAL) met at key points throughout the analysis process to discuss the two workshop findings, combine the two CLDs because of similarities, and to finalize any areas of uncertainty about factors and connections (e.g., their placement in the CLDs). Participants were sent a summary report of workshop themes, leverage points and associated suggested actions, and the draft CLD (with factors common to both workshops, or specific to one workshop, distinguished) for member checking, and participants provided feedback to ensure materials accurately reflected their understanding of workshop discussions. Nine of the 17 participants provided feedback, which included: agreement that the summary report and CLD reflected the workshop discussions; a request to make one change; requests to share the outputs with working groups; a request to be connected to another participant with expertise in a particular area to ask questions; and to thank the research team for the opportunity to take part in the workshop and express that they found the experience interesting. Participant feedback was incorporated to produce the final CLD of AMR in the European food system, for which the number of feedback mechanisms for each CLD factor were computed in Vensim. Feedback loops occur when a CLD factor is both an input and outcome of some causal process and a change in one factor can spark a circular chain of influence that either increases or reduces the original factor. The number of feedbacks therefore, provide a measure of the complexity of AMR and the extent of challenge it is to sustainably mitigate AMR.

The research team classified the leverage points for intervention that participants identified as ‘shallow’ (places where interventions are easier to implement with less potential to transform the system) or ‘deep’ (places in the system that are more difficult to alter yet have greater potential for transformational change) per Meadows [16]. Then we examined whether the identified leverage points were part of highly interconnected feedback mechanisms or not to determine if intervening at each leverage point has a greater or lesser potential to create unintended consequences in the system that could positively or negatively impact efforts to mitigate AMR or other parts of the system.

## Results

**Fig 1:**
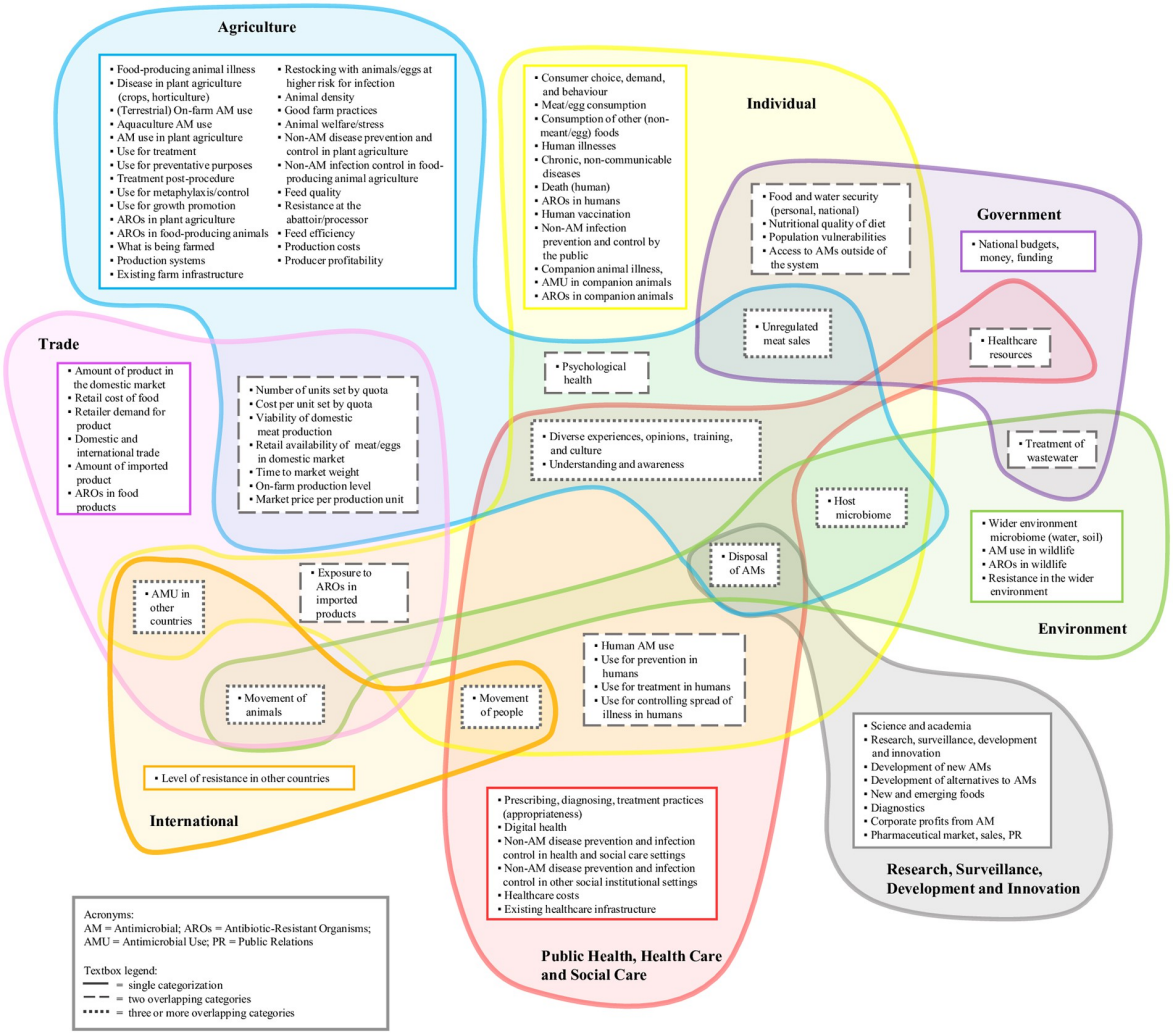
91 CLD Factors by 8 categories.

The CLD of AMR in the European food system contained 91 factors and 331 connections (S4 Fig); for ease of presentation, we grouped the 91 factors into eight categories (Fig 1) and defined them (S4 Table). Participants also identified five additional ‘overarching factors’ not included in the CLD because they impact the entire system: ‘agreements, standards, and regulations’; ‘leadership’; ‘media’; ‘collaboration’; and ‘climate change’. Seven themes emerged from workshop discussions (Table 1) that describe key AMR dynamics illustrated in the CLD.

**Table 1:** Description of the seven themes.

Theme	Description of Theme
**AMU and AMR spread**	Describes human behaviours that can contribute to AMR spread in the environment, such as: AMU; the disposal of antibiotics, and waste from humans, animals, and industry (e.g., pharmaceutical industry); and trade and travel.
**Economics and agricultural/aquaculture practices**	Describes the role of the market-driven economy in driving industry to higher density farming and AMU, and how changes to farming production and management practices could impact producers’ ability to cover the associated costs and remain viable.
**Consumer choice, demand, and behaviour**	Focuses on the impact of consumer demand for products and services (e.g., food; hospital) on AMU, and the factors (e.g., habits, personal experiences) that shape these demands.
**Health and social care systems**	Focuses on how prescribing practices, resources and capacity issues of the animal and human health and social care systems (public health, health care including veterinary services, social care and the communities they serve) can impact whether or how well antimicrobial stewardship and infection prevention and control measures to address AMR are carried out.
**Promoting health and well-being**	Focuses on how promoting psychological well-being and human and animal health can contribute to AMR mitigation efforts.
**Social and economic conditions**	Identifies the impacts that social and economic inequities exert on AMR, health and well-being.
**Research**	Focuses on the importance of research to develop new antimicrobials and the need for new sustainable funding models to incentivize efforts in these areas, alternatives to antimicrobials, new antimicrobial-free foods, and surveillance needs.

### Themes

#### AMU and AMR spread

Participants identified antimicrobial misuse in food production (terrestrial livestock, aquatic animal farming, and plant agriculture) and in human health care as a major driver of AMR, which contributes to the number of antimicrobial-resistant organisms (AROs) in the environment. Antibiotics and antibiotic residues that get released into the environment (e.g., through effluent from farms, healthcare facilities, and the pharmaceutical industry) were identified as contributing to the development, frequency and spread of AROs in the environment (e.g., via water). Human and animal excreta were also noted to spread AROs and residues, such as when faecal waste and wastewater are recycled as manure in agri- or aquaculture. All these practices were noted to spread AMR to animals (aquatic, terrestrial, wildlife, companion, and livestock animals), plants, and humans (e.g., through contact with waterways). Participants also identified wastewater treatment facilities as impacting AMR spread, noting them as “…*a major hot spot for the selection of resistant organisms…”* (workshop Day 1), depending on standards. AMU in aquaculture was described as an under-examined source for AMR development and spread through water. Other modes of AMR spread identified were abattoirs (via employee contact with affected livestock), animal transport for trade, and the movement of people due to migration and health tourism.

#### Economics and agricultural/aquaculture production practices

Economics was a motivator for antibiotic use in animal farming. Participants identified the market-driven economy as driving industry to higher density farming practices to meet demands and generate profit, which increases animal stress, illness, and antibiotic use (e.g., for metaphylaxis purposes) as a cost-effective intervention. Good farming practices and non-antimicrobial infection prevention and control (IPC) measures (e.g., vaccinations, nutritious feed, animal housing) in terrestrial and aquaculture food production (e.g., on-farm, abattoirs) were identified as necessary to improve animal welfare, reduce illness, and limit the need for antibiotics. Technologies that treat and repurpose existing waste (e.g., human) by extracting nutrients for use in fertilizer on crops and feed in animals were also described as having potential to contribute to good farming practices by improving nutrition and the health of animals. Implementing good farm management practices, however, was identified as requiring training (e.g., focused on farmers, seasonal workers, abattoir staff) and incurring “…*big costs upfront…*” to farmers who already operate with a “*low margin…*”(workshop day 1).

#### Consumer demand

Demand for products and antibiotics by individuals was perceived to influence antibiotic use. Participants highlighted that consumer demand impacts the types of products retailers bring into the marketplace and increases food production involving AMU (see theme: Economics and agricultural/aquaculture practices). Consumer demand for less expensive food was also said to influence the purchasing of imported meat that may be produced using unregulated AMU or that may contain unknown levels of ARO loads due to the absence of testing at borders (see theme: Agreements, standards and regulations). However, participants noted that consumer willingness to pay more for niche markets could shift food production practices and reduce AMU. Religious factors and an individual’s experience with disease or AMR were said to shape consumer demand and willingness to pay more for food products produced without antibiotics, such as kosher meat or organic food. Consumers’ level of understanding of where food comes from and how it is produced was noted to potentially shape consumer demand. In Sweden for instance, a high level of awareness about food and the importance of sustainable practices was described to increase consumer demand for foods raised with good farm practices and higher animal welfare standards.

Beyond food, participants identified how patients and companion animal owners may demand and use antibiotics when not indicated, potentially reducing the efficacy of existing antimicrobials. Human’s search for a “*quick fix*” (workshop day 2) and cultural norms were said to drive adults to demand antibiotics to avoid using sick days to meet work productivity standards. Previous experiences were also identified such as immigrant families demanding antibiotics at first sign of mild colds because “….*that mild cold in their own country can kill that child*” (day 2 workshop). Family members purchasing antibiotics while travelling “…*because it’s easier [to access] than in the country they live in*” (workshop day 1) and passing them on to family and friends was another factor influencing AMU or inappropriate AMU and potential development of AMR. High previous antibiotic exposure was noted to influence an individual’s resistance and in turn the potential effectiveness of treating infections with antibiotics. Unpacking the individual, social, economic, and policy factors that influence consumer choices was identified as necessary to developing effective behaviour change interventions.

#### Health and social care systems

Participants described the presence or absence of antimicrobial stewardship (e.g., use of diagnostics, antibiotics under prescription and IPC measures) in human and animal health care settings, social care settings (e.g., home care, long-term care facilities) and the communities they serve as impacting AMU and AMR spread (e.g., via disposal of antimicrobials and waste and IPC measures), the burden of illness, and health and social care costs. Several factors were identified as impacting stewardship, such as pressures on physicians and veterinarians to prescribe antibiotics (e.g., patient demand, physician fear of causing patient harm by not prescribing, potential negative media exposure, legal problems by not prescribing, and pharmaceutical industry interactions with medical professionals that may involve economic incentives to prescribe). Technological advancements that have led to increased digitization in health care were also described as increasing physicians’ reliance on lab results to diagnose rather than spending adequate physician-patient time to make a holistic assessment of symptoms, potentially impacting early accurate diagnosis and antibiotic prescriptions. Participants also noted that little is known about how technologies such as “*wearable devices*” (workshop day 2) and rising e-health solutions (e.g., ePrescriptions, online medical consultations) will impact prescriptions or human behaviour. Resource issues, such as high costs of diagnostic kits that reduces their use and drives prescription of broad-spectrum antibiotics, insufficient physicians per population that reduce physician-patient consultation time and increase the potential for suboptimal diagnoses and antibiotic prescriptions, and a lack of adequate hospital IPC staff found in countries with significant AMR problems were also described as impacting stewardship. The movement of people (e.g., through migration, health tourism, and the influx of workers in agriculture and health care) was also noted to increase resource demands and capacity issues by introducing diverse opinions, trainings and cultures that may impact stewardship in human and agricultural contexts.

#### Promoting health and well-being

Health and psychological well-being were highlighted as important to preventing illness in animals and humans and reducing AMU and healthcare costs. Vaccinations were viewed as important to illness prevention although participants recognized efforts to increase vaccination in humans and animals in Europe have not translated into widespread uptake. Reducing stress and improving overall health status in animals via good farming practices and high animal welfare standards and improved lifestyle behaviours and nutritional intake in humans were identified as useful to cure some minor illnesses. Changing the environments in which we live to be more health promoting was also deemed important to reducing stress. Using narrow spectrum antimicrobials was also identified as important to treating illnesses. All these practices were also said to reduce AMU and preserve host gut microflora, which may build immunity and resilience to illness and reduce AMU. Promoting producer mental health was also identified as important to good farming practices, as farmer stress levels may translate into less care for others, including their animals.

#### Social and economic conditions

Social and economic conditions were identified as influencing AMR and health and psychological well-being. Participants noted that “*patterns of resistance are linked to social inequalities–to areas…that have deprivation…*” (workshop day 1), which may increase diseases and health and social care costs. Countries, populations and individuals experiencing poverty were said to be at greater risk of experiencing transient employment with no sick pay, food and water insecurity, compromised nutritional intake, and preventable chronic diseases, which contribute to stress and poorer psychological well-being, and health complications that may require antibiotic treatment compared to those with greater affluence.

#### Research

Participants identified many actions and approaches to tackle AMR. Participants noted that “*when it comes to antibiotics*, *we need the entire portfolio*, *so we need to find ways to both safeguard the existing…ones because we will need them also in the future*, *but also find of course new business models to come up with new antimicrobials…*” (day one workshop).” New sustainable funding models and incentives that are potentially funded by the government or through the support of public-private partnerships were viewed as needed to assist pharmaceutical companies in the costly development of new antimicrobials. Additional ways to protect the effectiveness of antimicrobials and tackle AMR were identified, such as exploring the gut microbiota and the microbiome to find alternatives to antibiotics and improve health and well-being; genomics research to advance personalized medicine; development of recommended antimicrobial lists for use in animals and humans plus rapid diagnostics that distinguish between different infections to inform treatment guidelines and responsible AMU; development of new foods (e.g., via antimicrobial-free 3-D printed foods and genetically modified foods); and surveillance of AMR spread in the environment, including via aquaculture. The need to get ahead of the AMR crisis by overcoming existing debates between sectors about the need for a strong evidence-base before acting versus acting when warranted to accumulate the evidence (e.g., the push for surveillance of AMR in the environment) was discussed. Better political alignment between government departments was also identified as important to increase research budgets and release funds allocated to different issues (e.g., the environment and health) for joint action “…*where…co-benefits exist*” (workshop day 1) (e.g., to examine climate change impacts on AMR).

### Overarching factors

‘Agreements, standards, and regulations’ were said to impact the entire CLD with beneficial and negative consequences. In Europe, agreements, standards, and regulations were noted to promote responsible AMU and limit AMR (e.g., the ban on antibiotic use for growth promotion) yet participants said surveillance systems to track the ARO load in imported food products was lacking. Moreover, participants described negative consequences due to regulations for farmers. For example, in some Northern European countries, the domestic animal-food industry was said to potentially spend more to comply with domestic restrictions to reduce AMU and certain bacteria in their food products, and are often outcompeted by less expensive products imported from other countries which produce animals or food under different conditions. Concern was also raised that strict import regulations in some Northern European countries may eventually motivate some producers to export their products to countries with growing economic purchasing power and less stringent regulatory requirements. Participants also highlighted that different European countries have varying levels of AMR, do not necessarily operate with the same regulations, and if they do, have different interpretations, which raised questions about “*how effective is it if you’re not using it [regulations] in one area and your neighbouring country is…*” (workshop day 1). For instance, participants said some European countries require a prescription for antibiotics while others allow antibiotics to be purchased over the counter. Moreover, participants noted (in)formal domestic and international agreements (e.g., trade, climate, security) “…*drive the political agenda in a certain way and they might not always be moving in the same direction*” (workshop day 1) potentially circumventing AMR mitigation efforts. Ensuring international agreements have restrictions on AMU in food animals including use of antimicrobial pesticides, and that code of practices or standards such as from Codex Alimentarius are implemented were noted by participants as important.

‘Leadership’ was viewed as strongly shaping the factors governing AMR. Participants noted that leaders could be anyone and that individuals and non-governmental organizations could play an important role in raising awareness and taking action to address the AMR problem. Governments were identified as key leaders because they set the vision and policies that can influence changes in how a nation or society operates. Moreover, participants noted the importance of leaders cultivating trusting and respectful relationships with others to develop a shared mindset about the ideal future and inspire action. Participants illustrated how, in Sweden, the government is generally viewed as a “*trustable system*” (workshop day 2) and citizens follow the rules because decision-makers have demonstrated their commitment to ensuring public well-being by achieving peace and affluence. Having “…*no wars in Sweden for …at least two hundred years [has given us] time to worry about the future*, *and to put energy…and financial resources into what is coming*” (workshop day 2). This context was said to have enabled Sweden to be forward-thinking (e.g., being one of the first countries to address AMR), and to take actions to deter corruption (e.g., through delinking industry interests from government decisions). Participants also described the need for leadership to set agendas and make legislative decisions based on scientific evidence rather than “…*the agenda [being] so directed by*, *or influenced by*, *some stakeholders*…” (workshop day 1).

‘Media’ and social media were said to drive information, either translating scientific evidence in appropriate or inappropriate ways, delivering “*fake news*” (workshop day 1) to the public, or framing messages to “*scare us*” (workshop day 2), with the power to shape public opinion that can help or hinder AMR mitigation efforts.

‘Collaboration’ within and between governments and industries was identified as an overarching factor because it impacts sharing of data about AMU and actions between sectors to tackle AMR, and this can be limited if there are fears from sectors (e.g., farming industry) of repercussions from this transparency.

‘Climate change’ was identified as an overarching factor. Participants described climate change and other parts of the system as “*interlinked in many ways*” (workshop day one). One way that participants said climate change would impact the system was via rising temperatures. A sector where climate change was described to be “*having a direct impact on AMR is in…salmon farming*, *where the season is now two degrees warmer*, *[and] the second you get even a degree warmer*, *you get more disease outbreaks because the animals are more stressed*” (workshop day one). These changes in turn were said to potentially increase the need for AMU for treatment and the emergence and spread of AMR, threaten food security, increase the retail cost of food, and negatively impact people and countries experiencing inequities in the social determinants of health as they will bear a disproportionate climate burden. Selecting or genetically modifying food-animal and crop species that are less vulnerable to climatic changes was described as potentially useful to shift consumer demand and potentially reduce the need for AMU in food production. Climate change was also described to impact water via, for instance, flooding, which could overwhelm wastewater treatment systems causing the potential spread of antimicrobial residues and AMR. Increasing research funding directed to AMR in the environment and in aquaculture was viewed as likely due to climate change. Participants also noted that differing levels of commitment across nations regarding achieving the Sustainable Development Goals was challenging society’s ability to address the climate crisis.

### Leverage points

Participants identified 14 factors (11 factors in the CLD, and three ‘overarching factors’) as leverage points with the potential to transform the system to combat AMR, and suggested associated actions, which we classified as shallow and deep leverage points per Meadows (1999) (Table 2).

**Table 2:** Shallow and deep leverage points, associated actions, and feedback loops.

Leverage Point	Associated Actions	Feedback Loops
***Shallow Leverage Points*: *Places where interventions are easier to implement with less potential to transform the system in ways that mitigate AMR*.**		
**National budgets, money, and funding (CLD factor)**	Increasing investments in: Providing universal health care. Developing health promotion and prevention agendas and initiatives to improve health and psychological well-being. Providing insurance for farmers (e.g., to cover costs to deal with infectious outbreaks, such as methicillin-resistant *Staphylococcus aureus* (MRSA). Developing new antibiotics using sustainable business models that delink volume sales from manufacturer reimbursement, and by incentivizing the innovation system through public-private partnerships to address AMR.	Involved in zero feedback loops, indicating that a change in the CLD factor ‘national budgets, money, and funding’ directly impacts other parts of the system (i.e., CLD factors ‘research, surveillance, development and innovation’ and ‘health care infrastructure’) that each go on to impact other parts of the system), but is not subsequently impacted by the system.
**Retailer demand for product (CLD factor)**	Changing what products retail companies offer in the marketplace by: Creating collective agreements that change procurement requirements for foods and products, which puts pressure on suppliers to change production practices and systems to support antimicrobial stewardship and good farming practices.	Involved in up to 32,766 feedback loops, with a given feedback loop length containing 1 to 30 other CLD factors, indicating that a change in the CLD factor ‘retailer demand for product’ heavily impacts and is heavily impacted by many parts of the system.
**Agreements, standards, and regulations (overarching factor)**	Addressing AMU and AMR by: Setting antimicrobial use limits (e.g., in medicated animal feed) across nations, and AMR limits in imported foods. Providing economic incentives to promote good AMU practices in human and animal sectors. Changing public procurement requirements to promote public institutions’ (e.g., schools) purchasing of foods that support AMR mitigation efforts. Creating regulations that ensure non-antimicrobial infection prevention measures in particular settings (e.g., on-farm and abattoirs to protect against MRSA). Creating regulations about how industry can work with decision-makers, such as regulations that ensure an independent national expert group evaluates all new alternatives to antimicrobials to avoid industry directly marketing these products to decision makers.	Not applicable because the overarching factor ‘agreements, standards and regulations’ impacts the system of factors in the CLD.
***Deep Leverage Points*: *Places where interventions are harder to implement yet have potential for transformative change to mitigate AMR***		
**Psychological Health (e.g., stress, producer mental health) (CLD factor)**	Increasing a focus on the prevention agenda to promote health and well-being and prevent disease by: Gathering data on and sharing the impacts of interventions that promote health and well-being and any unintended consequences.	Involved in zero feedback loops, indicating that a change in the CLD factor ‘psychological health’ directly impacts other parts of the system (i.e., CLD factors ‘population vulnerabilities’; ‘chronic non-communicable diseases’; and ‘good farming practices’ that each go on to impact other CLD factors), but is not subsequently impacted by the system.
**Understanding and awareness of scientific evidence, surveillance, and best practices (CLD factor)**	Moving scientific evidence, best practices, and success stories into action across sectors and nations to improve health and well-being and AMU practices, reduce AMR spread, and counter incorrect media messages by: Developing evidence-based AMU recommendations and providing training on these recommendations to front-line workforce across sectors (e.g., farmers and seasonal farm workers, health care and allied health care providers) to promote antimicrobial stewardship and good farming practices. Incorporating AMR, good health, and good farming practices in early school education to equip future generations with skills in stewardship and healthy living. Developing public campaigns on the benefits and risks of AMU for individuals versus the population. Marketing AMR as a One Health and One Welfare issue to create a movement for collective action like the climate change movement. Engaging media and social influencers to build relationships and their skills in delivering evidence-based messaging on AMR. Increasing advocacy among non-governmental organizations and influential figures to bring evidence-based messaging to political leaders that shape policy.	Involved in one feedback loop, indicating that a change in the CLD factor ‘understanding and awareness of scientific evidence, surveillance, and best practices’ directly impacts one other part of the system (i.e., CLD factor ‘science and academia’) and is subsequently impacted by that part of the system.
**Consumer Choice, Demand, and Behaviour (CLD factor)**	Shifting consumer and patient demand for products and services to transform widespread reliance on AMU via: Increasing transparency via labels describing animal welfare standards and the antibiotic footprint of products (e.g., food) or services (e.g., hospitals) to increase traceability, transparency, and consumer willingness to use or pay more for niche markets that could shift food production practices and put pressure on decision-makers for policy change that contribute to AMR mitigation efforts.	Involved in up to 32,766 feedback loops, with a given feedback loop length containing between 4 and 33 other CLD factors, indicating that a change in the CLD factor ‘consumer choice, demand and behaviour’ heavily impacts and is heavily impacted by many parts of the system.
**Non-antimicrobial disease prevention and infection control in plant agriculture (e.g., heavy metals).**	Improving non-antimicrobial approaches to prevent and control infections and diseases in different settings via: Sharing success stories, for instance, where on-farm investments in infection prevention measures did not significantly change cost structures. Improving good farming practices (e.g., increasing biosecurity, vaccinations, and animal welfare standards) to raise and keep food animals healthy. Improving the level of hygiene in human health care to prevent disease. Implementing evidence-based interventions and recommendations to prevent or control infection and AMR spread (e.g., advising people at high risk of MRSA to not work in swineries). Developing devices that notify health care providers to adhere to workplace infection prevention measures (e.g., wash hands, etc).	Involved in >30,000 feedback loops, with a given feedback containing upwards of 33 CLD factors, indicating that a change in each of the five CLD factors ‘non-antimicrobial disease prevention and infection control in plant agriculture’; ‘non-antimicrobial infection prevention and control by public’; ‘non-antimicrobial disease prevention and control in health and social care settings’; ‘non-antimicrobial infection prevention and control in other social institutions’; and ‘non-antimicrobial infection control on farms of food producing animals’ heavily impacts and is heavily impacted by many parts of the system.
**Non-antimicrobial infection prevention and control by public (e.g., hand hygiene, home cooking; social isolation; access to sick days).**		
**Non-antimicrobial disease prevention and control in health and social care settings (e.g., hospital and long-term care).**		
**Non-antimicrobial infection prevention and control in other social institutional settings (e.g., restaurants, workplace, community, home).**		
**Non-antimicrobial infection control on farms of food producing animals. (5 CLD factors)**		
**Research, surveillance, development, and innovation (CLD factor)**	Focusing research efforts in the following areas to fill knowledge gaps and explore (new) responses to AMR: Conducting research on gut microflora and the microbiome in humans and animals to uncover alternatives to AMU and how to promote health and prevent disease. Developing precise measures for surveillance on AMU (e.g., how much antimicrobials are used versus discarded) to trace transmission of antimicrobial residues in different settings (e.g., food, environment). Identifying indicators and metrics to assess and determine what places in the system are effective targets for intervention. Capturing data via surveillance on AMR-related deaths for recording on death certificates to make the impacts of AMR tangible. Developing rapid diagnostics that distinguish between microorganisms to inform AMU treatment recommendations for humans, animals, and plants. Developing block chain technology to improve traceability of the processes involved from farm to fork and detect infectious outbreaks. Learning how to develop cost-effective behaviour change interventions that are tailored to high-risk populations and settings.	Involved in up to 32,766 feedback loops, with a given feedback loop containing between 2 and 30 other CLD factors, indicating that the CLD factor ‘research, surveillance, development, and innovation’ heavily impacts and is heavily impacted by many parts of the system.
**Collaboration (overarching factor)**	Increasing collaboration by: Developing information networks between nations, governments, and industries to share benchmarking data to determine how AMR initiatives (e.g., National Action Plans for AMR) impact different sectors and to foster adaptive learning. Building trust between human and particularly agricultural/animal sectors to motivate sharing of information about AMU, AMR, and actions without fear of negative economic consequences.	Not applicable because the overarching factor ‘collaboration’ impacts the system of factors in the CLD.
**Leadership (overarching factor)**	Changing the intent (i.e., values and goals) that drive the system by leadership (from individuals to formal leadership bodies): Creating the vision and goals of how we want the world to be and what values we want to uphold (i.e., economic profits or health and wellbeing as our indicator of progress) and actions needed to achieve and maintain success. Implementing the Sustainable Development Goals, and determining the infrastructure needs and actions necessary to work through conflicting agendas across nations.	Not applicable because the overarching factor ‘leadership’ impacts the system of factors in the CLD.

### Feedback loops

The final CLD contained several feedback loops, which occur when an outcome of some causal process is also an input and provide a measure of the complexity of a system. Of the 91 factors in the CLD, the majority were involved in up to 30,000 feedback loops or more, with a given feedback loop involving up to 33 other CLD factors. The remaining CLD factors were either not part of any feedbacks: (n = 5: ‘national budgets, money, and funding’; ‘movement of people’; ‘psychological health’; ‘existing health care infrastructure’; and ‘host microbiome’); one feedback (n = 2: ‘understanding and awareness of science, evidence, surveillance, and best practices’; and ‘science and academia’); or two feedbacks (n = 1: ‘new and emerging foods’).

Eight of the 14 CLD factors identified as leverage points involved up to 30,000 feedbacks or more, one CLD factor was part of one feedback, and two CLD factors and the three overarching factors identified as leverage points were not part of any feedback loops, the latter because participants indicated they impact the entire system (Table 2).

## Discussion

This study aimed to identify the complex system of factors influencing AMR relevant to the European food system and leverage points for intervention to address AMR. Two CLDs were developed by different sets of participants across our two workshop sessions. While workshop day one uniquely identified three CLD factors and several new connections between CLD factors and workshop day two uniquely identified ten CLD factors and many new connections, the models were combined into one participant validated CLD because the majority of factors overlapped. Despite the many similarities, the richness of the data differed between the two days. For instance, workshop day two participants, who were more likely to represent perspectives that are less traditionally engaged in AMR discussions, tended to provide greater sector-specific and contextual information about issues than workshop day one participants who were more likely to have expertise and experience in AMR. Workshop day two participants also identified and discussed in greater detail social factors such as trust in leadership and inequities, and issues relevant to CLD factors such as ‘non-antimicrobial infection prevention and control by the public (e.g., hand hygiene, social isolation and taking sick days)’ and ‘non-antimicrobial infection prevention and control in other social settings (e.g., workplaces)’. Thus, by engaging different sectors, we were able to identify a broader range of factors and deeper understanding of how actions in particular sectors may influence AMR.

Many of the factors identified across both workshop days align with factors previously described in the literature. AMU for human health care, for animal food production and AMR spread were identified as key drivers of AMR and re-emphasized a strong need to better understand the factors driving AMU, and AMR spread in the environment [21–23], to find ways to incentivize the pharmaceutical industry to invest in developing new antibiotics [24], and to explore the microbiota as one way to identify alternatives to antimicrobials that may improve immunity and fight infections in humans [25]; and produce healthy food animals [26, 27]. Other participant-identified factors which are also described in the literature as impacting AMR included personal AMU practices [23, 28, 29], consumer food demands [30], patient demands for antibiotics, patient-doctor interactions, prescriber knowledge, attitudes and perceptions, prescriber and dispenser training [28, 31], a need for prevention [32], and addressing populations and areas experiencing social inequities [33–35]. Five additional overarching factors impacting the entire AMR system were identified, reinforcing the potential impact that leadership/governance [35, 36], regulations [4], and climate change [37] can exert on the AMR system, and identifying the media, and collaboration across governments and industries as key factors to consider when reducing AMR. Our study combines the participant-identified factors in one CLD, illustrating how they fit together and interact, thereby deepening our understanding of how AMR is generated in the European food system. Model complexity demonstrates that AMR is the product of actions taken in different, often siloed, parts of the European food system and between European countries, and how AMR exerts health, social and economic impacts across the One Health spectrum. Continued efforts are needed to engage stakeholders not usually involved in AMR discussions to build commitment and identify actions that can sustainably tackle the AMR challenge [20, 38]. Because AMR implicates human, agricultural, and environment sectors and participants described systemic issues such as social inequities as underpinning AMR, findings suggest that a whole of government approach to tackling AMR is also necessary.

Our study identified three shallow and eleven deep leverage points for AMR interventions per Meadows [16]. Shallow leverage points reflect less transformative actions that decision-makers often target: ‘retailer demand for product’ (e.g., through new procurement rules); ‘national budgets, money and funding’ such as directing funds to research, development and innovation (e.g., investing in the development of new antimicrobials) and to increasing investments in health promotion and prevention; and ‘new agreements, standards and regulations’ (e.g., setting consistent AMU standards across nations). Of the eleven deep leverage points, nine targeted changes in the design of the system by implementing new feedback loops that deliver information where it is missing about the consequences of choices that could cause people to behave differently [16]: ‘consumer choice, demand and behaviour’; ‘research, surveillance, development and innovation’; ‘understanding and awareness of science, evidence, surveillance, and best practices’ among different audiences and sectors; encouraging ’non-antimicrobial infection and disease prevention and control measures’ (e.g., on-farm, by the public); encouraging ‘psychological health’; and fostering ‘collaboration’ between governments and industry across nations to share information and intervention impacts and engage in adaptive learning relating to AMR. The covid-19 pandemic, which occurred post-workshops, has illuminated difficulties in achieving a coordinated mitigation approach and offers important lessons on how countries can improve collaborative efforts to address health emergencies like AMR. The deepest leverage point was ‘leadership’ because it targeted changes in the intent that drives how the system operates [16], with a focus on leadership determining a new goal for the system (i.e., health and well-being for all and achievement of the Sustainable Development Goals, as opposed to business viability of current agricultural practices), and then changing the underlying structures of the system (e.g., infrastructure, monitoring systems, funding) needed to achieve and sustain the goal. While participants called for comprehensive multi-faceted interventions that act at the identified leverage points, the intent of the system is the most challenging to change, requires diverse sectors to work together to examine the ultimate causes of unsustainability that impacts AMR, and is likely most needed to achieve a transformation in the system that sustainably tackles AMR [39].

Understanding whether the above leverage points are ideal targets for sustainable change requires assessing feedback loops. For example, the leverage point ‘national budgets, money and funding’ had zero feedbacks based on participant input, suggesting that changing national budgets will directly impact investments in things like health care and research, surveillance, development and innovation, which will themselves have impacts (e.g., food production, health system). The absence of a feedback mechanism suggests that this action has less potential to create unpredictable effects in the system. In contrast, eight of the 14 leverage points participants described were part of thousands of feedbacks, connecting to up to 33 other CLD factors, such as ‘consumer choice, demand and behaviour’ which has potential to change the system in ways that promote or constrain AMU depending on public demand but makes long-term impacts on other parts of the system difficult to anticipate or to control. This high degree of connectivity underscores participants’ perceptions of the complexity of the AMR problem and the challenge with finding long-term solutions, and additional research is needed to determine how effective these leverage points are in potentially disrupting or slowing down vicious feedback mechanisms or amplifying virtuous cycles within the context of these highly networked feedbacks. This underlines the need for the development of a learning system that can capture the most important feedbacks in a specific context [40].

A strength of our study was the use of a participatory and systems thinking approach to integrate a breadth of perspectives from stakeholders representing different sectors and disciplines, including those less traditionally engaged in AMR discussions, but whose domains of expertise directly or indirectly impact AMR and who are thus essential to addressing the problem. Our approach enabled participants to share, ask questions about, and build on each other’s knowledge in ways that were respectful of different views and knowledge and led to the development of a complex CLD. Thus, our approach demonstrates that bringing together participants from different sectors, with different priorities, and varying levels of AMR expertise is a successful way to generate new, shared understandings about the complexities of AMR, and where and how to potentially intervene, reinforcing these methods as useful to build capacity for systems thinking and systems-informed decision-making [41]. Moreover, our approach yielded a tool (the CLD) that may help governments, industries, the health sector, advocates and the public visualize the complexity of AMR and their roles, and that can be used to explore how interventions might impact AMR and other parts of the system.

A limitation is that while the CLD was developed with a European view, workshop discussions had a European Union and Sweden focus as participants came from these areas. While participants did discuss factors impacting Europe as a whole, it is possible that factors unique to other parts of Europe may not have been captured. We successfully engaged diverse perspectives from human, animal and crop systems and different organizational types at subnational, national and regional levels, however, inclusion of other sectors such as travel industry and the environment sector may have shed insights on additional factors not captured in this study.

While we purposefully engaged a wide range of experts in different content areas with varying knowledge of the technicalities about AMR, we did not independently verify their statements and did not determine the relative importance of the identified factors which can be explored in future research.

## Conclusion

By engaging diverse perspectives, we created an interdisciplinary CLD of factors influencing AMR relevant to the European food system that spanned the One Health spectrum and numerous potential connections between these factors and potential feedback mechanisms. This demonstrates the complexity of the AMR problem and challenges with finding long-term solutions. The identification of factors and feedbacks was useful to find relevant leverage points or places in the system to target interventions. Leverage points that target regulations, such as government’s setting AMU limits, or investing national budgets in prevention or research may be easier to implement. These actions in turn can support broader comprehensive cross-sector multi-pronged actions to redefine the vision, values, and goals of the system and sustainably transform AMU and tackle AMR. This provides the foundation for building more resilient societies in the face of growing AMR.

## Supporting information

S1 Table Quotes per theme. (PDF)

S2 Table Quotes per overarching factor. (PDF)

S3 Table Quotes per leverage point. (PDF)

S4 Table 91 CLD factors by 8 categories with definitions. (PDF)

S1 Fig Methodological approach. (PDF)

S2 Fig Day 1 workshop facilitator-revised CLD. (JPG)

S3 Fig Day 2 workshop facilitator-revised CLD. (JPG)

S4 Fig CLD of AMR in the European food system. (JPG)
