# Supplementary material for: Factors influencing antimicrobial resistance in the European food system and potential leverage points for intervention: A participatory, One Health study
Source: PLoS One. 2022 Feb 22;17(2):e0263914. doi: 10.1371/journal.pone.0263914 (PMC8863257; doi:10.1371/journal.pone.0263914)
Supplement: S1 Table — (PDF) [file pone.0263914.s001.pdf]

S1: Quotes per theme

Please note:
Quotes per row often contain the conversation of multiple participants. In keeping with the University of Waterloo Ethics requirements, we have labelled each contribution per participant as "P" and for facilitators as "F".

**THEME: AMU and AMR Spread**	
**Sub-theme/Sub-Topic**	**RELEVANT DE-IDENTIFIED WORKSHOP QUOTES**
AMR in environment	Workshop day 1:<br><br>P… the environmental influence, because all the waste goes into the environment. So the resistance builds up in the environment, and the big question, it relates to the transmission then from the environment back to either the human population or the animal population.<br><br>P: Yea. There is an intermediate step there as well, which I don't think is on here which is wastewater treatment.<br><br>P: Yes.<br><br>P: Waste water<br><br>P: Yea, I think that will most warrant its own spot, wastewater treatment. It is so critical to this.<br><br>F: And I think your point [participant] your point was we have got resistance into the water environment. I think what you are talking about is antimicrobials entering them, the water environment, and then selecting for resistance within the environment?<br><br>P: Yes the resistance develops naturally in the environment due to the waste.<br><br>F: So for those I am just going to put AMs in environment, and probably that is coming somewhat from human use.

	P: Yea. Both human and animal use. And It comes back to the environment into the human population and the animal, the resistant pathogens.<br><br>F: And then that is going to resistance then into the wider environment.<br><br>P: It is not only the antimicrobials themselves, it is also the resistant bacteria and then again it is coming from human and farm effluence.<br><br>P: And the whole recycling of water, and especially now in Europe, in Southern Europe it is a big thing because we are talking about massive use of recycling water into agriculture and ….crop production.<br><br>P: This is also big.<br><br>F: So we do this is entering the water environment and then some of that water then being used for crop production.<br><br>P: Crop irrigation.<br><br>F: Okay. Were there any other points that people wanted to add before we move onto the next kind of thing?<br><br>P: Yea not entirely wastewater treatment.<br><br>P: It is coming. It is definitely identified as a major hot spot for the selection of resistant organisms definitely.<br><br>F: And did that then go somewhere? Where did that go back to? I guess … so this is entering the water environment and then waste water treatment facilities.<br><br>P: Yea. That would go back into the environment.<br><br>F: Environment. It circles through.

	P: But wastewater treatment varies enormously from different areas even within say a country. It will be different ways of dealing with wastewater.<br><br>F: Okay and then I don't know either [name of participant] or [name of participant] you mentioned manure and the –<br><br>P: The crop production.<br><br>F: The crop production but also the run off from farms and other …<br><br>P: Also from the wastewater treatment, goes for irrigation.<br><br>F: there was something else you said…<br><br>P: It was not only … contamination with the antimicrobials, It is also with the resistance and genes
	Workshop day 2:<br><br>P: As I already told you I think the environment dimension of the manufacturing release and patient excretion of antibiotic stuff with this coming out? So that environmental…<br><br>F: And I think that is something that is not here. So we have resistance entering the wider environment, but we don't have any antimicrobials. Like the actual selective pressure.<br><br>P: That is right.<br><br>F: So I think we need to add antimicrobials in the environment.<br><br>P: And as I said, both from our own manufacturing operations, but also then from patient excretion.<br><br>F: And so that is coming from human use.<br><br>P: Yea, and production of antimicrobials for the human use.

	F: Yea, but would that one wouldn't be different between humans and animals, right? The production antimicrobials?<br><br>P: No. No. Pretty much the same kind of thing.
	Workshop day 2:<br><br>P: one thing I would underline is that the connection between human use, terrestrial farming use and then resistance, it gets lost on the way somehow here, but also I think [P's name] mentioned it before, that it should be more clear that the human P:…antimicrobial use and terrestrial antimicrobial use does not only put out resistance, resistant bacteria, but it puts out a lot of antimicrobial residues in the environment. So that one should be clear from both ways, because then in the environment, the new resistance is produced by environmental procedures that is, are ongoing outside of the human reach, so to say.<br><br>F: And that would probably be the same then. There is human use. There is terrestrial use. There is companion animal use and agriculture use and all of those would be then contributing to antimicrobials in the environment?<br><br>P: Yea, yea.<br><br>F: Okay. So we see here, resistance entering the wider environment and waterways and it goes to resistance in food products and it goes to people for sure. I am just wondering if there are any other links that it might go to. We don't have any direct link to people, but that is implicit there.<br><br>P: Alright, because I think the antimicrobials entering the environment are then selected for resistant bacteria in the environment.<br><br>P: In the environment. Yes.<br><br>F: Is there another pathway coming out of that? As we have more antimicrobials in the environment, is it linking to anything else?

	P: Yea, that comes back to human health for example. That comes back to humans. If you have resistance in the environment, that exchanges, there is an exchange between the environment and humans and, there could be a pathway there I think.<br><br>P: You have something at the resistance, the unknown food exposure for instance.<br><br>P: Yea possibly.<br><br>F: The non-food exposures here, and then there.<br><br>P: From the environment through onto.
AMR spread between non-food species and animals, between humans, and between humans and animals	Workshop day 1:<br><br>P: Yea, I thought non-food species may be, may need to be put on there.<br><br>F: So is it other species?<br><br>P: Well yea so in agriculture context, there are obviously tons of farms in Europe, and there must be other systems where you might actually get quite high levels of use that is fairly unregulated I would imagine because it is not entering the food system, but maybe big drivers of resistance,…[such as] farming of cleaner fish. So these are fish that [are] now farm[ed] to live with salmon to eat the sea lice on the salmon to control sea lice problem. So there, we use antibiotics in the cleaner fish, but these are not necessarily declared as being used in the salmon sector, because you know …<br><br>P: Because we don't use any antibiotics in salmon.<br><br>P: We are not. We are not using many antibiotics. We are using them in the cleaner fish, but I can see that there may be some non-food examples there that warrant inclusion, that might complicated things, but actually may be sources of resistance… That is a a specific example, but non-food animals, that aren't companion animals, that aren't wildlife, I would …put it on there.... Ornamentals or, yea they are imported a lot, ornamentals as well. You can use any, I mean that is the other point, right, you can use any antibiotics in ornamental animal species …you can use any antibiotic you like.
	Workshop day 1

	P: So we talked during coffee about increased connectedness globally, and speed, so and now we have exposure to resistance in imported products. What about the increased connectivity among people globally. The concentration of people, urbanization. I mean all those kind of very large factors.<br>P: The connectivity.<br><br>P: Exposure.<br><br>F: In some places between people and animals?<br><br>P: Yea, both..<br><br>P: And products, connectivity.<br><br>P: I am not sure that that importation of the products is quite such a risk for EU in terms of you know the quite strict importation, guideline systems in place… higher, higher (on the model). So yea that is it. Yes. I mean I think our food safety systems are pretty robust for imported products in terms of, they have to be produced, you know to align with EU standards. So I think that is probably quite a low risk on that diagram. I know we have already touched on that.<br><br>P: But it comes at a cost.<br><br>P: I disagree with that, I don't think there are any regulations that protect us from AMR issues. There is nothing there. There is a lot of things about ….<br><br>P: Residues there is.<br><br>P: Residues is the only thing, but it is a totally different thing. We don't have any antimicrobial resistant bacteria … We don't have surveillance systems. We don't know. We don't test for any product. That is a big thing in this …development because even labelling countries, whether they have kind of beginner to advanced surveillance systems it is a big thing for them, because they don't want a label attached, because this may have trade implications. So imagine if they are testing out the products, this may totally… so I think we have information about what is coming into Europe in terms of load of resistance and [inaudible ? [there are other] many things] and we just don't know.
	Workshop day 1

	P: Perhaps there is one thing that comes to mind and now that you mention, this post processing is perhaps a big topic but also in Europe we don't use, for example, [inaudible] examination. We rely on our prevention of food borne pathogens of primary levels. So we try to, it is not possible to control in farms. But you know the places like in the US, they have more harvests, washes with chloride and with … acid so that is a lot. So …..because perhaps it is not that simple to control that primary level, resistance, unless you have a very stringent policy in use.<br><br>P: Well you know the places that you can supply different resistance treatment, have an affect also on the risk of contact with the resistance bacteria in your meat.<br><br>P: So that feeds into some of the discussions around AMR in pork and things like that with … and abattoir and the food<br><br>P: AMR is not on, the transmission is not via food, and it is more via contact with …
	Workshop day 1:<br><br>P: One of the things that we haven't mentioned …The movement of patients across Europe either as a result of an incident or though health tourism, we know from outbreaks that we have had and still have ongoing, that we are moving patients colonized with nonresistant organisms between countries and health facilities that they aren't going on to cause outbreaks. I think that is something that needs to be, um, considered, because that can have a massive impact in a, in a health care facility.<br><br>F: I am wondering…we have got the migration, like the movement of workers, but we also have like the movement of patients and there is probably…within country movement, and there is global movement?<br><br>P: Yea, yea.<br><br>F: Okay. And that goes to the international piece right, that health tourism as well.<br><br>P: Well certainly within Europe and outside Europe.<br><br>P: There are big differences in the levels of AMR across Europe, right, in all this. So movement within Europe will be …

	P: Yea, I mean even within Europe we have documented outbreak.

<table>
<tr><th colspan="2">THEME: Economics and Agricultural/Aquaculture Production Practices</th></tr>
<tr><th>Sub-Theme//Sub-Topic</th><th>RELEVANT DE-IDENTIFIED WORKSHOP QUOTES</th></tr>
<tr><td rowspan="2">AMU in food production</td><td>Workshop day 1:

P: There is a whole conversation to be had about whether we are using antibiotics optimally. Like, do we have appropriate diagnosis to distinguish between a viral or bacterial infection. Why do we treat for once a day for 7 days? Well it’s because there’s 7 days in a week and it works (laughter). But there’s some work being done that shows that’s not the best way. It doesn’t give you the best health outcomes. And it has implications for overuse so there is debate at the moment about…well, there’s one school of thought that says well actually when you do feel bad, stop taking the antibiotic. That’s a different school of thought but that stops you taking antibiotics that you might not need, so that could fall into… we can optimize our use for humans but for animals…should metaphylaxis of 10,000 animals [be] the right way to do it or should we be isolating the animals and treating them individually? So we’re not using massive amounts of antibiotics we don’t need. It’s the same in the fish farming, you have to treat the pet.

P: just on the issue that [name of participant] mentioned, [that]…issue …it is a cost-benefit one. It is basically an economic issue. It’s cheaper to treat the whole pen through the feed or the water than to isolate and treat individual animals. It’s an economic issue.</td></tr>
<tr><td>Workshop day 2:

P: Yea for the focus that of course is the driving force for using antibiotics [for] all kind of purposes is [for the] low…prices…</td></tr>
<tr><td>Good Farm Practices and AMU, AMR and costs</td><td>Workshop day 2:

P: you have good farm hygiene practices…

P: The… the way we reason in [name of European country] is healthy animals don’t need antibiotics, and healthy animals we get that, not only by good hygienic standards, but also, but good living conditions for animals. I mean, good stables, spacious stables, etc.

F: So….[call it]…good farming practice?

P: Yes.</td></tr>
</table>

	F: And that includes management, hygiene,<br><br>P: Not only hygiene, but also nutrition.
	Workshop day 2:<br>P: One thing that I am missing from this map is like, kind of like infrastructure approach to farming practices, because then you talk about like different resources that is needed to, you know, grow plants, or like, you know, animal husbandry, because now they are very much talking about like different kind of like new movements about authorization, about the approaches of our economy for example that we should use more of our resources instead of like looking at them as a sort of waste and then you have some factor about use of like sewage water and brown water and lots of those, which is becoming more and more main stream because I mean if we are losing all the nutrition in our brown water, then you know be ultimately seen as a source of nutrition. What kind of like technological transition can we have in order to use them as a resource, instead of seeing them as waste and runoff?<br><br>F: So then if that's, that is stuff coming the farm, some of those waste pieces, how can we link this back into …?<br><br>P: I mean use them back. I mean closing the loop and also like take a look at this societal infrastructure like all of the brown water from the human could be used as a source of nutrition, but regulation wouldn't let them because there is a risk of heavy metals and you know, but there are many different initiatives trying to just extract phosphorus, nitrogen, lots of those and using them as fertilizer back in farms, which is based on concept of similar economy. So, this is like a technological infrastructural transition that could actually help us with better sources that house better farming practices as well.<br><br>F: Yea, cus I, and I think, I was just trying to capture sort of using this… like if we think about sort of the wastewater, can [we] then connect it to maybe [to]…cropping and some of the feed?<br><br>P: Right.<br><br>F: And then that would have a direct, as nutrition and what we feed our animals affects whether their health not directly, their illness?<br><br>P: Yea
	Workshop day 2:

	P: what I miss is something that you talked about, prevention [inaudible] if we have healthy people, healthy animals, you diminish the demand… hopefully I would say, and I don't think you have it clear enough really that…that you must focus on health. What you talk about infection, prevention, etc. etc. etc. Then of course you have put a focus or something that it is our problem, otherwise, and retain of [inaudible] any kind of animal products in that time, that has of course focused on low prices, high efficiency, and it is not an easy way to solve the problem you talked about. So that of course is, oh, very important link to discuss the price of food.<br><br>P: And animal products and of course sitting in the… engage in animal welfare, I think there must be a very focus on the welfare, because you have to, if you have to, prevention is so important.
	Workshop Day 1:<br><br>P: And if you think of optimal use in animal farming, then I think that's a tricky terminology because it, it starts out – or it sets out from the system you have now, and not questioning the system as such. And that is not captured by good farming hygiene practice, but it should be captured by good farm practices. Like, we might have to change how we raise the animals, how they are together - I mean more structural changes to avoid stress.<br><br>Another participant: Yea.<br><br>P: I mean, reducing density- So, it's more than just good hygiene practices, it is good farming practices.<br><br>P: And it's more than [animal] welfare as well because I just added on 'stress' there, and really that just captures exactly what you just said there – which is good farming practices.<br><br>P: there needs to be a fat arrow to costs.<br><br>P: That's right. Definitely. That's very important.
	Workshop day 1:<br><br>P: I think more from a farming perspective, where you, you would improve your farming practices, and therefore in the short term there would be a large investment, but in the long term as you are reducing your disease burden.

	P: If you are talking about restructuring the production facility building wise. Yes I agree.<br><br>P: The studies that are complete …. in the [name of some Northern European countries], are on exactly that.<br><br>P: Yea.<br><br>P: The costs are upfront and immediate, where the benefits are more long term.<br><br>P: Yea sure.<br><br>P: The question is how to you get into big costs upfront given that you are working with a low margin industry.<br><br>P: Yea.<br><br>P: But then it would be spread out over a longer period of time.
	Workshop day 1:<br><br>P: But it also affects if then if consumers are willing to pay a higher price for the final product, then the producer can pay all the extra costs of putting these extra quarantine measures if you like on the farm – the biosecurity measures on the farm – which would push up the price of livestock products. So, I think it's all along the food chain (P agreed), because the problem at the moment is that livestock producers have a very, very light margin if any in their production and anything that might change that margin puts them into the red so that's where this is a really tight economic question. But in the future, you're right, if consumers are willing once they become more aware and understand the consequences of AMR and so on, to pay a little more for their final food then the producers will be able to build that into their system because it folds back along all along the chain.<br><br>F: so we have this producer profitability which I think you are highlighting as being a very narrow margin –<br><br>Some Ps: yes

	F: and you have retail costs – we have meat and eggs here but maybe it is just food generally –<br><br>Many Ps: Yes, and that then is linking to “this” – so what we are willing to pay for food is going to affect producer profitability.
	Workshop Day 1:<br><br>P: But it also affects if then if consumers are willing to pay a higher price for the final product, then the producer can pay all the extra costs of putting these extra quarantine measures if you like on the farm – the biosecurity measures on the farm – which would push up the price of livestock products. So, I think it’s all along the food chain.

THEME: Consumer Demand	
**Sub-Theme//Sub-Topic**	**RELEVANT DE-IDENTIFIED WORKSHOP QUOTES**
Consumer demand, willingness to pay more for food and AMU in food production	Workshop Day 1:<br>P: So it is coming, but a lot more customers…I mean increasing this transparency, it is top down things, consumer requiring more transparency and some of you mentioned pork antibiotic-free pork in France. I think the real driver is …by consumers.
	Workshop Day 1:<br><br>P: But it also affects if then if consumers are willing to pay a higher price for the final product, then the producer can pay all the extra costs of putting these extra quarantine measures if you like on the farm – the biosecurity measures on the farm – which would push up the price of livestock products. So, I think it's all along the food chain (P agreed), because the problem at the moment is that livestock producers have a very, very light margin if any in their production and anything that might change that margin puts them into the red so that's where this is a really tight economic question. But in the future, you're right, if consumers are willing once they become more aware and understand the consequences of AMR and so on, to pay a little more for their final food then the producers will be able to build that into their system because it folds back along all along the chain.<br><br>F: so we have this producer profitability which I think you are highlighting as being a very narrow margin –<br><br>Some Ps: yes<br><br>F: and you have retail costs – we have meat and eggs here but maybe it is just food generally –<br><br>Many Ps: Yes, and that then is linking to "this" – so what we are willing to pay for food is going to affect producer profitability.
	Workshop Day 1:<br><br>P: But it also affects if then if consumers are willing to pay a higher price for the final product, then the producer can pay all the extra costs of putting these extra quarantine measures if you like on the farm – the biosecurity measures on the farm – which would push up the price of livestock products. So, I think it's all along the food chain
Health status and food preferences/demand	Workshop Day 1:

	P: I think there is a need for more like a social lens to this as well. Like, if you identify the main drivers – that would be "demand for product" if you relate that to "food production". And then for "human use" it is may be "vulnerability" or "burden of illness" – I don't know which of the two is the main driver, but you have 2 main drivers for the whole system, and then if you look at the demand for products, they have issues that relate to consumer preferences and how they are being influenced by occurrence by diseases and resistance and how they will change their behaviour from these kind of influences. I think there are a lot of things that need to be teased out as it relates to human choices as you mentioned.
	Workshop Day 1:<br><br>F: [name of participant] mentioned there are a multitude of factors that affect what we choose at the grocery store. Is it worth listing some of those to see if we can put them down on here?<br><br>P: Well, if they switch it will have implications for many parts of the system. So yup, probably [need] a new [map/causal loop diagram].
Religious or cultural food preferences/demand	Workshop Day 2:<br><br>P: I have some suggestions…that it is important for, it is the culture preferences… On food preferences, if you are vegan or vegetarian or you eat Kosher or Halal food, and… this is something that you can connect to population demand for product.
Increased interest in sustainability and food Preferences/demand	Workshop Day 2:<br><br>P: …I think is really specific about the Swedish system is that the consumers and also the retailers and the food producers has a very high awareness. They are not 100% you know, I mean they don't have 100% knowledge about everything, but I think that there is an awareness which might make it easier to find solutions. I don't know if that fits, but at least that is my point.<br><br>F: Does that, is it awareness of each other or is it awareness of resistance?<br><br>P: Awareness regarding more like sustainability as a whole, and like an openness towards actually being part of the solution.<br><br>F: So that awareness then would contribute to the transparency. Would it? in between those different groups?

	P: Yea. I think it would definitely affect the demand. I mean both demand from the population but also I don't want to say that, it is not only consumer driven. It has been done. It is also something that is in the trade business, although the consumer is going to push them.
Consumer understanding and transparency in how food is produced	Workshop Day 2:<br><br>P: the food industry doesn't work like, really like you know connecting farmers to people. Like the food is grown here. Here is your food. It is about like a huge global logistic system that is more or less like the retailer asks, I need 50 lbs. of avocadoes first class on this day and I don't give a dam, if they come from, Ecuador or [inaudible]. I want that. I mean it is most of the time something like that, and of course like many different retailers try to take that responsibility on themselves, like to talk about where is the origin of food comes from and so on, but I mean, being at a supermarket, and buy an apple, could you tell me like how old the apple is? How much nutrition are you getting in that apple? When was that harvested? Which farm it comes from? I mean basically like some started to work on traceability in the food sector. I don't think it is working and then transparency towards the customer is almost zero. When you talk about like I mean like organic food coming from the greenhouses and the vegan people go for the organic option. Do they know that the nutrition in those come from the slaughter houses, a block from the market for example? You never talk about like issues like that. So, production methods, certification methods, origin of that logistic globalization and like all of this different things like the transparency issue in the food sector is really, really low.<br><br>F: So just to try to unpack that a little bit, so there's… because there is a lot behind what you are describing. So there are differences in production methods.<br><br>P: Sure.<br><br>F: I think what you are also characterizing is, maybe a knowledge gap between the consumer and the producers.<br><br>P: And transparency of course.<br><br>P: And certification is part of the solution, but method to secure something.<br><br>P: Certification, I mean you could talk about like organic labels…So I mean like how you try to like get some transparent information about the origin of food, what kind the certification, and what kind of like checklist

	and like requirements and so on and so forth, they failed, but how much of that information is shared with the consumers is one thing. Maybe it is not directly like the connected to your topic, but I mean seeing food as something as simple as the farmer produces and you eat it is a bit like, you know, naïve I would say.<br><br>F: So… so like some of the knowledge gaps, so there are some labelling pieces, like organic, or free from [antibiotics], or all of those things.<br><br>P: Sure.
	Workshop Day 1:<br><br>P: I think there has been some evidence on that on several European countries and I am thinking of France. When you go over to the store and you want to buy meat, you get the normal pork and you get antibiotic free pork from the stores as well and it would be interesting to see what kind of trend that will be in the sales of the antibiotic free pork, because it is a bit more expensive. I think maybe 20-25% than the normal mass-produced pork, but it would be interesting in maybe a year or two to look at the data on the sales figures of that and compare one with the other.<br><br>P: You do get unintended consequences from that as well, because you get people, you get spanners on welfare, because people say, how am I going to treat these animals?<br><br>P: Yea.<br><br>P: Because I am selling out and freeing him and I am going to lose a lot of money if I treat these animals. They will be fine.<br><br>F: Although if an animal is sick, it will not be entering a food chain, so there is a… there's a fine line there.<br><br>P: Yea.<br>P: Technically not. Technically.
	Workshop Day 1:<br>P: I am missing a link in the section about public demand for alternatives of antimicrobials. It is very much human centered, but I think there is a same demand and a lot of efforts for developing alternatives for food producing animals so I think we should somehow connect …

<table>
<tr><td rowspan="4">The quick fix, habits, and demand for antibiotics</td><td>Workshop Day 1:

P: … it's related to habits. And of course not everyone is buying antibiotics to begin with but people pass them along the family to friends, and some people get them abroad when travelling because it's easier than in the country that they live in. So it's the whole mobility aspect as well</td></tr>
<tr><td>Workshop Day 2:

P: there are a lot of things you could of course ask when it comes to humans on why do we want to quick fixes with antibiotics. We very rarely applying a lot of the preventative measures we know we could, regardless whether that is changing our role, our behaviours, strong vaccination and stuff like that, and vaccination in one place. I actually believe that relates to, to like foreign practices and stuff like that.</td></tr>
<tr><td>Workshop Day 2:

P: …And, so, and then there is a lot of stuff, but coming back to the human side, we also need to somehow address like the… the human behaviours… and I always tell a story about my [child], once again, and after [the child] had…had some infection, [and] screamed for three days. I didn't sleep for three days, and then I went to the doctor with [my child] and although I understand that we should not always put in broad spectrum antibiotics, but I was, I was so tired, I was so… just wanted the worst, strongest medicine, because I wanted to sleep! When [name of child] is crying, I do not get any sleep, and then we wanted that quick fix.

[Many Ps: um hm]

P: People want that quick fix, so when it comes to the animals of course, we can somehow, I guess we decide somehow, but when it comes to the human side, we need to understand also the enormous pressure physicians comes under when, when a very, very angry [parent] comes in and screams and wants the ciprofloxacin or whatever for [their] kids.

F: So could we capture that as like patient demand?

P: Yea.</td></tr>
<tr><td>Workshop Day 2:</td></tr>
</table>

	P: I have to say that you include the companion animals. Because I think many times you forget that. When I, when we argued about the responsibility, I also argue then pet owners, because you have the responsibility to the same as you do. My dog wants antibiotics, etc. etc.,
	Workshop Day 2:<br><br>P: we are also more prone to go to the doctor immediately in these cases, which is a problematic thing, we are really healthier. We have a much, much larger ability to cure a lot of these minor illnesses by ourselves, but at the same time we, we demand more health care.<br><br>F: Is that, is that in part, because like coming back to the stress level - So you are stressed and you get sick, but then you don't want to take the three days at home and not work, and so you are looking then for …?<br><br>P: That is another very important reason for wanting the quick fix. Yea. Sure.<br><br>P: Yes. Our society, I mean our requirements for productivity at every, every day, every second. Yea.
Previous Experiences and demand for antibiotics	Workshop Day 2:<br><br>P: And both the culture preferences back to [name of participant] about the patient demands. The culture thing is that, in health care in [name of European country] one thing is, it's an issue in the health care system is that the immigrant families, they can go with their small children for a very mild cold. They go to the emergency room, and the thing is that that mild cold in their own country can kill that child. That is why [name of participant] said waited three days [before taking the child to the doctor], but they [immigrant families] don't want to wait one day, one hour. They rush to the emergency room, and they want antibiotics for the first, from the beginning, and this is, this is because of the experience that they have had in their own country. So this is another thing about the patient demand.
	Workshop Day 2:<br><br>P: Can I add something to that, because I had actually had like one personal experience that happened, because I mean I have parents from [name of country outside of Europe] and I grew up in the [name of country outside of Europe] and in both those countries, I mean you get antibiotics quite easily and the thing is I got like an ear infection a couple of years ago…and then my doctor [in Europe], he was like when I asked for antibiotics, he is like, 'oh, antibiotics!' Like I asked for poison. For me it was like 'yea'.<br>I actually like quite got a lot of antibiotics [as a] child…I wasn't thinking, but it is like different kind of like views on how dangerous antibiotics can actually be.

	And then you have this like resistant to yourself towards those. So they started like prescribing like mild antibiotics under two years to me, that the infection got so bad, that it couldn't be curable until they almost killed me giving me antibiotics after like two years. So I couldn't like even move like for two weeks and I was done with the dosage of the antibiotics after I used that, because it actually got [me] like more and more and more resistant… So it was like one thing that is like maybe like the culture difference, like how much resistance you yourself have towards…to taking, because you mean, maybe like your…your body or like the kind of like the [microbiota] in your body are more resistant than the other people because of where you come from or how high your resistance is.<br><br>F: And your previous exposure?<br><br>P: Yea, exactly, and then one more point that is maybe also, if you could capture that.<br><br>F: I think it is just…previous antimicrobial exposure?<br><br>P: Exactly.<br><br>F: And I think that then is going to affect, well it is not going to affect use, it is going to affect the success of that? So it is sort of the efficacy like whether or not the treatment works?<br><br>P: Right.

	Workshop Day 2:<br><br>P: Can I add something to that, because I had actually had like one personal experience that happened, because I mean I have parents from [name of country outside of Europe] and I grew up in the [name of country outside of Europe] and in both those countries, I mean you get antibiotics quite easily and the thing is I got like an ear infection a couple of years ago…and then my doctor [in Europe], he was like when I asked for antibiotics, he is like, 'oh, antibiotics!' Like I asked for poison. For me it was like 'yea'.

	I actually like quite got a lot of antibiotics [as a] child…I wasn't thinking, but it is like different kind of like views on how dangerous antibiotics can actually be.<br>And then you have this like resistant to yourself towards those. So they started like prescribing like mild antibiotics under two years to me, that the infection got so bad, that it couldn't be curable until they almost killed me giving me antibiotics after like two years. So I couldn't like even move like for two weeks and I was done with the dosage of the antibiotics after I used that, because it actually got [me] like more and more and more resistant… So it was like one thing that is like maybe like the culture difference, like how much resistance you yourself have towards…to taking, because you mean, maybe like your…your body or like the kind of like the [microbiota] in your body are more resistant than the other people because of where you come from or how high your resistance is.<br><br>F: And your previous exposure?<br><br>P: Yea, exactly, and then one more point that is maybe also, if you could capture that.<br><br>F: I think it is just…previous antimicrobial exposure?<br><br>P: Exactly.<br><br>F: And I think that then is going to affect, well it is not going to affect use, it is going to affect the success of that? So it is sort of the efficacy like whether or not the treatment works?<br><br>P: Right.

**THEME: Health and Social Care System**	
**Sub-Theme/Sub-Topic**	**RELEVANT DE-IDENTIFIED WORKSHOP QUOTES**
Antimicrobial stewardship	Workshop Day 1:<br><br>P: Stewardship. I think it needs to define stewardship because it means different things.<br><br>P: Yea….

	F: So that, you are going to bring in the infection, prevention, and control and the public health, like all of those pieces?<br><br>P: All sorts of bits. Yea.<br><br>F: Yea. Okay. So we are going to, can we agree that stewardship is sort of that broad sort of sense of what stewardship is. Is everyone okay with that?<br><br>Many Ps: Yea.
	Workshop Day 1:<br><br>P: it also … uh…the regulations, the presence or absence of infection prevention and control, all affects both the health care costs, the burden of illness and the use of antimicrobials, so you can make a whole bubble up there with all these elements on the human side similar to the veterinary side<br><br>P: And this could be in health care settings or in social care settings.<br><br>F: And then that would link in directly to health care costs?<br><br>P: Yea-<br><br>P: To the costs, to the burden of illness, and to antimicrobial use…in humans.
	We need to go away from always using broad spectrum antibiotics to actually much more narrow ones, but then we need to know what kind of infection we are trying to treat, and much more focus on prevention of infections, hygiene sanitation…
Norms and prescribing practices	Workshop Day 2:<br><br>P: Can I add something to that, because I had actually had like one personal experience that happened, because I mean I have parents from [name of country outside of Europe] and I grew up in the [name of country outside of Europe] and in both those countries, I mean you get antibiotics quite easily and the thing is I got like an ear infection a couple of years ago…and then my doctor here], he was like when I asked for antibiotics, he is like, 'oh, antibiotics!'. Like I asked for poison. For me it was like 'yea', I actually like quite got a lot of antibiotics for children, I wasn't thinking, but it is like different kind of like views on how dangerous antibiotics can actually be

	Workshop Day 2:<br><br>P: Regarding your comment about the first visit. There is a culture…in [name of European country] health care which is very difficult to understand for the people from other countries and the thing is that in [name of European country] the person is responsible for his or her health, but in many other countries, your, I mean, this is very usual, when going to a doctor here in [name of European country] in the health care and they say, what can I do for you? And then it is very… when ….immigrants [it was found] here in [name of European country] about their health, and their experience with health care, and this is the sometimes most of them mentioned that it is very strange, that it is not my responsibility. It is the doctor’s responsibility. Yea, you are laughing, because<br><br>P: I can see it is different.<br><br>P: It is very different. I mean… but, but it is difficult to understand and to find out that if I have diabetes, it is not my doctor’s responsibility. It is my responsibility to take care of my food, my physical activity and… and everything, but it is very difficult to understand and to accept it. That is why it is the culture.<br><br>P: Hm-mm. Yea.
Physician Fear and prescribing practices	Workshop Day 1:<br><br>P: Survival pressure through the media.<br><br>P: The trouble is certainly in the [area of Europe], we have this issue with sepsis that is driving the media, and is really making prescribers very nervous around antibiotics and prescribing, because the threat of a public case of sepsis and a child dies is seem to be greater than the greater risk in giving the antibiotic. So yea we have some conflict currently with other medical conditions.
Physician interactions with pharmaceutical industry and incentives and prescribing practices.	Workshop Day 1:<br><br>P: We had something about the selling of antibiotics, and profits derived from that and how that differs between countries.<br><br>P: It affects the use on both the human and the animal side of course.<br><br>F: So profit. Would that capture it? Profit for sale.

	P: I guess.<br><br>P: Yea, because there are different systems for how antibiotics are sold. Right. Even just to the consumer. Some countries in Europe, the doctors themselves will get you know financial profit for selling, but in other countries that is not the case.<br><br>P: Right.<br><br>P: Wherever you have regulation, the legal regulation of the use in animals and humans that then affects the profit possibilities, because it will be laid down by law who can sell.
	Workshop Day 2:<br><br>P: I don't know if this is something that has an aspect on this, but [name of country has] ethical rules on how we can interact and meet with physicians and you know getting access to them, and that is something that [P's name] also thought about. So you have to understand it is difficult for pharma companies to get access to health care professionals or physicians to talk about products, etc. and I was just thinking about you know informing perhaps or providing information to physicians about other types of alternatives, etc. because we are really talking about, if they prescribe one type of product that is more, gets more resistance than others. I don't know, because I don't know that much about AMR, but maybe that can mean something …regulations access to… access to health care professionals.<br><br>P: I just have a comment or a suggestion. What is your intention to increase the access of industry to the physicians? Was that the aim of your kind of proposal?<br><br>P: My comment?<br><br>P: Yea.<br><br>P: No, just you know I was saying that… as I understand [it], the farming industry [in name of European country] has a difficult time coming to the [acronym] as we call them, just to inform them about products, etc. so I think that I mean yea maybe that can be a hindrance to talking about other alternatives, etc. and getting the message out. So…

	P: And especially if our medical experts are insightful and to speak with professional decision, but we need to… stay very close to… to the approved texts from the agencies. We could never ever stop discussing any use. Of course that is a crime. So, these kind of ideas around what could be done instead of using [antimicrobials]…the only approved thing we have come from MPA is that you should use this and then we are allowed to… it is a crime to say anything else.
	Workshop Day 2:<br><br>P: I would really like to add… [the differences between] … [name of European country] and ...[region outside Europe] where there are no regulation …between the industry and the decision [makers], and one of the reasons why [there is] a very, comparably very rationale model of antibiotic therapy in [name of European country] is because we have such close, such strict regulation of how industry can work with decision makers. So that is one of the assets of the Swedish model actually and one should be very careful to disrupt that.<br><br>F: Is it the same on the veterinary side? Does anyone know? Are they allowed to market directly to the veterinarians?<br><br>P: I mean they can come and have product information evenings when they talk about products. Yea.<br><br>F: I am just curious if there is the same legal framework there for veterinarians.<br><br>… P: …It is also in…within the veterinarian side. It is so important.<br><br>F: Okay. So it is the same sort of rules and regulations in the conversation.<br><br>P: Yea.
	Workshop Day 2:<br><br>P: …We know we do not want to disrupt any of the ethical rules that we have today. What we just need to understand that when we talk about this, this consensus tripod culture here in [name of European country], and everyone talked and try to find good common solutions together. When it comes to this regulation, [it is there] for good reasons, absolutely…but it is also sometimes could of course limit the opportunities to find a common solution to something like this. There is good and bad always when not allowing communication.

	P: Oh, but I agree that the strictness has also some difficulty. It incurs some difficulties when we try to do these discussions on the national level and that is a problem, but… but we shouldn't mix the two I think. I mean we should not let the difficultness at the national level influence the strictness that we now have at the local level. We have the drug committees, which were established in [name of European country] a long time ago, to be the ones writing and the local recommendations to the physicians. While twenty or thirty years ago, the traditional treatment was mostly formed by the industry, and of course the industry has an interest in net gain and so that influences, influence here and influences still in many other countries that prescribers' pen, and that is not what is rational from a medical point of view. That is what is rational from an economic point of view and, and you have to keep this in mind.
Technological Changes and diagnosis and prescribing practices	Workshop Day 2:<br>P: …Generally in [name of European country]…before - nurses and doctors used their craft, the hand craft. They.., they exam…patients much more. Today…the reference is the computer system. You take a long [inaudible] report. You read and then you have the reference about you know this patient, and then we got into it [with the patient], and see, 'oh it is not as it was written, or [as] I was reading in the [report]'.<br><br>…Fifteen years back, [nurses and physicians] got into it and said oh this is the traffic accident. Okay…go in there first [to see the patient] so that can shake out what is the problem. What do I see now? How is the body? How is everything and …get connection to the patient and the relatives, and then…go and get a report… and that is a huge difference when [determining]…what is the problem with the patient? Because [one] can see a difference now, and I also asked a lot of nurses [I know] before I came here, [and they said they] see that we use more broad spectrum antibiotics directly [compared to before].<br><br>F: And do you, do you have a sense of what caused that shift…away from sort of that hands on thing.<br><br>P: Yea, I think about that, it…it's not so, it is not so…. You know it is better to be theoretical in your, in your studies or your analysis and you haven't seen a patient, but you have read a lot of papers, and you have written a lot of them, and we can talk about everything what you don't know how you can smell infection or you can't smell cancer, because if we have the hand craft, you can smell everything and you can say that is that and that. That is very important. Nowadays I can see that the nurses and the doctors who are … who are… educated now, in the last years they are not so skilled at, to diagnose things. They don't touch the patients. They don't smell them and look in their eyes…

	F: So it is an issue of how health professionals are trained. Like their education and training has shifted obviously to be more evidence based and more clinical, like more objective and evidence first, the patient relationship comes at some point after. Is it education and training of health professionals?<br><br>P: It is about the society. Everything you have made by hand. People who work with hands, they are not so good, and people who have academic and can make all, they are wonderful. We love them. [Group laughed slightly] So that is the problem in the home, because we don't, in Europe, this is very important to get back that… the education and the experiences. It is about how you examine patients, really can see what is there... I mean, I'm not meaning that nurses and doctors are not good to examine patients. I don't mean that. I mean that the system make the opposite that we are living at what has been written before, and it is a reference. So they don't use their intelligence as they have already, because we know a lot of people, when you have worked for many years, you can see, but so I don't mean that they don't have the skills. we just need to know, use them<br><br>F: use their tacit knowledge.
	Workshop Day 2:<br><br>P: I just wanted to build on that perhaps, you were saying about computer systems. I mean I think we all read in the papers how health care system is becoming, I mean and education system, etc. everything is much more administrative work nowadays, so maybe they lose some of that time with the patients and you know learning how to, let's just say see and smell the infection, etc. So I don't know, administrative work or more administration in the profession. I don't know.
	Workshop Day 2:<br><br>P: …before the doctors were more experience-based and now I mean it is like you know e-health and these needs are coming that you could actually like count on, like variable devices and robots in your blood, and in real life like kind of like you know monitoring system that actually help the physicians to have like more precise diagnosis.
	Workshop Day 1:<br><br>I mean that is a big drive in Europe now towards, moving towards that. E-prescription as being cross border. E-prescription is being introduced and these kinds of things, and they might indirectly influence the chart.

	P: So E-prescription is that also including the, now you can get examined on the Net, medicine without seeing a doctor. Is that included there?<br><br>P: Good question there.<br><br>P: Is that kind of trend.<br><br>P: Consultations.<br><br>P: Yea that is right.<br><br>P: It is all part of digital health I would say.<br><br>P: Yea digital health.<br><br>P: Does that exist for animals as well?<br><br>P: Face-timing (laughter)
	F: …I don't know about Europe. Right like you will have people take a photo of a calf, one of the cow. So that doesn't actually go out. You do have a relationship with veterinarians, but they will diagnose based on that?<br><br>P: It's the same.<br><br>P: So, is that good or bad or it is just a factor that will influence parts of use, right?<br><br>P: I think so. I mean you are making a diagnosis with perhaps not an imperfect sample, but maybe it is better than just going out without that consultation. Right?<br><br>P: But it is driven by cost. It is a cost issue.<br><br>P: it's an economic issue.<br><br>P: Obviously it is a resourcing as well. It is a resource issue.

Resources, capacity and stewardship	Workshop Day 1:<br><br>P: So I think it is also often discussed in the community and the primary health care is I mean, how specific….based on qualities, is the necessary staffing and public GPs… for example have less and less time to … consultations with the patients, so they have to diagnose based on, on perhaps optimal methods and they are [tight on time to provide] education [for] patients, so they found that difficult not to prescribe antimicrobials. So a lot of these really has massive I think influence on, on the level of use in community in general, and I think we are in the direction that this is not improving, it is getting worse. So if you have to treat the patient in, or diagnose somebody and prescribe something in five minutes, then you do something that perhaps is not optimal compared to having all the time to explain. And uh<br><br>P: It is the conflict between treating individual and then population.<br><br>P: I think that is probably common to most of the countries in Europe.<br><br>P: Yea.
	Workshop Day 1:<br><br>P: That is probably what we discussed before if the GP have enough time to conduct the necessary tests and they can inform a decision whether an antibiotic is needed or not, that risk would not be there, but of course if the poor guy has to decide in that minute, would I risk that this time suffers from my decision and then I get the [inaudible] as a result. I don't know, so I think it is all connected.
	Workshop Day 1:<br><br>F: Just before we turn that over, so the diagnostics, right you talked about the need for better diagnostics. Where do you see that fitting in here?<br><br>P: Somewhere in like the proper use of antibiotics is driven by that… we do not have either the cost for the diagnostic kit. It is found to be too high, or there is no time to wait for the results. So not doing the right diagnosis of course drives you know antimicrobial use.
	Workshop Day 2:<br><br>P: And it can be interesting to see the, I understand why [name of national institution] did not want to use some of that diagnostic tests, because they are, as I said, they could be quite expensive, and when the

	alternative is to just use a broad spectrum antibiotic, which costs nothing, they are almost free today, then where is the incentive…. Why spend money to do that right diagnosis, when the simple treatment is so inexpensive.
	Workshop Day 1:<br><br>P: – I mean, if you have the procedures in place, if you have good infection prevention and control in place, if you follow the regulations, if you have good antimicrobial stewardship – all these things in place – of course, that is an educational effort that costs money….Some of the countries in Europe where there are huge problems with antimicrobial resistance on the human side, if we look at the results of…point prevalence surveys…these countries in some of the hospitals, they don't have any infection control nurses or any infection control staff at all. So, the investment in that has a result, or has an effect if you don't invest. So it has a cost to…to-<br><br>F: So, for both of those then…with good farm practices then we need something coming in to that from the like the economic drivers, right? And similarly from the human side, if we are to have good infection and control practices, we need to have that system funded as well?<br><br>P: Yea. It has a cost. It comes with a cost.<br><br>P: That's that temporal thing too. It may come with a short term cost …but long-term [benefits]…<br><br>P: Yea, depends what your system…view.
	Workshop Day 1:<br><br>P: As was said before it does not help if you try to involve and hope that that somebody will train somebody else, because that is not [inaudible] so you need to get a proper running and maintain.
	Workshop Day 1:<br><br>F: And the settings, so just that I am clear, but health and social care, sort of system or setting, is the health care at large.<br><br>P: Yea.

	R: And the non-health and social care is the community?<br><br>P: Yea what we would call community in our own homes. Not necessarily care homes, because more normal health care is going to be delivered in people's homes, but it is still health care.<br><br>F: Right, but it is that other thing.<br><br>P: Yea. Yea.<br><br>P: Again it depends on the country I would say and the types of professions that you have when these types of care institutions, there may be some institutions where they have very few health care professionals, and more people who are not at all attuned to AMR, because they wouldn't have learned about that.<br><br>F: About more in the health care setting.<br><br>P: Yea. I am thinking about senior homes or institutions that are taking care of the aging.<br><br>F: And you see those as part of the community piece, or as part of the ...<br><br>P: Depends on the country.<br><br>P: Yea. I mean I clustered them under health and social care, because there is often an interaction through community nurses or GPs with residents within these homes, but also the care workers. You may have one registered nurse in the home, but the other, the care workers are predominately a non-registered or a non-qualified work force. So there are, there is a different approach needed to get that work force are usually quite poorly paid, quite transient. Um, so the influence is on AMR can be very different to hospital settings.<br><br>P: So, they don't benefit from the same educational offers perhaps also.<br><br>F: Is that something, we haven't really captured that right? So we've done the system, but then sort of that like staffing and different roles?<br><br>P: There is an issue particularly in terms of the migration of health care workers, which we see a lot of or who knows what we will see in a few weeks' time, but I know, but we do know from things like… that health

	care workers from different European countries come with their own individual perceptions and their own cultural influences that are not necessarily the same as those that we would have in the [name of a part of Europe]. So we have a bit of a rub sometimes in terms of we make huge assumptions that everyone understands or follow agrees with what we want in the [name of a part of Europe] and of course they don't and we don't address that.<br><br>F: And that comes into, we had travel here somewhere and I don't know where it is now, but might be, just that general movement of …<br><br>P: It is just general movement of people and employment.<br><br>F: And then you like also brought up their like this, like cultural aspects too and I think that may be comes back to your point [name of participant], that like the health care systems, the health, the health and social systems are different in the different countries and some of that probably has to do with culture…<br><br>P: Absolutely, yea.

THEME: Promoting Health and Well-Being	
**Sub-Theme**	**RELEVANT DE-IDENTIFIED WORKSHOP QUOTES**
Health promotion and infection and chronic disease prevention	Workshop Day 2:<br>P: We are snowed in for the prevention and since we are all …from the human sector it was easiest for us to see that, but we tried also to see that prevention. There is room for prevention also in the animal sector…. First we say prevention of disease that is, that can be achieved by increase and fulfillment, increased coverage of vaccination programs would have an enormous effect, big impact, but it would be relatively hard to do, because that is what we are all trying to do in Europe now, but we are not succeeding with it for example. So I think there it is.
	Workshop Day 2:<br>P: We need to go away from always using broad spectrum antibiotics to actually much more narrow ones, but then we need to know what kind of infection we are trying to treat, and much more focus on prevention of infections, hygiene sanitation, but also strong vaccination programs in order to secure that reaction in, in the best… of worlds.
	P: If you look at resistance, we are in a good situation. If you look at antibiotic consumption, which you can, you can also do that in total prescriptions per 1000 inhabitants for example, and then we are also extremely low, and we have been lowering it for the latest twenty, twenty-five years and we are doing it gradually. So we don't see backlashes. We don't see that this has bad effects. We don't see increased deaths in untreated [inaudible] for example, or we don't see that children mortality is going up even though we have reduced the antibiotic use enormously. So we are following that, but that is the easy part to see antibiotic total use. The more difficult part is the spectrum. We were talking about before, of broad spectrum and narrow spectrum, and I think that actually has a larger effect than the total use, because… am I allowed to take a second…Yea? Okay. If you treat for example now a cold that has become bacterial and so I have sinusitis, and in [name of European country] we have the tradition to treat this with penicillin Lin. [drug name]. Very, very narrow penicillin, and why do we do that? Because we know that the dangerous and most symptomatic sinusitis, they are caused by either demococci or sometimes in the same area, streptococci maybe acting up. They are the two dangerous bacteria. Then you have a lot of other bacteria, which like Moracella, like [inaudible bacteria name] and they are not often causing great harm. So we choose to treat the major ones. If we instead treat with a, and you had something in your food, yea<br><br>F: Ceftriaxone<br><br>P: Ceftriaxone, which I find they use so much in the [region outside of Europe] where I have worked now for a few years, they use this broad spectrum antibiotic, which of course it kills also this bacteria, but it also kills

	half of the stomach bugs, and so you are creating a vacuum in your stomach and you are letting whatever resistance you have, which you probably do, they grow and so you get much more resistance from this treatment, and so just like treating a simple upper respiratory tract infection, you can even though you get antibiotics, if you get the right antibiotic, as I see it, you still don't harm your gut flora, which is 1.5 kg. of bacteria, which is millions and millions of bacteria. So this is the, this is even more important to choose the right antibiotic and that is why this should be done, not by the pharma industry, which it is [region outside Europe], which is not in [name of European country], because it should be based on rational medical decisions, with the awareness of which one is broad, which one is narrow.
	Workshop Day 2:<br><br>P: We very rarely applying a lot of the preventative measures we know we could, regardless whether that is changing our role, our behaviours, strong vaccination and stuff like that, and vaccination in one place. I actually believe that relates to, to like foreign practices and stuff like that.
	Workshop Day 2:<br><br>P: what I miss is something that you talked about, prevention [inaudible] if we have healthy people, healthy animals, you diminish the demand… hopefully I would say, and I don't think you have it clear enough really that…that you must focus on health. What you talk about infection, prevention, etc. etc. etc. Then of course you have put a focus or something, that it is our problem, otherwise, and retain of [inaudible] any kind of animal products in that time, that has of course focused on low prices, high efficiency, and it is not an easy way to solve the problem you talked about. So that of course is, oh, very important link to discuss the price of food.<br><br>P: And animal products and of course sitting in the… engage in animal welfare, I think there must be a very focus on the welfare, because you have to, if you have to, prevention is so important.
	Workshop Day 2:<br><br>P: The… the way we reason in [name of European country] is healthy animals don't need antibiotics, and healthy animals we get that, not only by good hygienic standards, but also, but good living conditions for animals? I mean, good stables, spacious stables, etc.<br><br>F: So I think if we, just good farming practice?<br><br>P: Yes.

	F: And that includes management, hygiene,<br><br>P: Not only hygiene, but also nutrition.<br><br>F: Nutrition, yep
	Workshop Day 2:<br><br>P: Yea, I am talking about animal welfare part, and I saw that you included the, for instance animal density, but it is a lot other thing that also should be included in the animal welfare part. For instance the possibility to behave naturally. To have routine material for pigs. To keep the animals in stable groups, and, yea.<br><br>F: Could that fall under sort of what we have got there.<br><br>P: Yea, yea. Connected to animal welfare. Yea.<br><br>P: Definitely.<br><br>P: And don't forget feeding, feed of course is a very important factor… the composition of the feed is really influences for instance the amount of tail biting. How you compose the feed, the composition of the feed. So and then you have the air quality. You have… water supply, and the rearing system, and if they have… solid floor to lay down on, or if they have slated floor.<br>Yea, it is a lot of different aspects on animal welfare, and animal welfare…Animal welfare….is a way to reduce antibiotics from the bottom. I mean it is preventive. If you have a good animal welfare, you need a lot less antibiotics, because they are feeling better. The stress level is going down, and they are more healthy.<br><br>F: Should we talk about animal welfare and health promotion pieces within the same thing?<br><br>P: Yea, I think when you talk about here the consumption of food and nutrition composition of diet and so on, that is for the human part and you need it for the animal part as well.
	Workshop Day 2:

	P: Yes. Add to that the family who cares about these animals, if they can get stable and healthy, of course the family have to need that first. So but if you don't need to have animals to have a stable family, because that is, I think this is the thing who is a level of stable, calmness, trustful, that feeling in families, between people, that is a big problem. So we need to make stable families.<br><br>F: I think you are drawing a connection between the mental health of the people, and this case is simply producers and the health and wellbeing I think of the animals on the farm?<br><br>P: Yea.<br><br>P: And if I could add to that, I totally agree because we have also some scientific works about, studies about the proudness for the farmers, and if you have very good animal welfare, the farmers are more proud about… their husbandry and everything. So the farmer situation is one. So it is all linked together, I think.
	Workshop Day 1:<br><br>P: So well basically, you know, people living well are less likely to need an antibiotic. We brought that up earlier. You know, you… you said there are links between infections.<br><br>P: Well it can be many things.<br><br>P: Infections.<br><br>P: It is physical and mental health actually.<br><br>P: Absolutely.<br><br>F: So improved physical and mental health well-being?<br><br>P: Yea.
	Workshop Day 2:<br><br>P: So I was thinking in terms of like the long term prevention of human use of … '<br><br>F: antibiotics? antimicrobials?

	P: yea, yes, but also in the long term in the next step, like our relation to what we consume and how we produce food in different ways. I am wondering, I don't know the data about [name of European country], but I have this guestimate based on someone that knows, or you can find out, like what is, how are people feeling in [name of European country], like in terms of the stress level, depression, psychological wellbeing, because I know my own experience as a young kid and older kid back in the day, I was incredibly stressed, focused on like producing stuff and not getting well, and then I didn't care what I ate and I know a lot of other young people who are not necessarily feeling super well and I heard now in day care, there they have these, you don't go to day care as a three, four year old just to learn and develop, but you also are evaluated. So I am wondering how are the systems that we live in affecting like long term preventions perspective. Our stress levels, and how is that affecting us.<br><br>P: You have health promotion, because everyone I guess… and in my opinion of course regardless of what price your ill health of not feeling healthy in a psychological way. Of course you could imagine that at least in a suppressed immune system it is easier to get infections and to being treated with antibiotics.<br><br>[P: exactly] So of course I was saying to cycle back.
	Workshop Day 2:<br><br>F: ….So the psychological wellbeing or the mental health or the stress level piece.<br><br>P: Yeah.<br><br>F: And I think [P's name], just about sort of like we are talking about sort of non –communicable and communicable diseases and …that maybe that increases both potentially, but you also talked about nutrition, like the diet we choose.<br><br>P: Oh right, exactly, because like I am going back to my own example. I used to go like all time when I got a cold, I was like please treat me. Do something, and then I realized after many years, oh I am just too stressed. I should just rest. So which I started to do and nowadays also like I feel much more connected with, what do I eat, what do I choose. I have problem sometimes and I know other people too, buying food, because like what am I buying. Where does this come from, and I am also too more connected to like the trees and everything and that is the kind of awareness change as well. Like how am I feeling as an individual and then how we are collectively feeling?

	Workshop Day 2:<br><br>P: if you look at infectious diseases, you have of course the hard diseases. You have septicemia. You have pneumonia, and they kill you whether you are Swedish or Zimbabwean and how often you get them can be dependent on nutrition status, but then we have these soft diseases, which we are going to the primary health care centre for most of the time, like it is a cold, upper respiratory tract infection or, or it becomes sometimes bacterial sinusitis, and these are the ones I think where you have a lot of gray space and they depend a lot about, a lot on your psychology, and your stress level, but they also depend a lot on nutrition level, and hard health stages, so to say. So we are less prone to suffer from such infections I think than… than malnourished in African,
	Workshop Day 1:<br><br>P: [name of participant], I think an example, taking us back to the nutritional food would be if your diet is less nutritious you might be more likely to get Type II Diabetes, which can lead to a whole series of negative health care outcomes, ulcerations – these sorts of things all lead to need for us of antibiotics.
	Workshop day 2:<br><br>P: But what I mean is there are two dimensions. There is the psychology of stress factor and there is the basic more top heavy nutritional status, I mean. And health status as a whole.<br><br>P: The nutrition status, would you also say that we in general would have a more suitable or better microbiome than your African friends here to hopefully help us to have better immune systems to withstand infections …<br><br>P: That is the next, and very important and very, very interesting question. I would say that the microbiome depends on where you are in both of these settings, in [name of continent]…the microbiome depends so much on your consumption of antibiotics actually and there are [name of nationals of a European country] who have a very virginal flora and microbiome, and there are surely also in the far off I would say more rural settings in [name of continent] you would find people who have a perfectly sensitive microbiomes, but if you, if you are consuming a lot of antibiotics whether we are in [name of European country] or you are in [name of non European country], you will ruin your microbiome, and that… I would say that we in Europe, are actually still consuming more antibiotics even though we, so probably hasn't in the general situation I think we have a tendency to disrupt our microbiomes more than other continents.

	P: I would agree.<br><br>P: Yea. Yea.
	P: Can I add something to that, because I had actually had like one personal experience that happened, because I mean I have parents from [name of country outside of Europe] and I grew up in the [name of country outside of Europe] and in both those countries, I mean you get antibiotics quite easily and the thing is I got like an ear infection a couple of years ago…and then my doctor [in Europe], he was like when I asked for antibiotics, he is like, 'oh, antibiotics!' Like I asked for poison. For me it was like 'yea'.<br>I actually like quite got a lot of antibiotics [as a] child…I wasn't thinking, but it is like different kind of like views on how dangerous antibiotics can actually be.<br>And then you have this like resistant to yourself towards those. So they started like prescribing like mild antibiotics under two years to me, that the infection got so bad, that it couldn't be curable until they almost killed me giving me antibiotics after like two years. So I couldn't like even move like for two weeks and I was done with the dosage of the antibiotics after I used that, because it actually got [me] like more and more and more resistant… So it was like one thing that is like maybe like the culture difference, like how much resistance you yourself have towards…to taking, because you mean, maybe like your…your body or like the kind of like the [microbiota] in your body are more resistant than the other people because of where you come from or how high your resistance is.<br><br>F: And your previous exposure?<br><br>P: Yea, exactly, and then one more point that is maybe also, if you could capture that.<br><br>F: I think it is just…previous antimicrobial exposure?<br><br>P: Exactly.<br><br>F: And I think that then is going to affect, well it is not going to affect use, it is going to affect the success of that? So it is sort of the efficacy like whether or not the treatment works?<br><br>P: Right.

THEME: Social and Economic Conditions	
**Sub-Theme/Sub-Topic**	**RELEVANT DE-IDENTIFIED WORKSHOP QUOTES**
Equality, inequality, food security and AMR	Workshop Day 1:<br><br>P: …I put social inequalities um… which links into a lot of the food things but actually does feedback into human infections and I divided it out into health and social care and non-health and social care.<br><br>F: So can you explain that? What's the difference?<br><br>P: So what we are finding out from some of our data in [name of European country] is that some of the patterns of resistance are linked to social inequalities – to areas…that have deprivation so that feeds back into the health and social care system so you have this cycle.<br><br>F: Ok, so if I just call it the health and social care system<br><br>P: Yes.<br><br>F: So the social inequalities are having an effect on the system and on the diseases.<br><br>P: Yes, because it impacts your non communicable diseases which then has an increase in the burden of health care and the whole cycle -<br><br>P: So are the social inequalities due to a lack of let's say information or is it an economic question?<br><br>P: It's economic. [inaudible], but then it has an impact on education and the whole cycle that goes with that.<br><br>P: Because I think if you are more educated and are more aware of the impact of things, you will treat yourself better.<br><br>P: well you can be educated but still not have money to buy the food and everything.<br><br>[Many Ps agree].

	F: So this social inequality, we linked it to the social and health care system, chronic disease, infectious disease.<br><br>P: Uh health care costs.<br><br>F: Um health care costs…right here? And food security?<br><br>P: It's nutritional composition of diet.<br><br>P: Yeah, I linked it to nutritional composition of diet.<br><br>P: Nutritional quality isn't it.<br><br>P: Nutrition and AMR, what is the link?<br><br>P: Well if your diet is more nutritious? I mean you can have a secure … source of food but -<br><br>P: insecurity, food security, and nutrition I think are different.<br><br>P: It relates to human well-being, I mean your status of your -<br><br>P: Yes, its public health.<br><br>P: exactly you're more resistant, less vulnerable if you have a balanced diet.
	Workshop Day 1:<br><br>P: [name of participant], I think an example, taking us back to the nutritional food would be if your diet is less nutritious you might be more likely to get Type II Diabetes, which can lead to a whole series of negative health care outcomes, ulcerations – these sorts of things all lead to need for us of antibiotics.
	Workshop Day 2:<br><br>P: To tie this altogether perfectly [Group laughed]… Okay, if we look at another level of [inaudible] equality, so also in the conflict term, there is this wave of inside in terms of one of the main driving factors structurally in relation to attitudes and values and norms in society is when there is inequality, like gendered

	inequality, but also other types of inequalities, and then with those equality we less political violence…I mean we can change our relationships in terms of equality or inequality I mean in many different types, social, economical. We can change them in different ways, but if we don't also feel the equality, then we won't have the same effect, and I think that is where we come, I think this is the important part, also you said [name of participant like how we relate to the animals. How we related to what we eat. How we relate to this planet and each other, like and it is a very big thing, but I think it is important for the larger…<br><br>P: So again framing wise I think we should if we want to work on prevention, I mean it is important to be aware of both I think, because but if we want to work in long term to make this world more healthy, I should think we should talk about equalities, because, but I don't know, I mean who cares.
	Workshop Day 1:<br><br>P: back to social inequalities … we talked about high end welfare…so well basically, you know, people living well are less likely to need an antibiotic.
	Workshop Day 2:<br><br>P: Many talk about like this nexus of food energy water, which is all interrelated in that sense. But equality is very important in case of food security, because for example when you have like an economical advantage, your country tends to be food secure. Like when you look at the map for food security globally, usually those countries which are green are the countries with high GDP, even if they are not producing enough food in their own like borders. So, I mean I am paying $5.00 versus $1.00 that you could pay for the food. So obviously I buy from your neighbour which is closer to you.
	Workshop day 2:<br><br>P: …also like hand in hand with inequality … like how much food we have available, that actually some [inaudible] centre has done a study of how much food we have available in [name of European city] if we close down the borders today, seventy-two hours. After that people move to start eating each other. So I mean it is a kind of like a defense related security of food that the government of [name of European country] has started with a, what should we call it, leaves me at resistant, like foods… strategy… food supply strategy for the country, which is like we need to become more and more self-reliant in case of food production, because we import like 60%, 70% of the food, which is produced. Yea, I don't know, like which products. I mean this is the thing that I [read] in [a] report. So and they compare like to the [name of Northern European countries] and say like, they are producing like five times more than they need. So that gives you like an upper hand.

	So I mean in that sense when you connect it to inequality, then it actually gives you an upper hand to decide like if you want to have say food or not. Because first you reach security, which is exactly the thing that [name of participant] brought up, that when you reach a level that, okay, you remove that issue, because now you are secure, you are not under the hunger line anymore. You have the opportunity to go above that, then you get to decide. So it is kind of like a Maslow pyramid approach towards food, that, okay first I was hungry, whatever you give me I eat. If I am poor that I am producing whatever way that I could do, and if I need to use antibiotics to produce more food, then I do that, no problem. But, when you get the luxury of picking, so it is also like a… like a… systemic thing that affects the whole thing. Like the affordability, inequality, and what kind of approach to food security we take.<br><br>F: So are you making a link then, I think I heard between food security and like consumer demand, or demand for product?<br><br>P: You could say customer demand as well. I mean obviously you could relate to that, because then as a part of society, you get to have like higher demands of better food as well, but it is more or less like a national things, like the system demands better food for its people, especially if you have like a system which is a bit more like left wing politics, that the government is actually responsible for the cost, for the… for the wellbeing and you know welfare and healthy, healthiness of the people, because it costs you. So when you, I mean these are like really easy examples that you could bring up like compare the size of a [name of food product] in [name of European country] with one in [non-European country].<br>I mean the first [name of food product] that I got [name of European country] was a bit like…'that is a [name of food product]?', because [name of food company] doesn't get to sell like big food to people and a lot of sugar, because if they get sick, then the government needs to pay. So obviously government puts regulation on that.

THEME: Research	
**Sub-Theme/Sub-Topic**	**RELEVANT DE-IDENTIFIED WORKSHOP QUOTES**
Developing new antimicrobials	Workshop Day 1:<br><br>P: ….I don't see the pipelines for new development of new antimicrobials anywhere, and if you are consulting with anybody from the farm industry because it is always this big debate about how people attract the disease. How many problems are there, but if we are being serious about it and we need to … so important to all of us and an almost number of public health problem number one, should we come to a decision at some point that this is something that governments should directly fund and forget about any private industry involvement. It is really that crucial to all of us, but you know this big issue with, I can't remember the name of the company in the States, but being subsidized with millions and millions of dollars to develop new drugs and even without massive public input of money, they bankrupt a few months ago. So it has been really a huge static bomb for any potential investor that want to hear about development of new antimicrobials anywhere. So if that is really the situation that even if this makes models with public- private partnerships, we cannot advance .... in order to think that we cannot afford to do anything. So somebody will have to put the money on the table. It is like when penicillin was discovered, it was produced in governmental facilities and then it went to kind of pharma industry. So is that debated somewhere when a decision is going to be made about this. Perhaps nobody here is heard that.
	Workshop Day 1:<br><br>P: but I know there is quite a bit of work done on looking at incentivizing the innovation system through public-private partnerships, because of the cost involved are humungous.
	Workshop Day 1:<br><br>P: ….pharmaceutical companies do not have an interest in maintaining certain operations which are not economically viable. So it is finding new ways of let's say compensating industry for maintaining a non-profit.

	Workshop Day 1:<br><br>P: You don't have to use as much antibiotics or antimicrobials but we do that, and we need to understand, when we talk antibiotics it is completely different therapeutic field like any other thing in… in the health sector, in all other places actually we develop new better drugs and perhaps we phase out the old ones. When it comes to antibiotics, we need the entire portfolio, so we need to find ways to both safeguard the existing…ones because we will need them also in the future, but also find of course new business models to come up with new antimicrobials…
Developing alternatives to AMR	Workshop Day 1:<br><br>P: Maybe there is some part in the picture here but maybe, I think we should talk about… research and development as one of the major issues of the ….what science and innovation can do and the other problems we are trying to tackle…and to me this is related to the development of other AM …..
	Workshop Day 1:<br><br>P: I absolutely agree. I also think it is part of not putting all our eggs in the basket of new antibiotic drugs, and having, and also not ignoring microbiome, because it is so, you know as you said, it is so integral to health in so many ways that we have yet to understand.<br><br>F: So that would be a priority?<br><br>P: Yea and I think we need to understand the microbiome more, because my gut feeling, excuse the pun, is that, I know it is pathetic is that that is where we need to be heading in the future.<br><br>P: Yea. I agree and actually when I was looking at this before, we have got human microbiome up there for the animal one. It is just as critical. Top right, you have got human microbiome somewhere.<br><br>F: Changing gut microflora.<br><br>P: Yea. So we are doing that in … I am sure but they are also doing it in terrestrial systems as well….<br>F: Gut microflora? ….<br><br>P: Cattle in particular.

	F: …this is like all things with guts, not just humans.<br><br>P: Yes. That is it.<br><br>F: ...And I'm just trying to make this… this is… all things, not just humans, and I think there is, so we have got like vulnerable populations and nutritional composition of diet and I think that is human but I think that is also the animal diet?<br><br>P: Yea, feeds, yea.
	Workshop Day 2:<br><br>P: Yea. Then of course… there is some sort of like futuristic product that is coming to the market, that is not yet like regulated and you can talk about future food, like you know treated, printed food, or talk about insects. You talk about algae's and many kind of like this kind of like future that is coming to our system, which is not yet tested.<br><br>F: …We have got sort of these new, new food systems. Yea, new food. I will just put like an example, 3D and insects.<br><br>P: Yea.
	Workshop Day 1:<br><br>P: …Genetically modified foods…
Better Measures and Data	Workshop Day 1:<br><br>P: We don't actually have precise figures on use. Most of the figures used are based on sales from pharmaceutical companies, or from prescription figures from definitely surgeons, or doctors and so on and they are very broad aggregate figures. How many of those are actually used, we really don't know. We just assume that the sales figures are a good proxy, but yea it is true, And we don't really know how much actually go into the environment through residue or waste that go on like this, and you know<br><br>P: And it's true, when you have minimum pack sizes and you buy, you have to a buy kilo of an antibiotic, and you only need a few grams, but then are you supposed to throw that away. This is a problem for fish

	farmers for example. If you have to buy a kilo, to use a few grams, then they chuck the rest away, but that goes down to one kilo of use in the sale's figures, but it is not
	Workshop Day 1:<br><br>P: Again for the accuracies, we have shown Europe that there is, although it was mentioned, the accurate reports, nice correlations and you can see the benchmarking and areas where there is conservative approach to use, having much lower levels of resistance in general. So okay the systems are imperfect, but at least show that we are kind of in the right direction. It is better to collect the data than not having anything. …..
	Workshop Day 1:<br><br>P: I think that it is correct about the animal use that it is a new system and it needs perfection and on the veterinary side it is difficult to get the more accurate. It also falls on the human side of course, but just as well as when we talk to microbiologists about our surveillance systems for antimicrobial systems, and if some microbiologists as soon as they realize that the samples may not be taken the same way in each hospital or the cut off, for when you take a blood sample it is not the same. The immediately say, it cannot be used. You cannot compare this data. And every time we have to say, well this is the best data we have. Let's try to make the best out of it. Let's try to conclude as much as we can putting the disclaimers that this may not be fully comparable, but it is the best we have, and then as [participant] said a couple of times already, sometimes we need to take action, even though we don't have an absolute guarantee that when we do this, the effect will be that, and then otherwise we die before we have taken any action. Right.
Generating evidence versus taking action and AMR.	Workshop Day 1:<br><br>P: Research and development …. I think it does fit in another way in control options, because we know, just looking at this *(the model)*, at once you realize that this is a complex problem, and so the situation is so urgent that you can't wait, if you have full proof that there is a connection in order to act. Otherwise we will be dying without doing anything, and there is a certain tendency from some sectors to hide behind the non-existence of for example perfect risk assessments and not wanting to act and the environment is perfect example for that, there is a push to start surveillance in the environment to do more, but there are people saying there is not enough evidence in the environment is [inaudible] therefore why should we invest any resources, and that applies to everything. So I think research and innovation is too important to understand more the problem, but it should not be a blocker for proper risk management actions when we have already enough evidence. So I think the question is what is enough evidence to act, and when should we decide when it is not appropriate to wait for more evidence, and I think that is a bit of debate. For those who have

	been working in risk assessment, with a more kind of sophisticated methods, quantitative, more than etc. etc. we realize that is not going to be the answer to this such complex problem. You cannot redesign a model to give you the perfect system where you can plug in different controls to see exactly what is the effect of the intervention, if that is not worth that.<br><br>F: So, because what we have done right here, the research and development is specific to the development of alternate antimicrobials, but what you are getting at if there are all sorts of research and development probably into good farm practices, in terms of disease control, um, In terms of the infection, prevention and control on the human side. So can we link that to all of those things.<br><br>P: And understanding the environment, what is going on in the environment.
	Workshop Day 1:<br><br>P: I guess what you are saying is some kind of clues about where your actions should be targeted. I know that that is obviously an idea evolved based on some evidence, and the assessment of the level of risk. What I am saying is that I am not sure if such a complex context that is always possible, and I mean somewhere you need to draw this line, because there would be many situations where one needs to take action without the evidence. We have seen it in Europe for example with the establishment of targets of use. Sometimes we see it without really having a full risk assessment done, and then only after implementing a measure they have seen the effect of that issue. So this is a different way of looking at it, and the investment, would of course with an investment done without the full guarantee that the return will be there, and politicians do not like this.<br><br>P: The political level of course is the trigger. [The political level] needs to be convinced before we have any regulatory change and for sure regulation is a major determining factor for antimicrobial use in both animals and humans.<br>P: Yea, it is really, really important, because they are the ones that connect with so many things and control the budget. So the research budgets need to be increased, and also money needs to be released from different funds or aligned if the environment and the health budget where there are co-benefits. That is not done currently. So if we going to move faster, than there has to be a political alignment [between government departments], where those co-benefits exist.
	Workshop Day 1:

	P: I mean you know we know actually nothing about really what is going on in the natural environment, largely because much of that research is just not been funded. You know, funded, we are starting to get some more funding in the [area of Europe] for that kind of thing, but you know even now it is very difficult to get funding for antimicrobial research in aquaculture, because it is perceived to be a much lower risk than terrestrial livestock species. Right and then it is another step down for the environment, but it is slowly changing.
AMR in aquaculture and environment	Workshop Day 1:<br><br>P: There is. I mean you're absolutely right. I mean the fish farming aspect can go into all of these aspects. But there is one big difference which is the risk of…the risk of AMR transferring from a terrestrial agriculture species into a human is far greater than what you get from a fish species because of the nature of the pathogens, which would be very difficult to capture on here. And that's why the regulators really haven't concentrated so much on either the environment or the fish farming because it's not really zoonotic pathogens there in the same way you get with the poultry and beef etc
	Workshop Day 1:<br><br>P: I think that's very right distinction that the pathogens of the cold water aquaculture are not the [inaudible] to humans, but I think at this time there is a lot of publications of the presence of resistant determinants, resistant bacteria from water sewage etc and whichever way we turn it, the agriculture production where lots of antimicrobials are immersed in the water has a huge environmental… when the time comes when we will start having real data on the prevalence of resistant bacteria, resistant determinants in the environment to the larger extent – now we have excellent data from the veterinary side, the human side – but from the environment… water and soil, it is still, very few surveillance systems will collect this data and I think once this starts, there will be much more interest in what are the effects of all these antimicrobials in agriculture put into the environment from pharmaceutical production waste products going into the environment into the sea.
Treatment Guidelines	Workshop Day 1:<br><br>P: There are a group of nodes missing about antibiotic or antimicrobial stewardship for food producing animals. I think the development of treatment guidelines for animal diseases is quite an important component, and it has important links to training activities for professionals, for…students, medical professions – it has a very clear link to proper diagnostics and proper sensitivity testing and most importantly it has a very clear link to development of lists that rank different antimicrobials, and these lists should consider or need to consider human health elements and animal health elements and most of these

	are in contradiction so the same drug is important but for different purposes, so all of these things have be really, should come together into these treatment guidelines and I think it has very important links to livestock illness.<br><br>F: So the treatment guidelines will effect on farm use?<br><br>P: Yes, and livestock illness.<br><br>F: And treatment guidelines will effect illness?<br><br>P: It will help impact including use and tackling how to treat the animals when they become ill so you should consult them before embarking on antibiotic treatment.<br><br>F: Yes, I was trying to clarify whether the treatment guidelines will affect use or would they affect the prevalence of the disease.<br><br>P: They would because if used in the correct [inaudible].<br><br>F: So is it through use that it affects illness or is through the guidelines.<br><br>P: It is mostly through use, it's through the choice that you make with the antibiotic.<br><br>P: And the treatment guidelines are affected by diagnostics?<br><br>P: Yes, as a component of that.<br><br>F: And, education goes probably to both of these?<br><br>P: Yes. Um and there is the development of lists that rank – the WHO lists or the [inaudible] lists of Europe or any lists that exist. They should be considered in the treatment guidelines.<br><br>F: Does it have a link to human use as well?<br><br>P: Yes, there are a whole cascade of factors that are very similar…

	Workshop Day 1:<br><br>And it is what [name of participant] said before, if the treatment guidelines are followed, you have a more effective treatment, you get less burden of illness, you reduce the use of antimicrobials and so on and so on.
Diagnostics	Workshop Day 2:<br><br>P: I always tell when I discuss AMRs, of course everyone understands we need to secure responsible use. In my opinion that is very restrictive use, and in order to achieve that, we need to use better diagnostics, so we actually know, what we are trying to treat. We need to go away from always using broad spectrum antibiotics to actually much more narrow ones, but then we need to know what kind of infection we are trying to treat
	Workshop Day 2:<br><br>P: So there is a lot of those things, like fast and accessible diagnostic kits.
	Workshop Day 1:<br><br>P:… we haven't mentioned new technology… Well E-prescription, we mentioned earlier, but tracking of prescriptions, and things like that, but the whole move towards let's say I don't like the term, but personalized medicine and looking at the genome.<br><br>P: Genomics.<br><br>P: Genomics, exactly. All of that is being driven forward.
