# Supplementary material for: Factors influencing antimicrobial resistance in the European food system and potential leverage points for intervention: A participatory, One Health study
Source: PLoS One. 2022 Feb 22;17(2):e0263914. doi: 10.1371/journal.pone.0263914 (PMC8863257; doi:10.1371/journal.pone.0263914)
Supplement: S2 Table — (PDF) [file pone.0263914.s002.pdf]

S2: Quotes per overarching factor

Please note:
Quotes per row often contain the conversation of multiple participants. In keeping with the University of Waterloo Ethics requirements, we have labelled each contribution per participant as "P" and for facilitators as "F".

OVERARCHING FACTOR: Agreements, Standards and Regulations	
**Sub-theme/Sub-Topic**	**RELEVANT DE-IDENTIFIED WORKSHOP QUOTES**
Regulations and AMU and AMR and consequences	Workshop Day 1:<br><br>P: I think it was said before the growth promoter should be … at least that is not an issue in Europe.
	Workshop Day 2:<br><br>P: I just wanted clarify, you have those use for growth promotion and that is not legal in Europe anymore.
	Workshop Day 1:<br><br>P: I am not sure that that importation of the products is quite such a risk for EU in terms of you know the quite strict importation, guideline systems in place… higher, higher (on the model). So yea that is it. Yes. I mean I think our food safety systems are pretty robust for imported products in terms of, they have to be produced, you know to align with EU standards. So I think that is probably quite a low risk on that diagram. I know we have already touched on that.<br><br>P: But it comes at a cost.<br><br>P: I disagree with that, I don't think there are any regulations that protect us from AMR issues. There is nothing there. There is a lot of things about ….<br><br>P: Residues there is.<br><br>P: Residues is the only thing, but it is a totally different thing. We don't have any antimicrobial resistant bacteria … We don't have surveillance systems. We don't know. We don't test for any product. That is a big thing in this …development because even labelling countries, whether they have kind of beginner to advanced surveillance systems it is a big thing for them, because they don't want a label attached, because

	this may have trade implications. So imagine if they are testing out the products, this may totally… so I think we have information about what is coming into Europe in terms of load of resistance and [inaudible ? [there are other] many things] and we just don't know.
	Workshop Day 2:<br><br>P: To other experts, which I noticed and it is important, a natural trade agreement would include restrictions on the use of antibiotics for animals, and I think we… I would mention WTO as something that they should convince. … I think that is important too that we could [do] more to convince WTO that handled this [inaudible] agreement, that includes more of pesticides and all that kind of thing, but… it is included in the agreement.<br><br>F: So is it, it is not specifically trade then necessarily. It is international agreements on different practices.<br><br>P: Yea.<br><br>F: Okay and the WTO is one example.<br><br>P: Yea.
	Day 1 workshop:<br><br>P: [Name of European country] is one of the extreme positive examples of the world. We started long ago. We started like in the early nineties. On the vet side even in the eighties, and not only because of that, but probably partly because of that, we had today an extremely good situation when it comes to resistance, and you can compare it for different bacteria, resistance to different antibiotics, and there is someone now, actually during 2019 who has tried to boil down all the resistance is to all bacteria into one score, to simplify it, and then [name of Northern European country] comes out on top, India comes out in the bottom.<br><br>I mean so and that, there are other such maps mapping the situation globally and in Europe, and it is obvious that we are living in a country with extremely privileged situations when it comes to resistant, resistance….[Different countries in European] fare a lot worse than [name of other European countries]... and….it is much antibiotic policy.

	So we [name of European country] are low in antibiotic use, and that is the same for [name of other European country], at least for human use. They have an enormous veterinary system, because they have much more animals, and what more, there was one thing. Um… nope, okay that is enough. We are living in a different world than most other places actually yes. So we have also a lot to teach and a lot to show, and a lot to share. I think that is probably why you are here also.
	Workshop Day 2:<br><br>P: I would really like to add… [the differences between] … [name of European country] and ...[region outside Europe] where there are no regulation …between the industry and the decision [makers], and one of the reasons why [there is] a very, comparably very rationale model of antibiotic therapy in [name of European country] is because we have such close, such strict regulation of how industry can work with decision makers. So that is one of the assets of the [name of European country] model actually and one should be very careful to disrupt that.
	Workshop Day 2:<br><br>P: I don't know if this is something that has an aspect on this, but [name of European country has] ethical rules on how we can interact and meet with physicians and you know getting access to them, and that is something that [P's name] also thought about. So you have to understand it is difficult for pharma companies to get access to health care professionals or physicians to talk about products, etc. and I was just thinking about you know informing perhaps or providing information to physicians about other types of alternatives, etc. because we are really talking about, if they prescribe one type of product that is more, gets more resistance than others. I don't know, because I don't know that much about AMR, but maybe that can mean something …regulations access to… access to health care professionals.<br><br>P: I just have a comment or a suggestion. What is your intention to increase the access of industry to the physicians? Was that the aim of your kind of proposal?<br><br>P: My comment?<br><br>P: Yea.<br><br>P: No, just you know I was saying that… as I understand [it], the farming industry [in name of European country] has a difficult time coming to the [acronym] as we call them, just to inform them about products,

	etc. so I think that I mean yea maybe that can be a hindrance to talking about other alternatives, etc. and getting the message out. So…<br><br>P: And especially if our medical experts are insightful and to speak with professional decision, but we need to… stay very close to… to the approved texts from the agencies. We could never ever stop discussing any use. Of course that is a crime. So, these kind of ideas around what could be done instead of using [antimicrobials]…the only approved thing we have come from MPA is that you should use this and then we are allowed to… it is a crime to say anything else.
	Workshop Day 2:<br><br>P: I would really like to add… [the differences between] … [name of European country] and ...[region outside Europe] where there are no regulation …between the industry and the decision [makers], and one of the reasons why [there is] a very, comparably very rationale model of antibiotic therapy in [name of European country] is because we have such close, such strict regulation of how industry can work with decision makers. So that is one of the assets of the [name of European country] model actually and one should be very careful to disrupt that.<br><br>F: Is it the same on the veterinary side? Does anyone know? Are they allowed to market directly to the veterinarians?<br><br>P: I mean they can come and have product information evenings when they talk about products. Yea.<br><br>F: I am just curious if there is the same legal framework there for veterinarians.<br><br>P: …It is also in…within the veterinarian side. It is so important.<br><br>F: Okay. So it is the same sort of rules and regulations in the conversation.<br><br>P: Yea.
	Workshop Day 2:<br><br>P: …We know we do not want to disrupt any of the ethical rules that we have today. What we just need to understand that when we talk about this, this consensus tripod culture…in [name of European country], and everyone talked and try to find good common solutions together. When it comes to this regulation, [it

	is there] for good reasons, absolutely…but it is also sometimes could of course limit the opportunities to find a common solution to something like this. There is good and bad always when not allowing communication.<br>P: Oh, but I agree that the strictness has also some difficulty. It incurs some difficulties when we try to do these discussions on the national level and that is a problem, but… but we shouldn't mix the two I think. I mean we should not let the difficultness at the national level influence the strictness that we now have at the local level. We have the drug committees, which were established in [name of European country]… to be the ones writing and the local recommendations to the physicians. While twenty or thirty years ago, the traditional treatment was mostly formed by the industry, and of course the industry has an interest in net gain and so that influences, influence here and influences still in many other countries that prescribers' pen, and that is not what is rational from a medical point of view. That is what is rational from an economic point of view and, and you have to keep this in mind.
Differences in agreements, regulations, and standards relevant to AMU and AMR between countries and consequences	Workshop Day 1:<br><br>P: But whereas growth promotion in the EU was banned in 2006 if I remember correctly for all animals. [A few participants agreed]. But of course that is not what's happening in countries around the EU and we talked about this transboundary issues. How effective is it if you're not using it [regulations] in one area and your neighbouring country is, so having this transboundary – whether it's due to international trade, whether it's due to migratory animals and birds, how do you handle that?

	Workshop Day 1:<br><br>P: We had something about the selling of antibiotics, and profits derived from that and how that differs between countries.<br><br>P: It affects the use on both the human and the animal side of course.<br><br>F: So profit. Would that capture it? Profit for sale.<br><br>P: I guess.<br><br>P: Yea, because there are different systems for how antibiotics are sold. Right. Even just to the consumer. Some countries in Europe, the doctors themselves will get you know financial profit for selling, but in other countries that is not the case.<br><br>P: Right.<br><br>P: Wherever you have regulation, the legal regulation of the use in animals and humans that then affects the profit possibilities, because it will be laid down by law who can sell.
	Workshop Day 1:<br><br>P: Just on the regulatory side we talked about a few minutes ago here in the EU, I understand all antibiotics for humans and animals are by a prescription by a medical doctor, veterinary doctor or veterinary surgeon. So it's the professional vets and professional doctors who have to give a prescription for use. Of course, we don't take into account black market operations or internet sales and stuff like that which are tricky. So in Europe, it should be relatively easy to restrict regulations on prescriptions – prescription control…. Where other parts of the world there aren't prescriptions – they are over the counter.
	Workshop Day 2:<br><br>P: I think…one question is also for instance in [name of European country] and I think also Europe nowadays recently, you cannot buy and just going into a store, but I know in many other countries you can buy antibiotics yourself. You do not even have to have a prescription. So, I think that is a very, very important.

	F: So is that, does that come back to sort of regulations?<br><br>P: Yea, they have had that regulations, I don't know, could be [inaudible] they have it for a very, very long time. I don't know if you ever could buy antibiotics without a prescription in [name of European country]. Not for humans and not for animals.<br><br>P: That in other countries. I know it is recently in Europe. I am not sure it is decided yet? Or… I know it was …<br><br>P: It is an evolving process in Europe you might say. I mean there're, countries like [name of European countries] they have it on paper, but it is not implemented, and [name of European country] I think has taken exception to the law, regulation a number of times, but still you can go…there and buy it, so it is still to come. So it is a matter of implementing.
	Workshop Day 2:<br><br>P: …with [name of European country] and Europe…most seafood is traded, and we get a lot of our seafood from [region outside Europe], and historically we have always seen it as a bit of a privilege for them to sell to high income countries, because they always get a premium, they get paid better, but will be here for more and more processes now, and anxious that they say that they are, they would rather sell to [name of non European country] because they…got more purchasing power…. They don't have to deal with the issues of screenings and customs.<br><br>[Another P: hm-mm].<br><br>So they would rather have the security of selling to [name of non European country], and they are therefore willing to sell at a lower rate. So… and you know there it's kind of, it's a staggered flow to [name of non European country]. We get paid a little bit less, but we will not lose any customers.<br><br>P: Risk management.<br><br>P: Yea. So it is really a question for us in our seafood in [name of European country]. Like how do we, how would we draw the lines, because if we are strict and we are too proud, it might, you know, just erode outside of our borders and we are so interconnected with the rest of the world, so…

	…I mean the question is what happens when, when we, you know, if purchasing power keeps increasing in [name of non European country], then suddenly there is very little incentives for anyone who make it to sell shrimps to [name of European country]. Are we then [going to] become more relaxed with our requirements so that we actually can buy shrimp from abroad? Or are we just going to stop buying shrimp?
	Workshop Day 1:<br><br>P: I think this issue that now [name of participant] also elaborated on that, I mean it is an issue which is well known in the, among the animal industry in Europe, at least in North Europe, right that we are putting various restrictions on them regarding … bacteria, and antimicrobial use and so on in order to reduce the risk of the products that end up for the consumers in the shops. At the same time, it is a huge import of chicken meat, beef, even pork from [non European continents and countries], which are produced under completely different conditions concerning the environment, concerning the use of antimicrobials, and there is not really any control that we know the consumers are going for the cheapest product and in some cases for chicken, half of the volume of chickens sold is imported. And then of course the domestic industry gets angry because they are saying you are trying to protect your consumers by putting a burden that increases our cost while you let the consumers buy the chicken from [name of non European country].
	Workshop Day 1:<br><br>P: [Name of European country] is the largest, world's largest export of [type of meat], and I mean they have been complaining…you know not only in [name of European country] but in other markets in [name of non European country] and that, they are competed out by products from [name of continent], which are transported half way across the globe and sold cheaper.
	Workshop Day 1:<br><br>P: But maybe adding to this, because I mean this is the European context, and it would be then quite different from Canada, and just takes me back to Monday we had that discussion about guidelines of the US weren't there, but we had discussion about the value of guidelines, and we were talking about the Swedish national guidelines for common infections, whereas I wanted to speak with my Swedish colleague about the European guidelines, but it turned out that she wasn't familiar with the European guidelines really at all because [name of countries] has very strong national guidelines. However in countries like [other European countries] where they do not have very strong national networks, guidelines, mechanisms to tackle AMR, the European guidelines play a much bigger role, and so does the exchange of best practices. So does making funding and resources available. So I think that for the European context at the political level is extremely important, and it is different from [North America].

	P: Just another comment looking at similar interventions, we were looking at mainly internals pressures, or pressures from within the European union, but there is also the external pressures coming from international trade, international agreements. What about climate agreements. We move into security agreements, international organization regulations is one. So there is a whole group of different, domestic type pressures, to international pressures all coming together to drive the political agenda in a certain way and they might not always be moving in the same direction. That can be a challenge.<br><br>F: So just listening to what you are saying there, should this just be international agreements.<br><br>P: I think it is more than agreements.<br><br>F: Okay.<br><br>P: I think there is also international agreements is more a formal group.<br><br>F: Okay.<br><br>P: But there is also I think informal agreements between countries and ….<br><br>F: Can I just say formal, informal.<br><br>P: Formal and informal I guess.<br><br>F: And I guess my question was more about the trade piece, because you mention like the climate change agreements, which I think are, there may be a trade aspect, but it is beyond only trade. Is that fair?<br><br>P: Yes.
Differing interpretations or implementation of agreements, standards and regulations between countries	Workshop Day 2:<br><br>P: You are talking about insects. One quite interesting thing, I think, is that it is not legal to eat insects in Europe yet, but for instance in [name of European country] they have printed the legislation that it is legal. So I think you see this, but I think that it will be legal in five or ten years or something, but development hasn't come that far yet.

	P: It is reading the legislation like a different, sorry …<br><br>P: No, go ahead.<br><br>P: Because I am talking about like insects for example, like [name of European countries]. They started to actually like right away, but it is like they are reading the same documents and they consider it in another way, like maybe we could go around it like that, and then it was fresh insects and it is milder and it is going to be like you know, then you could reproduce it.<br><br>F: An interpretation piece.<br><br>P: Exactly. [group]
	Workshop Day 2:<br><br>P: Maybe I can add also, because it is linked to that other part of course, that we are not allowed to tail off pigs in [name of European country].<br>And we are actually not allowed to tail off in the whole of Europe. It is prohibited, but it is almost only in [name of European countries] that it is following this directive.<br>Other countries they tail doc 27-28%, because it says in the directive that if it is not possible, then you can, and they have to have enrichment in the pens. Not to tail bite, to prevent tail biting by straw and everything. You can do a lot of things to prevent tail biting, and they put in a chain and it works well for two or three days and then they start tail bite anyway, and then say we have tried, and it didn't succeed, so they we can tail doc. So I don't like that at all…. So, but in [name of European country] you can't do that, because if you really, really try to prevent tail biting and they still tail bite, then you are, even though not allowed to tail doc, because then you have to do other things. You have to look into the feed composition and everything to change something else. So we have a completely different view on that…It is interesting, because it is the same legislation actually.<br><br>F: It sounds like different interpretations in legislation.<br><br>P: That is like the insect thing. That is exactly the same thing. [Countries] just read it in a different way.<br><br>P: Yea.

	Workshop Day 1:<br><br>P: people need to access that kind of information. For example, we talk about international standards and CODEX has developed right now some kind of a guidelines document about how surveillance should be done in [foods?].., but hopefully every country that belongs to the CODEX networks should, for example should start applying, so this is important that this is, this comes to the surface of anybody can access this type of data to see how we are… measure it.
	Workshop Day 2:<br><br>P: So… If you take that point around international trade agreements, Codex Alimentarius that was its name that great code of practice and there is a code of practice for minimize and contain antimicrobial resistance and I just wonder why doesn't that have an impact, because we use that, in the retailer's form, but so there are recommendations, clear recommendation, international recommendation how to minimize and that is about veterinary prescription, etc. no growth promoting, etc. There is a code of practice, but I don't know if it has an effect, but we tried at least in [name of European country] to re-establish, to practice that.<br><br>…F: So you said that the Codex, it is a regulation for people to follow, but people aren't following it.<br><br>P: It is a, it's a code of practice.<br><br>P: It is voluntary more than it is a best practice.<br><br>F: Okay. So it is just choice and there is something there that people aren't following.<br><br>[P: yea]

OVERARCHING FACTOR: Leadership	
**Sub-theme/Sub-Topic**	**RELEVANT DE-IDENTIFIED WORKSHOP QUOTES**
Who are leaders	Workshop Day 1:<br><br>P: It is more than individuals. Maybe it is an organization, voluntary organizations that come together or set out to push the particular …<br><br>F: But what if you have people that are pushing what we perceive as the wrong agenda, because I think that is sort of the vaccine….<br><br>P: There is lots of that too. There is lots of that out there as well you know at the moment, but that is where I think the politicians have to be able to decide, based on scientific evidence, solid scientific evidence. We are back to this issue again. Fake news versus non-fake news.<br><br>P: So the scientific evidence versus other.<br><br>P: Yea but you have all these consumer organizations. You have patient organizations for different conditions. You have patient organizations, I think all these …<br><br>P: But the individual champions like you said, they can be more effective, because they have the exposure and people recognize them. Whereas organizational society, most people don't really…<br><br>P: Yea, I don't, I don't I think it is entirely clear where this leadership comes from. I mean you know it is quite clear that you know wherever you have a leadership vacuum at the moment, in many aspects in the west. I mean I actually find it depressing, that the leader of climate change is like a fourteen year old girl. Right. I mean it shouldn't be. It shouldn't be that. You know…<br><br>P: Definitely.<br><br>P: What, so I think it's, it's very difficult actually to point to where the leadership should come from in this.<br><br>P: It could be as a result of social movements as well and these are quite loose, and, organic, so some of it will definitely be, we can't predict, because these things move so quickly.

Trust in Leadership	Workshop Day 2:<br><br>P: the leadership… the impact of leadership in every context, that who really believe on things that this person is going to be a healthy person, even if it takes ten years or about ten years and have the connection, so the trust between the patient, between the kid, between the parent and between the society member and the society is very important…low [trust] infections, and [trust] gets more healthy people and that is very connected to the authority.<br><br>P: That is what you believe that if you are, if you think about the nurses, doctors, who believe in these patients, on these patients that this woman is going to be healthy. Everything is going to be so, and this person trusts totally, and you have that direction. You have a close relation and you get a warm contact between the people and that gets warmer body, you are not afraid. You are calm. You are trustful and you know where you are going. They both know where they are going. So even if they have antibiotic or not, they, it is very important for them.<br><br>F: So quality of relationship between provider and the care provider and the person, like a person using an antibiotic?<br><br>P: Yes.<br><br>F: The quality of the interaction.<br><br>P: In how the perspective if we think about the goal for the world we don't have, we have a lot of, we have to solve that problem…[The current goal] is not so much trust [in]… leadership. It is not so much trust to give a beautiful world [in] about twenty years…it is not prevention. It is the opposite, [and] people get scared. They get scared. Scaredness [makes people sick].<br>So we have the leadership. The leadership is very important in this prevention of… of to reduce the needing of antibiotics.<br><br>F: So that, and I think so, I think what you are talking about…it is about building those trustful relationships [P: in everything]. Yea.<br><br>P: In everything. In family, in society, in company, in organization, in the world between people who thought everything is between them, and the responsibility for the authority to have the goal that you are

	going to be healthy, no problem, and that the other person connected to that, and this is what you talk about when you talk about medical treatment is… the trust. [Trying to find the translated word] Everything is about relations between people, the feelings, feeling of trust, and feeling of a goal where we are going and that we are going to be healthier next year in body.<br><br>F: So it is a collective consciousness and there is a paradigm shift towards collective health and wellbeing from leadership and, whoever the leader is and that helps set the tone and helps shift how people interact with one another and that changes our choices?<br><br>P: So a cold leader, the family members also cold, [but if they] are together, get warmer. So that is very important prevention to get the leadership even if it is a nurse, a mother or President to make them warmer and that goal where you can really …<br><br>F: I put consciousness.<br><br>P: Yes.<br><br>F: It is a way of thinking.<br><br>P: It is a feeling.<br><br>F: Of leadership. What are you modelling?<br><br>P: Yes. I feel that this is something you have to have…always.
	P: ….I think ….that we don't have any goal for [name of European country]. We don't have any goal for the hospital care system. We don't, we have to get rid of this problem we have, and reduce the money flowing out you know, which they are all the time they are flowing out.
	Workshop Day 2:<br><br>P: Yea, and talking about that, I don't know if you follow the [name of] case? You heard about that? …And…Donald Trump, he tried to convince the Swedish Prime Minister… that they should do…<br><br>P: Send in the rapper. [Laughter]

	P: Yea, the …Prime Minister, said, we can't do anything about this, because the… [another P: judicial system]… is completely separated and we have no corruption. We have nothing like, I shouldn't say that, because I know that there are, but it is so minor and it shouldn't be anything. So yea we have a very trustable system. Yea.
Forward-Thinking Leadership	Workshop Day 2:<br><br>P: To add to that [re: Sweden taking care of its people], I often say because I get this question also, why did you end up there, and I say, I work for twenty years, but also other things, and among the other things, one simple way of putting it is we have no wars in Sweden for…at least for two hundred years [has given us time] to worry about the future, and to put energy…and financial resources into what is coming, and so we could start working on AMR very early. That is one way of, we are a great society without any other problems.
	Workshop Day 2:<br><br>P: [Name of European country] is one of the extreme positive examples of the world. We started long ago. We started like in the early nineties. On the vet side even in the eighties, and not only because of that, but probably partly because of that, we had today an extremely good situation when it comes to resistance, and you can compare it for different bacteria, resistance to different antibiotics, and there is someone now, actually during 2019 who has tried to boil down all the resistance is to all bacteria into one score, to simplify it, and then [name of European country] comes out on top, [name of non European region] comes out in the bottom….<br><br>…So [name of European countries] are low in antibiotic use…at least for human use. They have an enormous veterinary system, because they have much more animals, and what more, there was one thing. Um… nope, okay that is enough. We are living in a different world than most other places actually yes. So we have also a lot to teach and a lot to show, and a lot to share. I think that is probably why you are here also.
	Workshop Day 2:<br><br>P: Okay, but what happens next. What happens when we don't have any more resistance? What is that world going to be like? I mean I think that is really, and this is what I really learned in working with you [P's name], that is super important, because once we oust a regime that we don't want, because we have been demonstrating. What happens then? The risk is that we get back into the same loop.

Political Leadership and factors influencing decision-making	Workshop Day 1:<br><br>P: The political level of course is the trigger. [The political level] needs to be convinced before we have any regulatory change and for sure regulation is a major determining factor for antimicrobial use in both animals and humans.
	Workshop Day 1:<br><br>P: For me it is a big issue, which you already mentioned the evidence, and this whole debate, and in other issues… the difference between science and opinions is somehow been done you know. Politicians, they react when, [inaudible]….not always because there is scientific evidence behind a decision but because they are pressed you know. Who press the politicians? Of course the economy. Of course NGOs, activists. Things have changed, and the agenda is now much more political than technical. This is only one side. On the other hand, I think that there is no real debate. There is much more confrontational ideas than debate. So this sense is quite difficult to get, to reach a possible solution in the sense that we when we tackle the issue each one of us, we want to have our say, before the others, and it is very, very difficult. Would you agree with me …? Is this a crisis, as we are in an economic and social crisis that we are in now, decisions and processes are taking much more … national interest than the common interest…So the whole political debate in Europe is based on tensions, confrontation, more than the balance and debate you know? And in this sense, I want to come back to my main idea, politicians have to regulate, to put in place the legislation in these cases, but the agenda [being] so directed by, or influenced by, some stakeholders that maybe that the relationship between [scientific] evidence and measures is not that clear.
	Workshop Day 2:<br><br>P: Bad leadership. Like misusing power or money or I mean it could be like the industry. I mean being behind this kind of like driving public opinion as well, because I mean again back to an example of energy sector. What the big companies are doing in the energy sector, with the political leadership, and that we have lots of good like you know kind of like that solutions to change and transit our energy system to global you know, grid which is like based on renewables and cleaner and so on. We don't do that, because like the five biggest companies in the world are energy related. All of that has like stuff on the market, and they have like taken like a large pampering investment and that is what I mean, they put like a person in the White House. The first thing that he does is like going out of like the Paris agreement for example…like, 'no I

	am not going to transit like my energy system'… So it could be an example that later could come to that misuse of power and money and the public opinion for changing things.
	Workshop Day 1:<br><br>P: So it is really something that can't, this is again – consumer demand - can be very big if society does not consider something acceptable. Then the politicians will be up to, to include some measuring place.
	F: Sure and just coming back to your example, so then wherever that societal pressure came from, and your example there as a politician then was willing to take a stand and call for the reduction. So is that the need for sort of the, unique individuals that are convinced that are leadership roles. I don't know how to …<br><br>P: It is more than individuals. Maybe it is an organization, voluntary organizations that come together or set out to push the particular …<br><br>F: But what if you have people that are pushing what we perceive as the wrong agenda, because I think that is sort of the vaccine….<br><br>P: There is lots of that too. There is lots of that out there as well you know at the moment, but that is where I think the politicians have to be able to decide, based on scientific evidence, solid scientific evidence. We are back to this issue again. Fake news versus non-fake news.<br><br>P: So the scientific evidence versus other.<br><br>P: Yea but you have all these consumer organizations. You have patient organizations for different conditions. You have patient organizations, I think all these …<br><br>P: But the individual champions like you said, they can be more effective, because they have the exposure and people recognize them. Whereas organizational society, most people don't really…<br><br>P: Yea, I don't, I don't I think it is entirely clear where this leadership comes from. I mean you know it is quite clear that you know wherever you have a leadership vacuum at the moment, in many aspects in the west. I mean I actually find it depressing, that the leader of climate change is like a fourteen year old girl. Right. I mean it shouldn't be. It shouldn't be that. You know…<br><br>P: Definitely.

	P: What, so I think it's, it's very difficult actually to point to where the leadership should come from in this.<br><br>P: It could be as a result of social movements as well and these are quite loose, and, organic, so some of it will definitely be, we can't predict, because these things move so quickly.

**OVERARCHING FACTOR: Media**	
**Sub-theme/Sub-Topic**	**RELEVANT DE-IDENTIFIED WORKSHOP QUOTES**
Media as influencing AMR-relevant issues	Workshop Day 1:<br><br>P: So it is really something that can't, this is again – consumer demand - can be very big if society does not consider something acceptable. Then the politicians will be up to, to include some measuring place.<br><br>F: And how…how what, what are some of those triggers to that shift, because I think sometimes …<br><br>P: Some of those triggers can be for example, the, the making the transparency, increasing transparency, making data by the book available to general members of the society, and so that they are aware of what a situation is, and then [inaudible] and I am guessing about what is happening in the [name of European country] with the [acronym]…. That is what triggered the decision of the Minister to say, okay now we will implement targets of use and I want to see this done by a year two or year three, and I want 75% reduction in the use of antimicrobial in farm production….That was all driven by newspapers showing data.<br><br>F: So media.<br><br>P: Survival pressure through the media
	Workshop Day 1:<br><br>P: The trouble is certainly in the [area of Europe], we have this issue with sepsis that is driving the media, and is really making prescribers very nervous around antibiotics and prescribing, because the threat of a public case of sepsis and a child dies is seem to be greater than the greater risk in giving the antibiotic. So yea we have some conflict currently with other medical conditions.

	Workshop Day 2:<br><br>P: Yea, I would add on to the issue of [inaudible] and awareness and what you mentioned before, media. Maybe I didn't see this clearly, where is the role of press media as a sort of amplifying factor in this, and I'm also thinking of like how, not necessarily only the formal leadership can use media or how media can do things. And, I don't remember when this was, but in [name of European country] it was [inaudible] or some other ..<br><br>P: It was the start of all this business about taking away the antimicrobials in feed and it….wasn't pressure coming…from the consumers really….[but] I would say [it came from the] media [which] is of course is very important.
	Workshop Day 2:<br><br>P: That more I was thinking also like, do you remember when this was a few years ago in Sweden, several years ago there was a campaign for awareness about organic food, like which was the top most, top five most important organic foods that you should be buying. It was coffee, bananas, bla, bla, bla. Although it was fair trade, I think. I don't remember exactly, but at least it made like a lot of people talking about this and they even made it into a competition. Oh look at [name of European country]. They are so aware, we should be better, and I think, I don't know, but I think this had actually had five things, that I heard something about them following up on that. We could use, we could use to amplify the media in so many more ways.
	Workshop Day 2:<br><br>P: Yea. I don't think the media don't necessarily always know that they are part of amplifying [inaudible]. I mean that is what you mentioning, the awareness, and the contenders, [inaudible] as well.<br><br>P: We had the shame of halloumi. [Group laughed]<br><br>P: That's a good example.<br><br>F: The shame of halloumi?<br><br>P: Yea, it's… I mean a lot of people in Sweden are moving to vegetarian [inaudible] and so the media think we [inaudible] regardless should not choose halloumi, because it is related to antibiotics.

	P: That is one of the problems when we believe, when we are advised to use media as a clever way of rising kind of… thing here, because a lot of the… when you read about it, AMR, the data stuff in the media is more, actually it is a lot of, it is scares us and a lot of [inaudible] about halloumi case, really, not really proper science based kind of stuff. There is a big risk, [inaudible] the media didn't help us very much here.
	Workshop Day 2:<br><br>F: …When we, when we were mentioning press and media, did we mean sort of traditional newspaper, television or more the social media?<br><br>P: Social influencers as well. That is [not] like …very much academic, but they influence people. They more than like you know normal scientific articles, you know.<br><br>P: It belongs on Instagram pages like the biggest media, normally that we have.
	Workshop Day 1:<br><br>P: Fake news versus non-fake news.
	P: Because one thing behind the media business, which is quite interesting is how they are using in like data science and artificial intelligence behind media as well, because you could drive like public opinion quite easy by using some words, and this is something that a machine could analyze for us. Like, if you are putting your sentence like that, people would be like more like this. If you format exactly the same thing a bit more like that, then people consider it like that. So it is very important, like how you could actually like influence public opinion, through social media that is easy.
	Workshop Day 2:<br><br>P: In some countries it is more difficult, b….because I generally believe that the Swedish society is quite [inaudible] educated society, and that is why I mean most of this kind of like demographic situations, and public opinion and things like that work, but in many other places when you have like this mass media going in one direction, like then democracy puts [inaudible] work with the bureaucracy and that sense like putting like really wrong people in wrong places. I guess we have a lot of examples of that now.

OVERARCHING FACTOR: Collaboration	
**Sub-theme/Sub-Topic**	**RELEVANT DE-IDENTIFIED WORKSHOP QUOTES**
Benchmarking, cultivating trust and collaborating to share information	Workshop Day 1:<br><br>P: And also benchmarking then a little bit like a lot of our work is to kind of, we are not pointing fingers at anybody, but we like to point out okay well these countries have done that and it works well in their specific context. Perhaps you can learn something from that. Some elements might apply in your context as well, but we realize you need support, and that is where Europe then comes back in as well, but unfortunately that is what happens.
	Workshop Day 1:<br><br>P: [examining] the impact of adapting to the new AMR um…needs, you know what I mean? Because there is a clear and direct effect in terms of costs and economics in general for producers, the industry, veterinary industry, change attitudes, those kind of things – this is also very important.<br><br>F: So you're thinking about the new action plans and all the agreements that are at play.<br><br>P: Exactly…
	Workshop Day 1:<br><br>P: Then come back to this idea of benchmarking and exchanging positive or negative initiatives, one of the major points on this one health initiative …. is to make this benchmark, to cooperate, to make comparisons between what we are doing in different countries. So there is also another very strong comment from me, which is that this political initiative for the European Union, is information cooperation. So they have not only talking about in Europe. Also how can we contribute to the rest of the world in order to … [inaudible] the problems, we have on top of the table you know, which is a very European thing. Impossible I guess, I think.<br><br>F: Okay. Like you mentioned the benchmarking. Is there something that we need to think about with that sort of that comparison piece, because that is the idea with the benchmarking isn't it, is that you can compare different countries back to an original time. Is that something that we need to capture?<br><br>P: Yes, I think so. I am not sure where it fits in the whole picture, but we are missing, I thing about the …. Fact finding, the surveillance, the reporting, the pictures of where we are in all these little nodes, because

	people need to access that kind of information. For example, we talk about international standards and CODEX has developed right now some kind of a guidelines document about how surveillance should be done in [foods?].., but hopefully every country that belongs to the CODEX networks should, for example should start applying, so this is important that this is, this comes to the surface of anybody can access this type of data to see how we are… measure it.
	Workshop Day 1:<br><br>P: I think one thing that is helping on this front now is the less secrecy today compared to ten years ago on that. Ten years ago a lot of countries didn't want to even talk about AMR issues [inaudible] Today we see much more participation, much more transparency and this in itself is a positive step. This will lead to better gathering of data, better sharing of data, which will in turn lead to better prioritization of policies and also allow us more budget around the whole system and within the system yea for each species and I think it is all this systematic approach and it will take a lot of time.<br><br>P: That is definitely the case for humans where there is not economic, you know, it is, it's much more transparent in the human system. When you start talking about animal systems, you know and there is economic disincentives for being transparent and it still is. It is nowhere near as much transparency. I mean yea, there are all sorts of disincentive for sharing information in where you are farming and so on.<br><br>F: If this is part of, like coming back to your point [name of participant], if like, if this is happening to some extent, maybe more on the human side than the agriculture side, um, what is behind that? Is that like, are people more willing to trust? Right you need to build …<br><br>P: You need to build the trust and I think there is less trust on the agricultural side up to now. I think it is improving and, and I think it is also a cultural issue than the lack of sharing as I see it as a competitor if that makes sense, because the big difference between antimicrobial or antibiotic use in humans and animals is to have a bigger economic impact on production and productivity in agriculture, just coming to the human side. Human side would want to mainly cure people or treat them for a particular infection, so they can live longer and, something like that, but on animal side, you are influencing their productivity, but again we growth promotion and often with the use of antibiotics for preventative medicine. So it can make the difference between as I said earlier, the profit margin, whether it is in the black line or the red line.<br><br>P: And the big producing animal producing countries like [name of countries]. [Name of European country] said that on the [type of animal] side, and I think they have, I think it is 80% of antibiotics in animals…go to

	[type of animal]. So they, it is a kind of a many to some extent a production secret of other producers and they are competing on domestic market, and the international markets.<br><br>P: … I mean there are reasons for confidentiality when you are farming, where you don't share …<br><br>F: But you have transparency of data and maintain confidentiality, ideally.<br><br>P: Yes.<br><br>P: It is possible. Depends on the granularity of your data.<br><br>P: So, I mean that can be the difference between the human side of things, where we can have as much transparency as needed and then there are, there are practical sides to why there is not quite so much transparency. I mean you can set to level or you know some, you know, some smaller geographical levels as well have a certain amount of transparency, um, but it does, I think there are issues.<br><br>P: We often do not think of farming as a business. We think of farming as something there that is nice and cozy and everything is good and they produce organized food in a nice way and we are all healthy and happy, but farming is a business. It is like a pharmaceutical business. It is like any business. They are in the business of making money and sometimes we forget that and in a business you have business secrets.<br><br>P: Yea.
	Workshop Day 2:<br><br>P: What we just need to understand that when we talk about this, this consensus tripod culture in [name of European country], and everyone talked and try to find good common solutions together.
	Workshop Day 2:<br><br>P: Yea, no it is regarding non-competing areas. We have a tradition of collaboration, so it is okay. We are on the right side of the world, and so I think that's maybe it is more of a solution. I think for example there is one regarding antibiotics

**OVERARCHING FACTOR: Climate Change**	
**Sub-theme/Sub-Topic**	**RELEVANT DE-IDENTIFIED WORKSHOP QUOTES**
Climatic temperature Changes and AMR	Workshop Day 1:<br><br>P: I think this brings us in to the area we haven't talked much about is the environment, and climate change in relation to AMR. I think there are a group of things there…..what is the correlation? Warmer climate versus colder climate. I think there is some evidence that in cooler climate. I won't say cold, but colder climate there is less risk of AMR depending on what resistance you are looking at, than in the tropical climates. There is some evidence of that from a limited number of cities. So perhaps that is something we need to think about a little bit as well, the climatic conditions.<br><br>F: Is that climate change situation or is that a temperate versus tropical.<br><br>P: No, I think it is climate change. I mean I have linked climate change here to food security, and the retail cost of food, and then link that back into social inequalities, because it is the people on the margins of society that will be most affected by climate change, and bringing that in, but so it fits into a number of areas. I mean it does fit into the water stuff because of flooding and sewage than on open farmland and water courses and all that sort of thing. So it is interlinked in many ways.
	Workshop Day 1:<br><br>P: and another good example would be where climate change is having a direct impact on AMR is in for example salmon farming, where the season is now two degrees warmer, [and] the second you get even a degree warmer, you get more disease outbreaks, because the animals are more stressed, so climate change needs to be linked into animal welfare stress, farming practices, and so on. So not only do you get more disease outbreaks that need treating, full stop, but you get new diseases coming in as well. So we see that, so we see new diseases, we see more outbreaks, with more disease ….
Climate change and research on AMR in aquaculture and the natural environment	Workshop Day 1:<br><br>P: I mean you know we know actually nothing about really what is going on in the natural environment, largely because much of that research is just not been funded. You know, funded, we are starting to get some more funding in the [area of Europe] for that kind of thing, but you know even now it is very difficult to get funding for antimicrobial research in aquaculture, because it is perceived to be a much lower risk than terrestrial livestock species. Right and then it is another step down for the environment, but it is slowly changing.

	F: But does the climate change sort of shift that a bit?<br><br>P: Climate change certainly shifts it,
Sustainable Development Goals, climate change	Workshop Day 2:<br><br>P: We also talked about how that [AMR and ensuring healthy people and healthy animals] actually could be like this very focused case for implementing like all the SDGs more or less.
	Workshop Day 2:<br><br>P: The thing with SDGs is I mean in some countries it is sort of like a very accepted fact that I mean it is kind of like a bible that everybody should follow. It is like you know the approach was like sustainability, the climate crisis, global warming. In some other countries people don't have or even maybe like the political leadership doesn't have any belief or any kind of like even like relations to SDGs as well…, and then you realize when you go outside this bubble of like everybody believing in SDGs… It is kind of like a fact …in [name of European country] everybody agrees that SDGs we should follow them…sustainability is really important, the climate issue is super important, but probably that is not the case for many other countries.
