# Supplementary material for: Factors influencing antimicrobial resistance in the European food system and potential leverage points for intervention: A participatory, One Health study
Source: PLoS One. 2022 Feb 22;17(2):e0263914. doi: 10.1371/journal.pone.0263914 (PMC8863257; doi:10.1371/journal.pone.0263914)
Supplement: S3 Table — (PDF) [file pone.0263914.s003.pdf]

S3: Quotes per leverage point

Please note:
Quotes per row often contain the conversation of multiple participants. In keeping with the University of Waterloo Ethics requirements, we have labelled each contribution per participant as “P” and for facilitators as “F”.

**Leverage Point: Multi-pronged approach**	
**Sub-theme/Sub-Topic**	**RELEVANT DE-IDENTIFIED WORKSHOP QUOTES**
Multi-pronged approach	Workshop Day 1:<br><br>P: I think as we said throughout the whole day, everything is a multi-factor approach.

**Leverage Point: National budgets, money and funding (causal loop diagram factor)**	
**Sub-theme/Sub-Topic**	**RELEVANT DE-IDENTIFIED WORKSHOP QUOTES**
Investing in universal health care	Workshop Day 1:<br><br>P: Yes, we had fourth one. More than a human area, which was maintaining or sustainability of the national health and assistance in general and, and it is what we mentioned before that this seems to be very much linked to the European thinking of universal health care and how do we find ways of ensuring that the quality of what is offered is maintained and not restricted and the steps not having to knock on heads on wrong decisions by politicians.<br><br>F: And is there any specific action to do that?<br><br>P: Money, I guess?<br><br>[Group laughed]<br><br>F: And where does that come from?

	P: I think the underlying I guess the white elephant that we never talk about is the budget process. And, and whenever you try to get some kind of a budget on AMR implementation or AMR targets, nobody is willing to actually give you any indication of the budget.<br><br>P: Yea.<br><br>P: Where it is actually incorporated within the general disease control systems, whether it is educational, or whether it is a new budget. Very, very difficult to get some figures on that thing.<br><br>F: Okay. Figures on a cost or figures on like ….<br><br>P: Both I would say.<br><br>P: Yes, the cost of doing it, and then the benefit. You know, yea maybe we are at a too early stage. I am not sure, but I think that would help a lot, [P: definitely], actually get some indicator figures. Not exactly precise but started on that, but it would be a better idea of what the actual costs of AMR is in different sectors, different stakeholders, even rubbish ones. At least something to work with, because there's not, you are right, I'd say it is much more difficult to leverage money when you don't know how much, how much benefit there is.<br><br>F: So, can that be one of those interventions, one of those action points?If we had that information on this?<br><br>P: Yea.
Investing in health promotion and prevention agenda, including good farm practices	P: It is physical and mental health actually.<br><br>P: Absolutely.<br><br>F: So improved physical and mental health well-being.<br><br>P: Yea.<br><br>P: That is easy.<br><br>[Group laughter]

	P: Let’s say the Finnish presidency right now is talking about what the economy of well-being is their catch phrase, so there are, there is thinking around this, but we don’t know yet how to get there exactly.<br><br>F: And if this, like if we want to, if we want to do this, are their specific things that we should do now?<br><br>P: Increase the prevention budgets?<br><br>P: So we talked about increasing the prevention agenda. Ah, it is quite, it’s quite again it is quite an intangible thing. So I often talk about how do you operationalize public health, because we talk about hospitals in terms of delivery. Um, we could quantify many ways hospital based care or interventions or whatever. Public health is different, we know we don’t have the same sort of thing. It is far more woolly isn’t it. So and we have had conversations about this with AMR how we operationalize public health and I don’t have the answer, but, um, it is almost like it is always the poor relative of health care, but health care can only ever capture the outcomes of our public health system, so it is almost the wrong way around, you know. All the attention goes on hospitals and health care.<br><br>P: So that is easy. [Group laughter]<br><br>P: Yea, that’s definitely a problem.
	Workshop Day 1:<br><br>P:…And I think I mentioned… that some of the countries in Europe where there are huge problems with antimicrobial resistance on the human side... if we look at the results of…point prevalence surveys…these countries in some of the hospitals, they don’t have any infection control nurses or any infection control staff at all. So, the investment in that has a result, or has an effect if you don’t invest. So it has a cost to…You would need to establish a continuous education of staff but once you decide you want it, it costs forever. You cannot decide to train one set of infection control nurses and hope they tell the next ones. I don’t think that works.
	P: Okay so we have four main areas. One is around quality of use, so human, animal, … our own use, and we identified options there that could either be restrictions through regulation a set of targets or things like that, or working around economic incentives to try to promote good practices. So this is the first …<br><br>F: We had economic incentives.

	P: Yea or, or disincentives, or use of regulations, restrictions through regulations so those could be the main actions to meet those.<br><br>F: And those would both be interventions for use.<br><br>P: Yea for use.<br><br>F: And that would be both on the agriculture side and human side?<br><br>P: Yes.
Providing insurance to farmers	Workshop Day 2:<br><br>P: …the farming industry… if you don't know the situation of MRSA, you don't know what to do really, and they don't want to do the screening, because that means that some…will…have a problem - who is paying…It is the economy. Of course if you did screening…you would find MRSA in one pit. It would have catastrophic economic consequences to that farmer.<br><br>P: That depends on how you handle it.<br><br>P: But I mean that is the responsibility of the system or dimension to put that in place, to [provide] the economic insur[ance] for that kind of farmer.
Incentivize industry to develop new antimicrobials	Workshop Day 1:<br><br>P: we also need to incentivize the pharmaceutical industry to come up with new antimicrobials, because it often takes twenty, thirty years to come up with it. It takes a long, long process, and that can be done. I think, we didn't talk today very much about public, private farming and I am sure you have come across that in many areas, but I know there is quite a bit of work done on looking at incentivizing the innovation system through public-private partnerships, because of the cost involved are humungous.
	Workshop Day 1<br><br>P:…we need to move away from us being paid per dose, we need to delink volume sales from our reimbursement.

	Workshop Day 1<br><br>P: Yea, and I think it is also how pharmaceutical companies get their income, because I think one of the, … in the [area of Europe] there is a system being trialed instead of being paid by let's say a number of units, they are paid or they would be paid by maintaining a certain product in the market. So access to the antibiotic rather than the units that are sold, because otherwise the pharmaceutical companies do not have an interest in maintaining certain operations which are not economically viable. So it is finding new ways of let's say compensating industry for maintaining a non-profit…I am talking about …..developing new systems to pay companies to maintain.

Leverage Point: Retailer Demand for Product (causal loop diagram factor)	
**Sub-theme/Sub-Topic**	**RELEVANT DE-IDENTIFIED WORKSHOP QUOTES**
Retailers develop collective food procurement agreements	**Workshop Day 2:**<br><br>P:…This would be a significant one and that it might create a whole shift in the system in a way that is desirable in terms of reducing resistance and the impact of resistance.<br>We chose these collaborations, which was not on your original map, which can have an impact on sub-suppliers using, not using antibiotics in animals, etc….because we thought that that could have quite a big impact…<br><br>P: Because then that will have an impact in… you know good farm practices probably everything else.<br>Well we were talking there on a European level. I mean obviously we have this already implemented in some way in [name of European country], which I was not aware, but then on a European level, implementing that more retail companies go together and put force on their sub-suppliers. I mean the farmers from where they source their food or supplies.<br><br>F: So increasing collaboration among retailers.<br><br>P: Yea.<br><br>P: Maybe like increasing the requirement level on your suppliers of the system from the farm to everybody who is processing the food and so on and so forth. So that was one thing that we wrote down.

Leverage Point: Agreements, standards and regulations (Overarching Factor)	
**Sub-theme/Sub-Topic**	**RELEVANT DE-IDENTIFIED WORKSHOP QUOTES**
AMU and AMR-relevant regulations	Workshop Day 1:<br><br>P: Okay so we have four main areas. One is around quality of use, so human, animal, … our own use, and we identified options there that could either be restrictions through regulation a set of targets or things like that, or working around economic incentives to try to promote good practices. So this is the first …<br><br>F: We had economic incentives.<br><br>P: Yea or, or disincentives, or use of regulations, restrictions through regulations so those could be the main actions to meet those.<br><br>F: And those would both be interventions for use.<br><br>P: Yea for use.<br><br>F: And that would be both on the agriculture side and human side?<br><br>P: Yes.
	Workshop Day 2:<br><br>P: …If you look at [name of some European countries], it is a totally different place and in [name of European country] all the [type of animal], or at least…the [type of setting] have MRSA, and they have a large problem with farmers, who are taking care of the [type of animal]. When they come to the hospital they have to be isolated, which has created quite a turmoil, and in [name of another European country] they have gone the other way. When there is a [type of animal] with MRSA, they stamp out the whole population… [name of European country] has seen both examples and [has] not decided.<br><br>….I mean [doctors] are today telling MRSA patients what they can do and what they cannot do [because they] have this legal authority….and have… been trying… with the proper authorities to also put into that that if you have MRSA clear wounds or you are a MRSA spreader, you are not allowed to work in a [type of

	setting] for example, but… couldn't get into the legal level…[but] got into the level under where they are advised not to go, etc.,
	Workshop Day 2:<br><br>P: If we talk about new alternatives coming in, they should not be marketed directly to the decision maker. They should then come in through the expert groups at the national level…and should be evaluated there, or [through] a medical products agency.... I see that is a risk to have direct marketing to the decision makers. That is what we have in other places and the results are devastating.
Public procurement	Workshop Day 2:<br><br>P: Then when it goes to like legislation we talk like public procurement could be like quite important to also like put some pressure on some actors or add to like make it easy for the people buying from the private sector actually to make more like you know better alternatives and give like some sort of upper hand to those who provide like antibiotic free or sustainable alternative and so on an so forth, local food which costs more and so on.<br><br>P: The public procurement has a great impact, because of course that is [name of European country], that have free meals for school children. Yea. So then you should have a great impact…<br><br>P: And your healthcare as well<br><br>P: There was some pricing policy but I don't know how we could capture that, because the pricing policy in public procurement is one thing. Pricing of food in general is something else, but then you need to demand quality of the food being produced. So [the proposed public procurement would require that] you can't basically like sell bad food.

**Leverage Point: Psychological Health (e.g., stress, producer mental health) (causal loop diagram factor)**	
**Sub-theme/Sub-Topic**	**RELEVANT DE-IDENTIFIED WORKSHOP QUOTES**
Gathering and sharing data on health promotion and prevention interventions	Workshop Day 1:<br><br>P: … getting to better data, so that we can actually measure this problem and share. You know, where, you know where can we actually measure improvements in it [prevention – i.e., improving physical and mental health and well-being].
	Workshop Day 1:<br>P: I mean interventions for improved physical and mental health well-being. I mean …[laughter] it is not difficult but …<br><br>F: Interventions associated.<br><br>P: Yea, I am not an expert on that right, but there is a lot of interventions that …<br><br>P: There is a lot we already know.<br><br>P: Yea.

Leverage Point: Understanding and awareness of scientific evidence, surveillance, and best practices (causal loop diagram factor)	
**Sub-theme/Sub-Topic**	**RELEVANT DE-IDENTIFIED WORKSHOP QUOTES**
Education and training	Workshop Day 1:<br><br>P: Education of farmers and producers. Because often I hear the description when we are talking about use of antibiotics and antimicrobials in livestock, the focus is always on veterinarians and very seldom you hear actually people talk about maybe we should actually share some of this information with farmers, and I think there is not enough going down to the grass roots level, to the farm level and the informing farmers of the consequences one way or the other.<br><br>F: Okay.<br><br>P: Even though in Europe it is a little bit different because everything leads to a prescription.<br><br>F: But if we are talking about … farm practices, the vet is the small piece of that whole thing.<br><br>P: Absolutely.<br><br>F: So then the producer is more of the expert I think, in crop management and… housing and some of those pieces.<br><br>P: And that comes from research as well into what is a good farm practice.<br><br>F: In Europe… we struggle with this in North America anyway, but when we reach our producers for alternate producer organizations, so chicken farmers in Canada, Ontario chicken farmers, whatever, um…and I think we struggle with sometimes that message gets to the chicken farmers of Canada workers but doesn't actually get to the producers on the farm. Is that a similar challenge in Europe?<br><br>P: I would say yes.<br><br>P: Mostly likely.<br><br>F: Okay.

	P: The associations and the people who are well informed in the associations doesn't necessarily mean they are members. I think it is the same principle for all kinds of associations or organizations…<br><br>F: And with… [name of participant] would that be the same in Public Health as well?<br><br>P: I think so. [Group laughed] Unfortunately.
	Workshop Day 1:<br><br>P:…And I think I mentioned… that some of the countries in Europe where there are huge problems with antimicrobial resistance on the human side... if we look at the results of…point prevalence surveys…these countries in some of the hospitals, they don't have any infection control nurses or any infection control staff at all. So, the investment in that has a result, or has an effect if you don't invest. So it has a cost to…You would need to establish a continuous education of staff but once you decide you want it, it costs forever. You cannot decide to train one set of infection control nurses and hope they tell the next ones. I don't think that works.
	Workshop Day 2:<br><br>P: Increase the rationality of antibiotic use, both in the human sector and in the veterinary sector, and the way to do it is to produce guidelines at the national level, looking at the evidence and then seeing too what they are distributing and taught to everyone, to every user, every prescriber.<br><br>F: And I think that is one of your examples, but the idea that it is, taking what has worked in [name of European country] and trying to translate that into other context.<br><br>P: Yea.
	Workshop Day 2:<br><br>P: And we had a simple one. Education in schools, so we think it was raising the awareness regarding resistance amongst children, both from a human perspective, but also related to good [health and farm practices].
	Workshop Day 1:<br><br>P: Supporting education.

	F: Okay. So is education then one of those interventions or actions?<br><br>P: We talked about education as an action. Didn't we?<br><br>P: Yea.<br><br>F: Okay and education of who?<br><br>P: Oh everyone really. Education really around this subject. The example that we, we had a couple of specific examples, but one of them was around individual versus population benefit using antibiotics so you know people understand it, but yea antibiotic may be, um, beneficial to you or you may see no downside in taking an antibiotic if you've got viral infection or been prescribed that for a viral infection, but educating around you know population level, that the risks associated with that, and so changing in patient expectation. That was just one example, right.<br><br>P: No one thinks of the risks about taking an antibiotic. I think we are educated in the risk, about what taking an antibiotic does. I mean, messes up your gut microflora for a year, right? So about a year once you have a taken an antibiotic course for you to get back to …<br><br>F: You won't feel that or see that necessarily.<br><br>P: Yea, precisely.<br><br>F: I think that's the, now that the perception is that the vaccination of risk, the risk being vaccinated can be higher than the risk of the disease. That is the perception.<br><br>P: Yea.<br><br>F: Whereas I think the risk of not taking the antibiotic are perceived to be higher than taking it.<br>P: Exactly. It is the opposite, basically the opposite yea.
	Workshop Day 2:

	P: We thought that creating some kind of movement regarding antibiotic resistance related to one health and sort of market that as something.<br><br>P: It is actually already marketed thing with one health and one welfare it is also, it is newer wording, but one health marketing to spread that more. I think it is quite spread already, but…<br><br>P: But not publicly.<br><br>P: No, maybe not.<br><br>P: It is still in the small world.<br><br>P: In the scientific world.<br><br>P: A positive movement. So that it is not about pointing fingers to others or, but more as sort of getting in the vote, everybody saying we have to solve this together.<br><br>P: I mean that is a very interesting thing, because I mean you could actually like go with like bigger issues like when you approach like the climate crisis for example as a crisis or you talk about like how you could impact it for more of a positive setting, and then when you talk about like I am doing this like positive movement and do you want [to give] a hand with me… and like then it works quite better than like talking this kind of like upper political like situations and fear and sickness and of course if there is, there is stronger, like for us human beings, which are quite like selfish and have like a big knee and I should protect myself and my children and so on, but in the end, I mean at the end of the day, those kind of like approaches win for like a longer period.<br><br>F: Long term.<br><br>P: Yea.
Sharing country-specific best practices and scientific evidence	Workshop Day 2:<br><br>P: Maybe we can add to your discussion. We had a similar one more like sharing, because what we said that I mean [name of European country] is a very good example of, a good example of how both

	production and regulation can be done and this could of course be shared. So sharing best practice …[that] could also be done in other countries within EU.<br><br>P: Both on humans and veterinarians.
	Workshop Day 2:<br><br>P: Raising knowledge. It is not about exporting the best practices. It is more about exporting the knowledge…science academia knowledge which we have in [name of European country] and export that to other countries as well.
Working with media to translate knowledge effectively	Workshop Day 1:<br><br>P: Isn't that one of the great challenges here that everything was potentially connected and related, and which makes it even more problematic for media to try to, because it is always for the media if there is one thing that to write about and also to come up with suggestions on what is the proper way of, how should we act in order to solve this, but there is no simple solution here, which makes this an enormous challenge.<br><br>F: …. Is there sort of a knowledge translation piece?<br><br>P: Yea. I mean the collaboration is based on the experts and what they think what a person should do.
Advocacy	Workshop Day 1:<br><br>P: I think that was also, so you have to have uh, kind of the lobbying efforts from NGOs to some extent. NGOs that are moving in the right direction. I think positive NGOs in that respect, like positive NGOs and planning change. They have had a big impact on how politicians have better understood the consequences of not doing anything and today I think that is one of the driving factors, even the young colleague here from [name of European country]…I think recently was in the UN was it.<br>and that…is probably a bigger impact on the G seminar, the G20 maybe.

Leverage Point: Consumer demand (causal loop diagram factor)	
**Sub-theme/Sub-Topic**	**RELEVANT DE-IDENTIFIED WORKSHOP QUOTES**
Increasing transparency of AMR-related issues (e.g., AMU, animal welfare) in products	Workshop Day 1:<br><br>P: …Our second leverage point, it is really societal consumer pressure of concerns. We believe that this is the main driver for political precision and weight, and, and we can change. So if you have enough people that need consulting, that …<br><br>F: And demand?<br><br>P: Yea.
	Workshop Day 1:<br><br>P: Have you seen these, this antibiotic footprint, label suggestion that has come out recently, that mostly ties up both of those which is trying to attribute an antibiotic footprint to a product. So how many, how much antibiotic has gone into producing a particular product and putting that on supermarket labels. So at least you are labeled there, which is an antibiotic footprint, which I think is quite a nice idea, that ties into both of these things, because that may create a new market for premium products, because that market may not exist right now, but it also, you know, it also incentivizes reducing your [inaudible] use, but gives the consumer another, another choice.<br><br>F: Okay. So that, so that is an action or intervention that could be associated with this.<br><br>P: Yea. It can, it could be. I mean I don't know if, I don't know if anybody knows if it is going to work or not. You know it is a good idea though.<br><br>P: I guess it increase in transparency you know and [inaudible] to the choice of hospital or the choice of health care provider, if transparent information could be abide for any consumer to say I want to go to this hospital or not, because I can chose to go to this one. That is much healthier, call it whatever you want. So I think it is this increase of transparency and consumer choice or not. Plus all the policy that comes later as a result of what people want.
	P: [Name of country], they are really, really good in marketing and they have started to marketing … for animal welfare.

	R: Okay.<br><br>P: And then [to get] the [labelling for animal welfare]…, you have to produce the meat in the way that is the legal level in [name of European country]. Then you… can add some other values. For instance outdoor and so on, and get more [type of labelling to identify that production practice], but I think that marketing is also a very good way to, way forward to, because one is that you should have a very low antibiotic use, and they have also marketed no antibiotic meat for animals that have had no antibiotics at all, and they get 30% more for the meat in [other non-European and European countries] and it was one more country that they sell to, and we produce like that all the time in [name of European country], but we don't see the values, or we don't market the values.<br><br>F: So is that… sort of an action…?<br><br>P: Yea, definitely.

<table>
<tr><td colspan="2">Leverage Points:
Non-antimicrobial disease prevention and infection control in plant agriculture (e.g., heavy metals).
Non-antimicrobial infection prevention and control by public (e.g., hand hygiene, home cooking; social isolation; access to sick days).
Non-antimicrobial disease prevention and control in health and social care settings (e.g., hospital and long-term care).
Non-antimicrobial infection prevention and control in other social institutional settings (e.g., restaurants, workplace, community, home).
Non-antimicrobial infection control on farms of food producing animals.
(5 causal loop diagram factors)</td></tr>
<tr><td>Sub-theme/Sub-Topic</td><td>RELEVANT DE-IDENTIFIED WORKSHOP QUOTES</td></tr>
<tr><td rowspan="5">Good farm practices (hygiene, infection prevention,</td><td>Workshop Day 1:

P: More education and sharing about good farm practices. Sharing of lessons on successful communities or successful, successful countries like what has happened in the [name of European countries] as well. I mean by successfully in reducing antibiotic use and so the lessons and with very little change in the cost structure. So those are all lessons to be brought and shared with other countries that are in a similar pact, even though then you are assuming that the structure and production are the same which may not always be the case.</td></tr>
<tr><td>Workshop Day 1:

P: Yea, we had another one that was fairly obvious around the good farming practices, and anything that we can do to improve the way we raise the food producing animals and keep them being in healthier conditions. …so that, at least in the agriculture area important for the, and of course you have to revise biosecurity, the increase of the use of vaccination, etc. etc.</td></tr>
<tr><td>Workshop Day 1:

P: …. [ensure] full welfare of animals, exactly.</td></tr>
<tr><td>Workshop Day 2:

P: …prevention …from the human sector… There is room for prevention also in the animal sector of course. First we say prevention of disease that is, that can be achieved by… increased coverage of vaccination programs.</td></tr>
<tr><td>Workshop Day 2:</td></tr>
</table>

	P: And we also talked about, yes level of hygiene in the human health care, which especially if we look at the European level, there is a lot to do, and there is also much more to do in [name of European country] also, but there is always room for improvement, but especially if you look in [area of Europe]. So prevention of disease by gracing the available hygiene in human health care
	Workshop Day 2:<br><br>P: …Prevention of disease would improve hygiene in the animal sector. Can you help us? You are from the animal sector. Is this still way ahead to work?<br><br>P: Well talking about animal hospitals? or the farming industry?<br><br>P: Actually more the farming industry.<br><br>P: Yea, I think, well you can always improve of course, but I think the awareness is quite high in Europe…We talked …about the [type of] case in [name of European country] many years ago….We had, a long time ago we started to, in [name of European country] as soon as it was a [type of bacterial] outbreak on a farm, you had to slaughter the animals and this infected the whole stable and so on, and then put in new animals, and the European, other countries…. today they are really curious about how do you do [it]. How do you manage to do this? So and [name of another European country] is also a very good example. I mean trying to protect the animals from different diseases just slaughter all the animals and disinfect and so yea it is room for improvements to the other countries.<br><br>P: But I think it is very important what you saying that just say we are the best in the world or whatever in [name of European country], because we are not, because there is still a great difference between farms. [P: yea] How they handle antibiotics and animal health, etc. So I think it is very important to always be on the top to say, we can improve. Not lean back and say, we are at the end of the best. So still be the positive example.
	Workshop Day 2:<br><br>P: But I mean that is very interesting because I mean when you are talking about like control climate or isolated environments for farming, that is like I remember our farm for example here in Stockholm that we have exactly the same thing, that you need to change your clothes and you need to take like an air shower and you go through all this like high pressure rooms and so on, but then we have like two other layers of

	security for the farms as well. Like if there is any insect that gets, or any bugs that gets into the farm, then there is like a procedure for biologic pest control that you could actually run, or we have like UV filters that we do, but obviously for animals it is very more difficult than plants, and then taking away the plants from like a wall is very easier than like you know sending all of your animals to the slaughter house. So I mean how you could actually have this kind of like more clinical environments for growing animals or like you know plants and so on, and then how you should have like a plan B, C, D for preventing if something happens…
	Workshop Day 2:<br><br>P: … I mean we cannot build walls [to] have hygiene standards, so that we cannot even touch each other like this, and so the answer to that is to do it in the right circumstances. To do sharp and well evaluated interventions and one very good example of that is how we try to prevent multi-resistant bacteria transmission from human sector to animal sector and vice versa, and if you from [country outside Europe] are looking at how we do it, at least this is an example you should look closer to is how we have gone about it with MRSA. …If you look at [name of European countries], it is a totally different place and in [name of European country] all the [type of animal], or at least…the [type of setting] have MRSA, and they have a large problem with farmers, who are taking care of the [type of animal]. When they come to the hospital they have to be isolated, which has created quite a turmoil, and in [name of European country] they have gone the other way. When there is a [type of animal] with MRSA, they stamp out the whole population… [name of other European country] has seen both examples and [has] not decided.<br><br>….I mean [doctors] are today telling MRSA patients what they can do and what they cannot do [because they] have this legal authority….and have… been trying… with the proper authorities to also put into that that if you have MRSA clear wounds or you are a MRSA spreader, you are not allowed to work in a [type of setting] for example, but… couldn't get into the legal level…[but] got into the level under where they are advised not to go, etc.,<br><br>F: So really it is about having maybe some targeted interventions or targeted recommendations.<br><br>P: Targeted interventions, recommendations based on the knowledge and evidence and scientific facts....
	Workshop day 2:<br><br>F: how are you going to increase the level of hygiene…? …

	P: Well but it is also…technological assisting measures, but this is, I think this is more coming in the [North America], but some of it may pop up here too, and some of it I think will be useful actually…Devices that reminds the personnel to [inaudible].<br><br>F: So the level of hygiene in human practices targeting the health professional providing care. Is it beyond that? Are there other populations?<br><br>P: …specifically the human health care system.

**Leverage Point: Research, Surveillance, Development and Innovation (causal loop diagram factor)**	
**Sub-theme/Sub-Topic**	**RELEVANT DE-IDENTIFIED WORKSHOP QUOTES**
Microbiome and microbiota	Workshop Day 1:<br><br>P: I think those are the biggest headlines. I mean one of the areas that we haven't really talked about, but I think it is going to be fascinating in the future is the microbiome. So it may be in the future that we just take a yoghurt drink once a day and that is our antibiotic you know and it may be that something gets discovered by accident…You know in the future.
Better Measures	Workshop Day 1:<br><br>P: Yea, where, where in the system can we discover and see when you make an intervention here. Where can we measure what intervention is successful or not. At what point can we actually measure stuff. There are multiple places you can measure these things. There are multiple things you can measure, but it is coming up with those that are most indicative of what is going on.<br><br>F: And so I am not sure how to capture that as an intervention, other than thinking more about the system and the connections between?<br><br>P: That ever circular …<br><br>P: Yea, for me it has come out as indicators and metrics. You know if you want to something that isn't quite so circular, what are the key metrics we should be measuring?
	Workshop Day 1:<br><br>P: It is the accuracy of the measurements as well.<br>I mean antimicrobial use sounds easy. We can measure that. I mean that is exactly the point is you know our measurements on these things, the accuracy still remains … there are many details that we don't understand about how many antibiotics are used you know…we don't really know how much [goes] into the environment through residue or waste…
	Workshop Day 1:

Make AMR impacts tangible	make this problem tangible to the public, policy makers, to farmers, to pharmaceutical industry, to everybody to make that, put some number on it and on each of these.
	Workshop Day 1:<br><br>P: Going back to the intangibility with the conversations at the moment in the [area of Europe] around death certification and putting antimicrobial resistance on death certificates either as a direct cause or a contributing cause to someone's death. At the moment, we went through this with MRSA, and that is one of the ways that, because people talk about how many people die, but actually it means nothing unless you are affected, your family is affected by it. That is one of the ways that we could start to quantify that and that would really influence I think the public, because they are already used to the term super bugs or certainly in the English media. It is not the same across all of Europe. Um,<br><br>P: Actually I even think in the [area of Europe] we, most people know what MRSA is, because when that started to crop up.<br><br>P: Death certificates.<br><br>P: Death certificates, yea, on death certificates, you know it is not very long before, it doesn't take many people, oh yea I knew someone that was …<br><br>P: Of course, if you are talking about these additional deaths, then how are those additional deaths going to be captured if not on death certificates.<br><br>P: So this is it. This is another metric we can use. Right?<br><br>P: And then we would know how many people died as a direct result of antimicrobial resistance, and how many people died with a multi resistant organism, and that is really important for directing activity and actions.<br><br>P: Yea because until we capture, so these young women dying of what would have been treatable urinary tract infections, five, ten years ago, you start recording that on death certificates. You have got a whole different ballgame.

Rapid Diagnostics	Workshop Day 1:<br><br>P: So, um, we talked about the development of new rapid diagnostics …to be able, well at the very sort of, it is not basic talk, but is it bacterial, viral or fungal.<br><br>P: Which would be valuable.<br><br>P: yea<br><br>P: It could be for people and animals.<br><br>P: Yea, maybe it could be across the …<br><br>R: Could be which …<br><br>P: People and animals.<br><br>P: We also talked about agriculture as well, because of the spraying of crops and vines and things like that.<br><br>F: So human, animal, plant.
	Workshop Day 1:<br><br>P: For me if I was going to prioritize, it would be diagnostics, that simple diagnostic, for virus vs. bacterial infection, you know that kind of point of care. If you can do that in the spot, you can tell someone, I am not giving you an antibiotic, because you have got a virus, right.
Traceability systems	Workshop Day 2:<br><br>P: I mean traceability is the other thing which like nowadays I mean digitization enables that you could actually trace back the food.<br><br>[P: Yea] Maybe like too expensive for food sector unfortunately, but I mean data points in something that you have, but then you could maybe like trying to go back in the processes in which part of the process those infections happen, because it could be like in warehousing, bulk buying, transportation, production. You know, it's simply like fourteen different actors between the farmer and your plate.

	P: I think I can add to some of this. Of course I mean like you said transparency. I think that is a very important solution, but I do not know if it is part of the system? Block tracing is something …tested for information for example. So it is really an interesting solution to move forward…
Understanding and developing behaviour change interventions	Workshop Day 2:<br>P: we also need to somehow address like the… the human behaviours… understand [the] pressures physicians comes under when…[a parent]… wants …the ciprofloxacin for his kids.
	Workshop Day 2:<br><br>P: We very rarely applying a lot of the preventative measures we know we could regardless whether that is changing our role, our behaviours…
	Workshop Day 2:<br><br>P: yeah. But I can say actual a find thing that would fit here, which would be nice. I think it is targeted interventions, targeting high risk individuals in high risk environments…because it is really not so expensive. It takes some legislation. It takes some analysis, but it has a lot of effect potentially and it is not so expensive actually. …It is a risk to cost kind of like method…[P: cost effective], yea, cost effective, like resource allocation or targeted interventions.

Leverage Point: Collaboration (Overarching factor)	
**Sub-theme/Sub-Topic**	**RELEVANT DE-IDENTIFIED WORKSHOP QUOTES**
Data sharing	Workshop Day 1:<br><br>P: I think one thing that is helping on this front now is the less secrecy today compared to ten years ago on that. Ten years ago a lot of countries didn't want to even talk about AMR issues [inaudible] Today we see much more participation, much more transparency and this in itself is a positive step. This will lead to better gathering of data, better sharing of data, which will in turn lead to better prioritization of policies and also allow us more budget around the whole system and within the system yea for each species and I think it is all this systematic approach and it will take a lot of time.<br><br>P: That is definitely the case for humans where there is not economic, you know, it is, it's much more transparent in the human system. When you start talking about animal systems, you know and there is economic disincentives for being transparent and it still is. It is nowhere near as much transparency. I mean yea, there are all sorts of disincentive for sharing information in where you are farming and so on.<br><br>R: If this is part of, like coming back to your point [name of participant], if like, if this is happening to some extent, maybe more on the human side than the agriculture side, um, what is behind that? Is that like, are people more willing to trust? Right you need to build …<br><br>P: You need to build the trust and I think there is less trust on the agricultural side up to now. I think it is improving and, and I think it is also a cultural issue than the lack of sharing as I see it as a competitor if that makes sense, because the big difference between antimicrobial or antibiotic use in humans and animals is to have a bigger economic impact on production and productivity in agriculture, just coming to the human side. Human side would want to mainly cure people or treat them for a particular infection, so they can live longer and, something like that, but on animal side, you are influencing their productivity, but again we growth promotion and often with the use of antibiotics for preventative medicine. So it can make the difference between as I said earlier, the profit margin, whether it is in the black line or the red line.<br><br>P: And the big producing animal producing countries like [name of non European countries] and to some extent [name of European country] on the [type of animal] side, and I think they have, I think it is 80% of antibiotics in animals in [name of European country] go to [type of animal]. So they, it is a kind of a many

	to some extent a production secret of other producers and they are competing on domestic market, and the international markets.<br><br>P: … I mean there are reasons for confidentiality when you are farming, where you don't share …<br><br>F: But you have transparency of data and maintain confidentiality, ideally.<br><br>P: Yes.<br><br>P: It is possible. Depends on the granularity of your data.<br><br>P: So, I mean that can be the difference between the human side of things, where we can have as much transparency as needed and then there are, there are practical sides to why there is not quite so much transparency. I mean you can set to level or you know some, you know, some smaller geographical levels as well have a certain amount of transparency, um, but it does, I think there are issues.<br><br>P: We often do not think of farming as a business. We think of farming as something there that is nice and cozy and everything is good and they produce organized food in a nice way and we are all healthy and happy, but farming is a business. It is like a pharmaceutical business. It is like any business. They are in the business of making money and sometimes we forget that and in a business you have business secrets.<br><br>P: Yea.
	P: I just have a question, maybe it is off this map, but I mean I think also that what is specific regarding [name of European country] is that we have a tradition of collaboration between competitors.<br><br>R: Is that business competitors?<br><br>P: Yea, no it is regarding non-competing areas. We have a tradition of collaboration, so it is okay. We are on the right side of the world, and so I think that's maybe it is more of a solution. I think for example there is one regarding antibiotics.<br><br>F: Between the different companies.<br><br>P: Yea. [states name of companies]. They are selling the food.

	F: So this is retailers.<br><br>P: …There is co-ops and farmer own organizations that collaborate, because I mean the culture of cooperating as you said, is quite high.<br><br>P: That agreement has been very important, but all we're changing [inaudible] we should claim good claims on how we do antibiotics in animal production and that is what you are revising.<br><br>F: And so, is that what they have done?<br><br>P: Yea. This is specific examples, so [name of organization] has sort of put these actors together and they have united and said, okay we will put these requirements regarding antibiotics on all suppliers, private label, into the world.<br><br>F: So that is for like animal use. Is that right?<br><br>P: Yes. But I think that in general in the collaboration it is a culture, they need the freedom that it's very… comes from our history I would say.<br><br>P: …[name of organization]…are building bridges. [They] make sure that [retailers] meet and put suggestions on the table and then they agree, and the [retailers] own the agreement.
Understanding unintended consequences of actions	Right, because we can see on here that we already do surveillance of animal and human pathogens and the problem, so resistance genes and so on, but there could be points on here where we look at cost and actually it is a financial amount of money that we can measure, there is a metric as to what is going on, and there may be other ways of measuring you know whether the situation is improving or getting worse. The only other point I wanted to make about the system as well we brought up in our discussion was having some understanding of what the intended consequences we make will change it.<br><br>R: Yea.<br><br>P: Because we know there are unintended consequences.<br><br>P: Some will be good and some won't.

	P: It is very difficult to know.<br><br>P: Unless you measure them.<br><br>P: Yea, so you have to measure them. Yea, that is another reason for measuring as well is then you know we maybe can pick up those unintended consequences and you can make the change over here that is great, but actually then down here the … is far worse, and so you know, I don’t know, it applies to all systems, but that’s the, that’s the key.

Leverage Point: Leadership (Overarching factor)	
**Sub-theme/Sub-Topic**	**RELEVANT DE-IDENTIFIED WORKSHOP QUOTES**
Determine goal of system and associated interventions to achieve them	Workshop Day 2:<br><br>P: I feel that we talk about a lot of different details, but we don't talk about which type of future do we want to have in our world and [so] we [can] solve a problem, but we are not talking about how will it look like when we have it, as we want to have it.…and I feel…when we talk about details in this way and that way…a researcher, I saw him for on television one month ago….he said, in the future…perhaps we have to eat human meat and we don't have to be so scared about that…I think this is, it is very important that we, when we talk about this, take responsibility in this room, which future we want to have…inside me and inside you and inside everybody….We have to talk about [what type of future we want]… it is very, very important to take that direction *before* we try to [determine what actions to take]… in first place or second place. I get frustrated when I sit here.<br><br>F: So how do you do this?<br><br>P: Go back to yourself and think, are we going to be happier or not. Do you want that? How are we going to solve, are we going to be healthier enough…?
	Workshop Day 1:<br><br>P: Again you know it depends what is the end goal.
	Workshop Day 1:<br><br>P: And the final outcome is that we want health for all, for people, and this is good for all of us.
	Workshop Day 2:<br><br>P: I mean it is very interesting. I mean in futurism you talk about this concept as comparing as is to a grim scenario and then there are like four different parts of a kind of matrix, when you talk about that, that it is okay. The role of advancement in technology, societal development and philosophical approach towards that, economical situation, that could enable actually these changes or not, and we could learn actually in two different parts of that in history, to see like what kind of effects this kind of like happenings, or like a war, or like an economical crisis and so on had on our situation, and then you as well go and look at this kind of like things that are, you are not waiting for, like thing that is called for like you know the black swans and gray rhinos phenomenon.

	P: So this is kind of what you are saying, but how you read that is super difficult. I mean to kind of a like a screen to dream scenario, or like a couple of different scenarios how things should be, and that is for like a lot of people tend to focus on one area, like okay the technology is taking over. We should kill like all the scientists. It is going to be better future. I mean it is a very like multi-factor situation, but talking about what you are saying with people from different backgrounds could actually like give us some picture like what is the best system that we want. I guess [P's name] had a great example of that, which is like why this kind of like social movements in many countries fail when we are looking for like democracy or country, you just push out the dictator. What happens is worst case scenario, like afterwards, because you don't have like a plan for after the transition and you didn't screen like a grim scenario. It is like when he is out, we solve the problem, but you can't. So that is very important.
	Workshop Day 2:<br><br>P: so what [P's name] is saying I think it is most important to start with what do we want to achieve… and then who knows what kind of invention will come up that we have never even thought about.
	Workshop Day 2:<br><br>P: what I miss is something that you talked about, prevention [inaudible] if we have healthy people, healthy animals, you diminish the demand… hopefully I would say, and I don't think you have it clear enough really that…that you must focus on health.
	Workshop Day 2:<br><br>P: We also talked about how that [AMR and ensuring healthy people and healthy animals] actually could be like this very focused case for implementing like all the SDGs more or less.
Implement Sustainable Development Goals (SDGs)	Workshop Day 2:<br><br>P: I think when it comes to sort of the common goal, we do have the SDGs. I mean a lot of countries have actually agreed to that. It is sort of the overall target that we are aiming for. So we are not starting at zero, so there are some common ground to stand on when it comes to the direction, and I think it's, I agree that it is important that we know where we are going, but I am a person who thinks that it is also important to actually take steps at the same time.<br><br>F: But considering the consequences.

	P: Yea, absolutely.
	Workshop Day 2:<br><br>P: I think you must list the SDGs as a fantastic tool at least to have decided on which way we should go, and even if it is enormous.
	Workshop Day 2:<br><br>P: The thing with SDGs is I mean in some countries it is sort of like a very accepted fact that I mean it is kind of like a bible that everybody should follow. It is like you know the approach was like sustainability, the climate crisis, global warming. In some other countries people don't have or even maybe like the political leadership doesn't have any belief or any kind of like even like relations to SDGs as well…, and then you realize when you go outside this bubble of like everybody believing in SDGs… It is kind of like a fact here in [name of European country] everybody agrees that SDGs we should follow them…sustainability is really important, the climate issue is super important, but probably that is not the case for many other countries.<br><br>And then the other funny thing about the SDGs is, sometimes they are conflicting those goals. So we talk about… infrastructure development and economical growth when you are talking about like lowering down your foot print so it is a lot of like you know conflicting goals. And as well like benchmarks, sometimes it is like too low in many of them, and you talk about like zero hunger or like you know poverty or things like that, which we basically have no idea. So when people try to capture them with indicators, or you talk about like I don't know, equality and issues like that, for example in schools like how many girls could go to school. Obviously like 100% of them in [name of European country], but not in [name of non European country].<br><br>P: So there are like many different like benchmarks that in some places they work and then you need to just build on top of those and in some places you are just way below this level, that goes back to that inequality kind of factor and then they have earned this and like how much fact based are we discussing things. How much optimism we have and how much pessimism we have, which goes back to your point [P's name], because some people say like, alright like the climate crisis obviously we can solve it if we put our efforts into like a carbon absorbing technology that just basically sucks in air and produces carbon backs, but we don't yet need that because there is no customer that pays for that, but if you want to do that we have carbon capture technologies, but nobody does that because there are no customer for that.

	So that is, don’t go like this kind of like funny things with accepting like a global framework as something that everybody should follow, and then how do we basically [sort it out and] like create that.
