# Supplementary material for: Factors influencing antimicrobial resistance in the European food system and potential leverage points for intervention: A participatory, One Health study
Source: PLoS One. 2022 Feb 22;17(2):e0263914. doi: 10.1371/journal.pone.0263914 (PMC8863257; doi:10.1371/journal.pone.0263914)
Supplement: S4 Table — (PDF) [file pone.0263914.s004.pdf]

S8: 91 CLD factors by 8 categories with definitions

CATEGORY THAT FACTORS CLUSTERED AROUND	NAME OF FACTORS INFLUENCING AMR	DEFINITION OF FACTOR	OVERLAPS WITH OTHER CATEGORIES (the factor applies to more than one category)
**Individual Factors:**			
	Consumer choice, demand, and behaviour	Individual level human behaviour, choice, and demand for products and services	
	Consumption of other (non-meat/egg) foods	Human consumption of all non animal-based food products	
	Meat/egg consumption	The amount of animal-based food products consumed by the general population	
	Food and water security (personal, national)	The state of having reliable access to a sufficient quantity of affordable, nutritious food and clean, potable water	Government
	Nutritional quality of diet	The value of the product for the consumer's physical health, growth, development, reproduction and psychological or emotional well-being.	Government
	Diverse experiences, opinions, training, and culture	The varying experiences and backgrounds of the population that affect how they act and make decisions	Public Health, Health Care and Social Care; Agriculture
	Psychological health	A person's ability to think, feel and behave in a manner that enables them to perform effectively in their work environments, their personal lives, and in society at large	Agriculture
	Human illness	Diseases in humans	

CATEGORY THAT FACTORS CLUSTERED AROUND	NAME OF FACTORS INFLUENCING AMR	DEFINITION OF FACTOR	OVERLAPS WITH OTHER CATEGORIES (the factor applies to more than one category)
	Chronic, non-communicable diseases	Chronic illnesses in humans	
	Population vulnerabilities	Groups and communities at a higher risk for poor health because of the barriers they experience to social, economic, political and environmental resources, as well as limitations due to illness or disability.	Government
	Death (Human)	Number of human deaths (includes all reasons for death)	
	Human AM use	Antimicrobials used in humans for all purposes (treatment, prevention, control)	Public Health, Health Care and Social Care
	Use for prevention in humans	Antimicrobial used in non-infected humans to prevent getting an infection	Public Health, Health Care and Social Care
	Use for treatment in humans	Antimicrobial use in humans to treat an infection	Public Health, Health Care and Social Care
	Use for controlling spread of illness in humans	Antimicrobial use in humans to control the spread of an infection and prevent getting an infection from nearby infected human	Public Health, Health Care and Social Care
	Disposal of AMs (e.g. unused, unmetabolized)	The amount of unused or unmetabolized AMs that are disposed of in waste or wastewater, or are excreted by the host	Agriculture; Public Health, Health Care and Social Care; Research, Surveillance, Development, and Innovation

CATEGORY THAT FACTORS CLUSTERED AROUND	NAME OF FACTORS INFLUENCING AMR	DEFINITION OF FACTOR	OVERLAPS WITH OTHER CATEGORIES (the factor applies to more than one category)
			Environment.
	AROs in humans	Resistant organisms in all humans	
	Host microbiome	All organisms living in and on a host	Agriculture Environment
	Human vaccination	The human population which have been vaccinated against common pathogens	
	Non-AM infection prevention and control by the public	All other forms of disease prevention and control done by the public on a day-to-day basis	
	Understanding and awareness	The varying experiences and backgrounds of the population that affect how they act and make decisions	Public Health, Health Care and Social Care; Agriculture
	Companion animal illness	Disease in companion animals (infectious and chronic)	
	AMU in companion animals	Use of antimicrobials in companion animals (e.g. dogs, cats, reptiles, rodents, horses) for all purposes (preventative, control, and treatment)	
	AROs in companion animals	Resistant organisms in all companion animals	
**Public Health, Health Care, and Social Care Factors**			
	Digital health	Access to healthcare through the internet or phone	
	Prescribing, diagnosing, treatment practices (appropriateness)	The practices of a prescriber (physician and veterinarian) in terms of how they diagnose, plan to treat, and prescribe medication	

CATEGORY THAT FACTORS CLUSTERED AROUND	NAME OF FACTORS INFLUENCING AMR	DEFINITION OF FACTOR	OVERLAPS WITH OTHER CATEGORIES (the factor applies to more than one category)
	Non-AM disease prevention and infection control in health and social care settings	All other forms of disease prevention and control done in healthcare and social care settings	
	Non-AM infection prevention and control in other social institutional settings	All other forms of disease prevention and control done in social settings that is regulated and not under the control of the general public	
	Healthcare costs	The actual costs of providing services related to the delivery of health care, including the costs of procedures, therapies, and medications	
	Healthcare resources	The physical, monetary, and time resources the healthcare system has to be able to accomplish their work	Government
	Existing healthcare infrastructure	The current physical infrastructure of a given hospital that has the ability to affect the day-to-day operations	
**Agriculture Factors**			
	Food-producing animal illness	Diseases in animals (incl. poultry, livestock, aquatic animals) raised in agriculture	
	Disease in plant agriculture (crops, horticulture)	Diseases in plants used for agriculture	
	(Terrestrial) On-farm AM use	Use of antimicrobials in terrestrial food-producing animals for all purposes (preventative, control, and treatment)	
	Aquaculture AM use	Use of antimicrobials in aquatic food-producing animals for all purposes (preventative, control, and treatment)	

CATEGORY THAT FACTORS CLUSTERED AROUND	NAME OF FACTORS INFLUENCING AMR	DEFINITION OF FACTOR	OVERLAPS WITH OTHER CATEGORIES (the factor applies to more than one category)
	AM use in plant agriculture	Use of antimicrobials in agricultural plants for all purposes (preventative, control, and treatment)	
	Use for treatment	Antimicrobial used in animals to treat an infection	
	Use for preventive purposes	Antimicrobial used in healthy animals to prevent an infection	
	Treatment post-procedure	Antimicrobials used to prevent infection in an animal after a procedure	
	Use for metaphylaxis/control	Antimicrobial used in animals to control the spread of an infection and prevent getting an infection from nearby infected animal	
	Use for growth promotion	Antimicrobials used to increase animal's ability to gain weight	
	AROs in plant agriculture	Resistant organisms in all plants used for agriculture	
	AROs in food-producing animals	Resistant organisms in all food-producing animals	
	What is being farmed	The food-products being produced on a given farm	
	Production systems	The main operations, processes, and products used by the producer to generate food products	
	Existing farm infrastructure	The current physical infrastructure of a given farm that has the ability to affect the day-to-day operations	
	Restocking with animals/eggs at higher risk for infection	Bringing in of animals or eggs from a different farm which can carry new diseases that can be introduced to the herd	
	Animal density	The number of animals in a given space	
	Good farm practices	A collection of principles to apply for on-farm, resulting in healthy animals, and safe and healthy food and non-food	

CATEGORY THAT FACTORS CLUSTERED AROUND	NAME OF FACTORS INFLUENCING AMR	DEFINITION OF FACTOR	OVERLAPS WITH OTHER CATEGORIES (the factor applies to more than one category)
		agricultural products, while taking into account economic, social and environmental sustainability	
	Animal welfare/stress	How well an animal is coping with the conditions in which it lives both physically and mentally	
	Non-AM disease prevention and control in plant agriculture	All other forms of disease prevention and control done in plant agriculture	
	Non-AM infection control in food-producing animal agriculture	All other forms of disease prevention and control done in the farming of food-producing animals	
	Feed quality	The nutritional composition and quality of the feed	
	Resistance at the abattoir/processor	The amount of resistant organisms and genes at the level of slaughter and food processing	
	Feed efficiency	The amount of food-product produced relative to the amount of food consumed by the animal	
	Time to market weight	Time it takes for an animal to grow from farm to market	Trade
	On-farm production level (e.g. kg, L)	The amount of food product (animal-based and plant-based) that is produced for sale by a given farm	Trade
	Production costs	The costs related to production of food-products	
	Market price per production unit (e.g. kg, L)	Price the producer receives when selling products to the market	Trade
	Producer profitability	The producer's ability to use their resources to generate revenues in excess of their expenses	

CATEGORY THAT FACTORS CLUSTERED AROUND	NAME OF FACTORS INFLUENCING AMR	DEFINITION OF FACTOR	OVERLAPS WITH OTHER CATEGORIES (the factor applies to more than one category)
**Trade Factors**			
	Number of units (e.g. kg, L) set by quota	The number of units that can be produced which is set by the national quota	Agriculture
	Cost per unit (kg, L) set by quota	Cost per unit of animal-based food products set by quota	Agriculture
	Amount of product in the domestic market	The total amount of food products available for sale in the county's domestic market	
	Viability of domestic meat production	The ability of the domestic meat production to survive without imports	Agriculture
	Retail availability of meat/eggs in domestic market	The amount of animal-based food products that are produced to the standards for sale in retail stores	Agriculture
	Retail cost of food	The relative cost of food in retail stores	
	Retailer demand for product	The types and standards of food products which retailers want to stock in their stores	
	Domestic and international trade	All trade of food-products within and between countries	
	Amount of imported product	The total amount of food products available for sale that have been imported from a different country	
	AROs in food products	Resistant organisms in all food products	
	Exposure to AROs in imported products	Exposure to resistant organisms through contact or ingestion of a contaminated imported food product	Individual
**Environment Factors**			
	Wider environment microbiome (water, soil)	All organisms in the environment	

CATEGORY THAT FACTORS CLUSTERED AROUND	NAME OF FACTORS INFLUENCING AMR	DEFINITION OF FACTOR	OVERLAPS WITH OTHER CATEGORIES (the factor applies to more than one category)
	AM use in wildlife	Use of antimicrobials in wildlife animals (e.g. racoons, rodents, coyotes, birds)	
	AROs in wildlife	Resistant organisms in all wildlife animals	
	Resistance in the wider environment	The amount of resistant organisms and genes in the surrounding environment	
	Treatment of waste and waste-waster	The treatment of waste (human and animal) and wastewater to remove harmful pathogens.	Government
**Government Factors**			
	National budgets, money, funding	The money or funding available (e.g., relating to public health and health care; research, surveillance, development and innovation; agricultural practices; etc).	
	Access to AMs outside of the system	Obtaining antimicrobials from alternative sources that are outside of the regulations of the healthcare system (e.g. without a prescription from a physician or veterinarian)	Individual
	Unregulated meat sales	The selling of meat product that have not gone through regulated processing and retail	Individual<br>Agriculture
**International Factors**			
	AMU in other countries	Amount of antimicrobials used in surrounding countries and countries that have been connected through travel	Individual<br>Trade
	Level of resistance in other countries	The estimate of the level of resistant organism or pathogens with resistant genes in other countries which can be connected through trade, travel, or geography	
	Movement of people	The physical movement of humans from one location to another (domestic and international)	Individual;

**CATEGORY THAT FACTORS CLUSTERED AROUND**	**NAME OF FACTORS INFLUENCING AMR**	**DEFINITION OF FACTOR**	**OVERLAPS WITH OTHER CATEGORIES (the factor applies to more than one category)**
			Public Health, Health Care and Social Care
	Movement of animals	The physical movement of wild and food-producing animals from one location to another (domestic and international)	Trade; Environment;
**Research, Surveillance, Development and Innovation Factors**			
	Science and academia	The research and scientific evidence done in the scientific and academic communities	
	Research, surveillance, development, and innovation	Work directed toward the innovation, introduction, and improvement of products and processes	
	Development of new AMs	The creation, development, and production of antimicrobials	
	Development of alternatives to AM	The creation, development, and production of any product that can be used instead of antimicrobials	
	New and emerging foods	New types of food which will enhance or replace current food practices	
	Diagnostics	All resources used to diagnose a disease in humans and animals	
	Corporate profits from AM	Pharmaceutical industry profits from selling antimicrobials	
	Pharmaceutical market, sales, and PR	The marketing and reputation of pharmaceutical companies and pharmaceutical representatives.	
