# Supplementary figures and images for: Factors influencing antimicrobial resistance in the European food system and potential leverage points for intervention: A participatory, One Health study
Source: PLoS One. 2022 Feb 22;17(2):e0263914. doi: 10.1371/journal.pone.0263914 (PMC8863257; doi:10.1371/journal.pone.0263914)

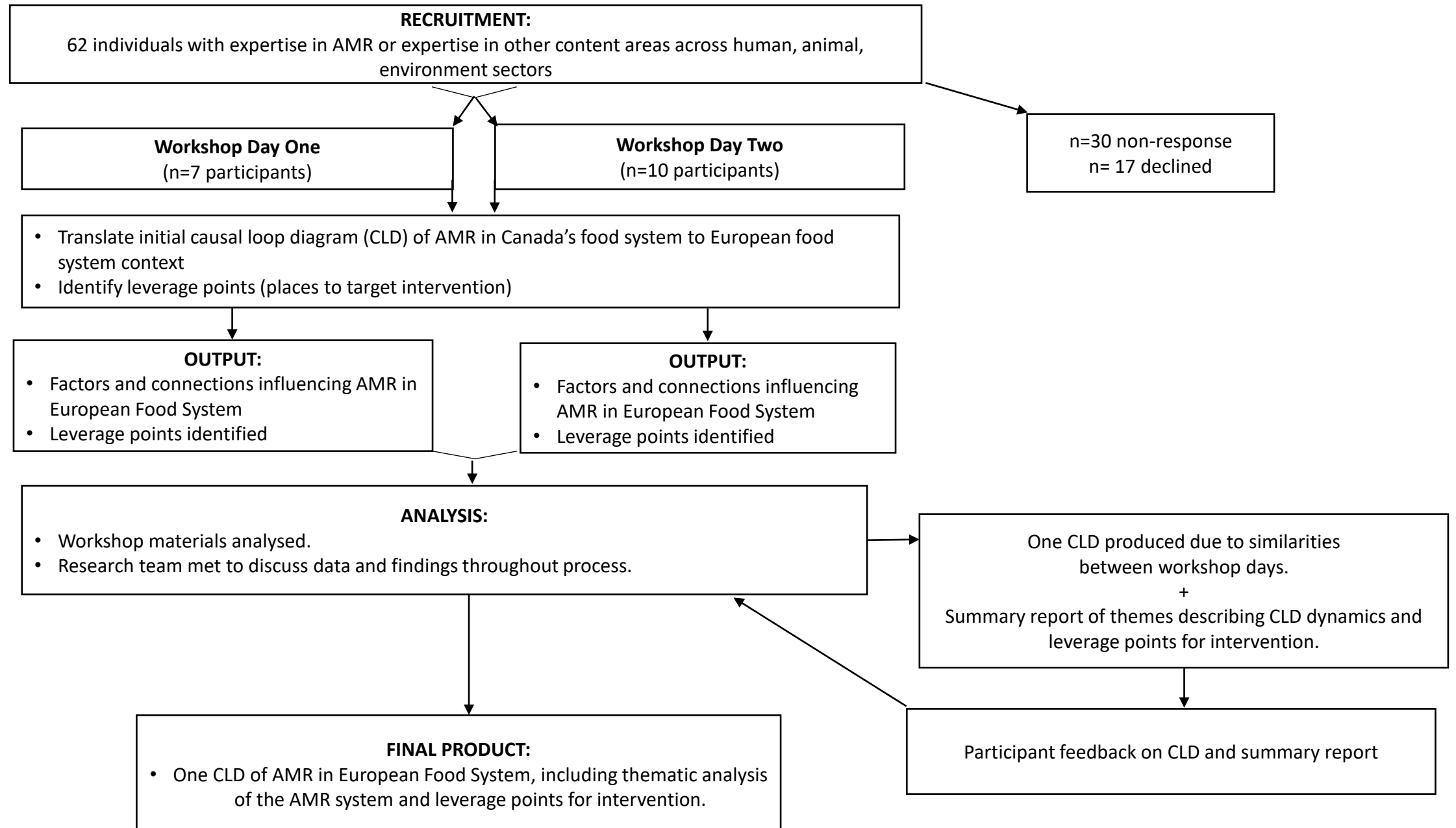

Supplement: S1 Fig — (PDF) [file pone.0263914.s005.pdf]

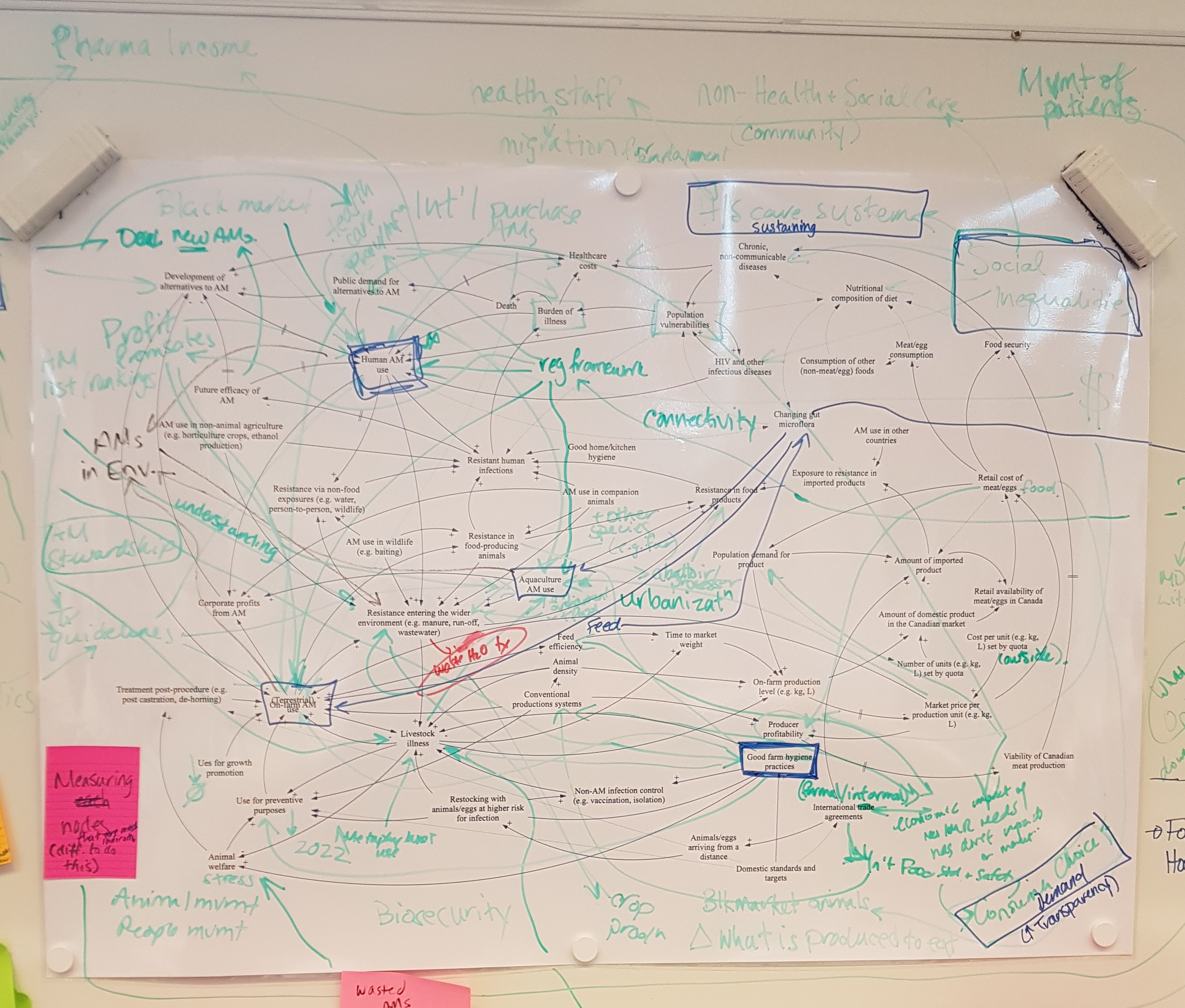

Supplement: S2 Fig — (JPG) [file pone.0263914.s006.jpg]

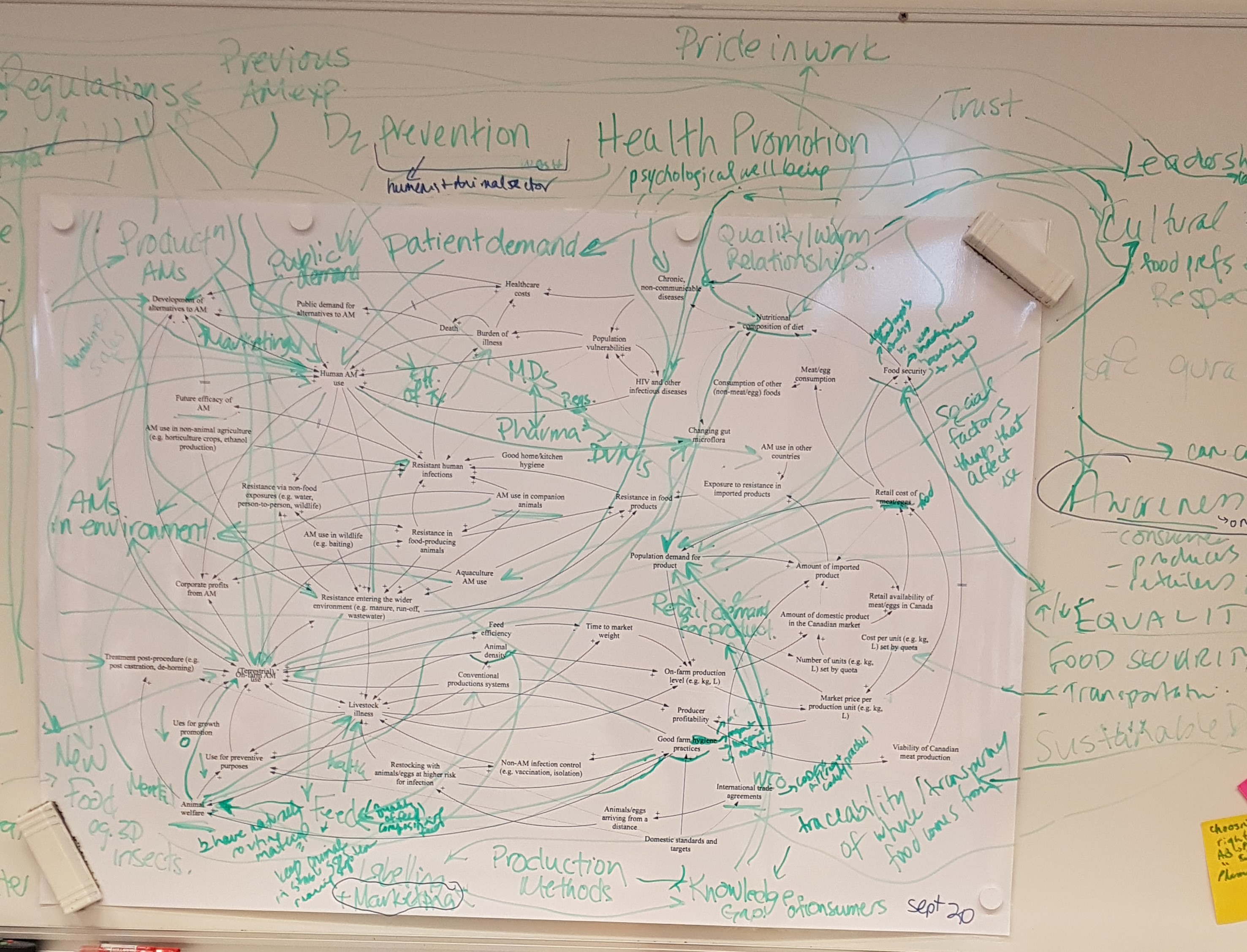

Supplement: S3 Fig — (JPG) [file pone.0263914.s007.jpg]

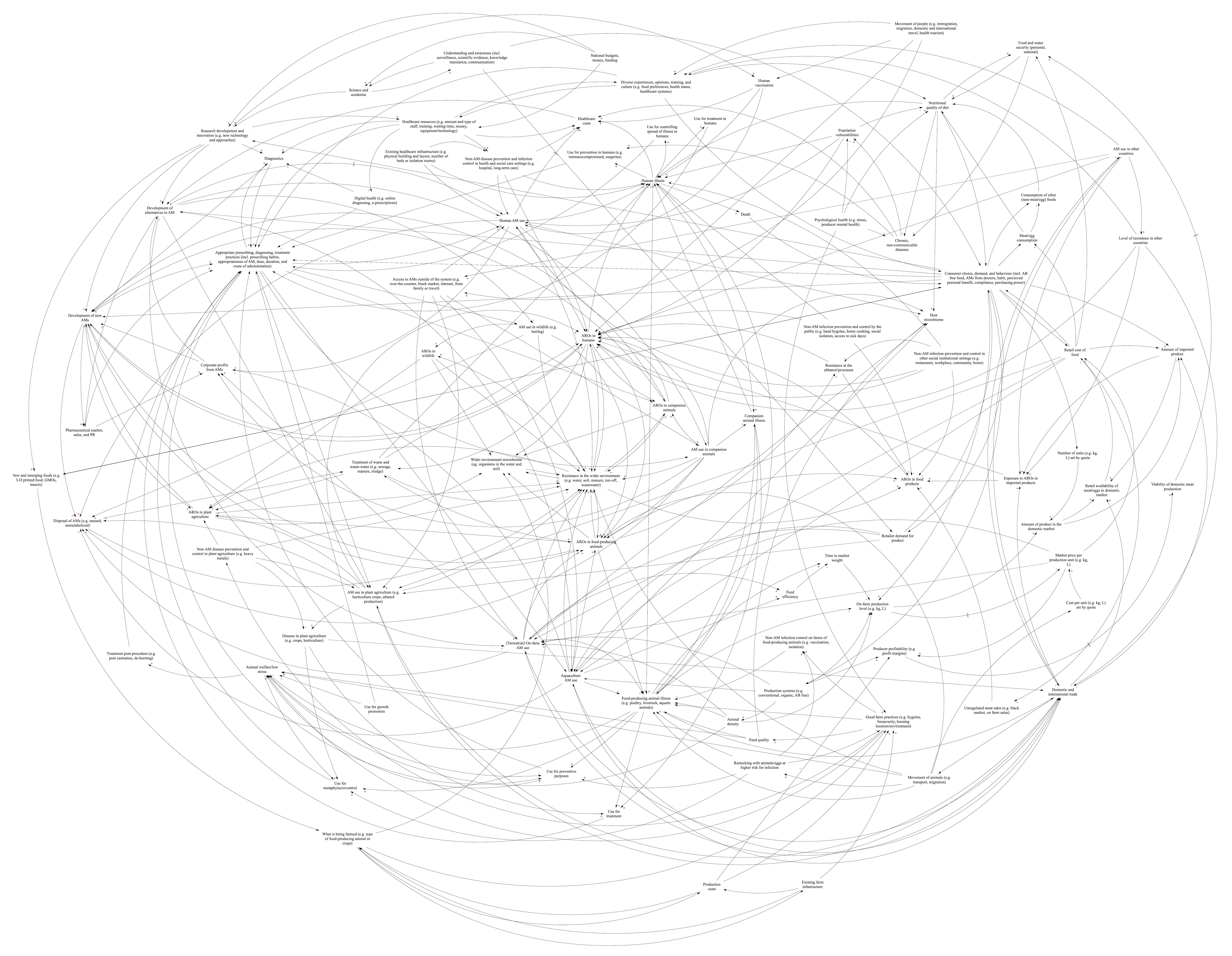

Supplement: S4 Fig — (JPG) [file pone.0263914.s008.jpg]
